# Investigation of cross-opsonic effect leads to the discovery of PPIase-domain containing protein vaccine candidate to prevent infections by Gram-positive ESKAPE pathogens

**DOI:** 10.1186/s12866-024-03427-w

**Published:** 2024-07-27

**Authors:** Océane Sadones, Eliza Kramarska, Diana Laverde, Rita Berisio, Johannes Huebner, Felipe Romero-Saavedra

**Affiliations:** 1 Division of pediatric infectious disease, Hauner children’s hospital, LMU, Munich, Germany; 2 Institute of Biostructures and Bioimaging, Italian Research Council (CNR), Naples, Italy

**Keywords:** Cross-reactivity, Antigen, PPIase, Enterococci, *Staphylococcus aureus*, Vaccines, ESKAPE pathogens, Opsonophagocytic assay

## Abstract

**Background:**

*Enterococcus faecium* and *Staphylococcus aureus* are the Gram-positive pathogens of the ESKAPE group, known to represent a great threat to human health due to their high virulence and multiple resistances to antibiotics. Combined, enterococci and *S. aureus* account for 26% of healthcare-associated infections and are the most common organisms responsible for blood stream infections. We previously showed that the peptidyl-prolyl cis/trans isomerase (PPIase) PpiC of *E. faecium* elicits the production of specific, opsonic, and protective antibodies that are effective against several strains of *E. faecium* and *E. faecalis*. Due to the ubiquitous characteristics of PPIases and their essential function within Gram-positive cells, we hypothesized a potential cross-reactive effect of anti-PpiC antibodies.

**Results:**

Opsonophagocytic assays combined with bioinformatics led to the identification of the foldase protein PrsA as a new potential vaccine antigen in *S. aureus*. We show that PrsA is a stable dimeric protein able to elicit opsonic antibodies against the *S. aureus* strain MW2, as well as cross-binding and cross-opsonic in several *S. aureus*,* E. faecium* and *E. faecalis* strains.

**Conclusions:**

Given the multiple antibiotic resistances *S. aureus* and enterococci present, finding preventive strategies is essential to fight those two nosocomial pathogens. The study shows the potential of PrsA as an antigen to use in vaccine formulation against the two dangerous Gram-positive ESKAPE bacteria. Our findings support the idea that PPIases should be further investigated as vaccine targets in the frame of pan-vaccinomics strategy.

**Supplementary Information:**

The online version contains supplementary material available at 10.1186/s12866-024-03427-w.

## Background

The ESKAPE pathogens *(Enterococcus faecium*,* Staphylococcus aureus*,* Klebsiella pneumoniae*,* Acinetobacter baumannii*,* Pseudomonas aeruginosa*, and *Enterobacter spp*) represent a great concern for global health. They were classified as a list of bacteria capable of “escaping” the effect of antibiotics [1]. Such behavior is a worldwide acknowledged threat, with the antimicrobial resistant bacteria responsible for 1.27 million deaths worldwide in 2019 with predicted rise in caused deaths to 10 million by 2050 [2]. The highest ranked Gram-positive bacteria in the World Health Organization (WHO) priority list of antibiotic-resistant bacteria are the two pertaining to the ESKAPE group: *E. faecium* and *S. aureus*. These bacteria are listed as “Priority 2: High”, based on several criteria, among which treatability, mortality, health-care burden, and trend to resistance [3].

Enterococci and *S. aureus* are part of the commensal microbiota in healthy individuals [4]. However, when the host-microbiota relationship becomes unbalanced, these opportunistic pathogens thrive and can induce life-threatening infections such as bacteremia or endocarditis [5–8]. In these cases, the duration of hospital stay increases, as well as the treatment costs and most importantly, the mortality rate [9]. *S. aureus* is the second most isolated pathogen involved in Hospital Acquired Infections (HAIs) between 2015 and 2017 in the United States (US). It represents 12% of the HAIs, while *E. faecalis* (7.9%) and *E. faecium* (3.8%) combined represent nearly 12% as well, which makes them the 5th and 8th most common pathogens isolated in the clinical setting [10]. *S. aureus* was identified as the most common pathogen responsible for bloodstream infections (BSI) (15%) and surgical site infections (SSI) (17.5%). *E. faecalis* ranked 4th in both the most frequently reported pathogens associated with BSI (7.6%) and Catheter-Associated Urinary Tract Infection (9.3%), as well as 3rd in the list of SSI (8%). While *E. faecium* was never found in the top 3 of each type of HAIs, 55.6% of *E. faecium* associated with SSI, and 82.1% of device-associated HAIs, were vancomycin-resistant. Also, not only do both pathogens thrive in clinical settings, being responsible for a great part of nosocomial infections but also, they are isolated from environmental reservoirs, threatening universal health care with community-acquired infections [11] and are involved in large outbreaks in hospitals [12, 13].

While the discovery of antibiotics remains one of the greatest advancements in medical science, clinically available antibiotic treatments often are ineffective to fight vancomycin-resistant enterococci (VRE) and methicillin-resistant *S. aureus* (MRSA), usually selecting resistant bacteria over sensitive ones [14]. In 2019, the Center for Disease Control and Prevention (CDC) published the “2019 Antibiotic Resistance Threats Report” which presents the latest data on deaths and infections by a list of 18 antimicrobial-resistant pathogens in the US. The report indicates a classification of such microorganisms, depending on the level of concern to human health. Both VRE and MRSA are listed as “Serious Threats” [15]. The CDC’s report accounts for more than 2.8 million of infections by antimicrobial-resistant pathogens and 35,000 associated deaths each year in the US, with VRE responsible of 5,400 (15%) and MRSA to blame for 10,600 deaths (30%) in 2017 [16, 17]. Great hope has been placed in yet another scientific discovery: vaccines. Prevention of bacterial infections can reduce the use of antibiotics, the cost of treatment and the length of hospitalization [9]. Numerous attempts to create either an enterococcal or staphylococcal vaccine led to the discovery of multiple vaccine antigen candidates, but so far no efficient and clinically available formulation has been introduced [18, 19].

Our team previously described the enterococcal peptidyl-prolyl cis-trans isomerase protein (PPIase), PpiC, as a promising antigen in *E. faecium.* We demonstrated that PpiC can induce the production of opsonic and cross-protective antibodies targeting different enterococci [20]. PPIases are ubiquitous in all types of cells, playing an essential role in protein folding, catalyzing the isomerization of peptide bonds. They are also known to be surface-exposed, involved in penicillin-binding protein folding, essential to bacterial growth, and involved in β-lactam resistance [21–23].

These characteristics led to the hypothesis that PpiC could be cross-reactive and cross-protective and may serve as a good antigen in other Gram-positive pathogens. Given the significant concern surrounding ESKAPE pathogens, we decided to focus on cross-reactivity between enterococci and *S. aureus*. In this study, we demonstrated that PpiC can induce opsonic antibodies that are cross-reactive between both Gram-positive pathogens. Bioinformatic analyses identified the staphylococcal protein PrsA as the closest homolog to PpiC, potentially explaining the cross-reactive activity observed with antibodies directed towards the enterococcal protein. Biophysical characterization and molecular modelling of PrsA demonstrated that the protein is a stable dimer with a bowl-like structure. We also show in this study that polyclonal antibodies elicited against this newly discovered antigen are cross-binding and cross-opsonic against enterococci and staphylococci.

## Methods

### Bacterial strains and culture conditions

The bacterial strains used in this study are presented in Table S1. *S. aureus* were grown in Brain Heart Infusion (BHI) plate at 37 °C or in BHI broth at 37 °C under agitation. We used Tryptic Soy Broth, and Agar to grow enterococci at 37 °C without agitation. For protein production, we used *Escherichia coli* M15, harboring pRep4, grown in Luria Bertani (LB) containing kanamycin at 25 µg/ml, at 37 °C with agitation. After transformation with pQE30, ampicillin at 100 µg/ml was added to the culture media.

### Bioinformatics and computational analysis

Sequence analysis to identify homologs of *E. faecium* PpiC in the *S. aureus* family was performed using BlastP database, by searching in the non-redundant sequence set of *S. aureus* (taxid 1280). No sequences could be retrieved in the *S. aureus* MW2 set (taxid 196,620), due to missing annotation of the bacterial strain. Indeed, once identified the homolog sequence set, we interrogated the *S. aureus* MW2 genome using a consensus sequence. This search uniquely identified PrsA from *S. aureus* MW2. Sequence alignment of PrsA against the sequence set of *S. aureus* (taxid 1280) confirmed that PrsA of *S. aureus* MW2 belongs to the identified set of PpiC homologs in *S. aureus* family. Sequence alignment between PpiC and PrsA from *S. aureus* MW2 was performed using EMBOSS Needle. Dataset of PpiC homologs with sequence coverage of more than 50% among *S. aureus* (taxid: 1280) was obtained using non-reduntant database and aligned using EMBL ClustalOmega [24]. The data were analyzed and visualized using JalView 2.0 programme [25]. JalView was also used to calculate residue consensus and conservation score. Conservation score is automatically calculated quantitative alignment annotation which measures the number of conserved physico-chemical properties conserved for each column of the alignment, based on the AMAS method of multiple sequence alignment analysis, whereas residue consensus presents the commonest residues and their percentage for each column of the alignment including gaps.

Also, a BlastP analysis was conducted to checked for potential homology between either PrsA or PpiC to proteins of human origin (taxid 9605).Molecular modelling of PrsA was conducted using Artificial Intelligence and employing the Colab server (https://colab.research.google.com/github/sokrypton/ColabFold/blob/main/AlphaFold2.ipynb), which predicts protein structures starting from their sequences, but applying a slightly simplified version of AlphaFold v2.0 (AF). The reliability of the AF predictions was assessed both by the Local Distance Difference Test (LDDT) score, a per-residue confidence score, with values greater than 90 indicating high confidence, and values below 50 indicating low confidence.

Pairwise sequence alignments were performed using EMBOSS Needle [26]. Antigenicity prediction was performed using Vaxijen tool [27]. These tools base their algorithms on principal amino acid properties of a protein sequence. Allergenicity was computed using AllergenFP v.1.0 [28]. Toxicity was computed with the ToxinPred, based on the recognition of residues detected in toxins through machine learning and quantitative matrix [29].

### Production of recombinant proteins

PpiC and PrsA were recombinantly produced in *E. coli* M15 harboring pRep4 and genetically engineered pQE30. The cloning, as well as the production and purification, was performed as described by Romero-Saavedra et al. (2015) [30]. Briefly, PrsA-encoding gene was amplified from the genomic DNA of *S. aureus* MW2 by PCR, excluding the signal peptide, using the primers listed in Table S2. Amplicons were added to pQE30 by genetic engineering. *E. coli* M15 containing pRep4 were transformed and selected for positive intake of the plasmid. To produce the recombinant proteins, bacteria were cultivated at 37 °C until optical density at 600 nm reached 0.5. At that stage, expression of the plasmid pQE30 was induced for two hours by addition of isopropyl β-D-1-thiogalactopyranoside at 0.5 mM. Bacteria were harvested, washed, and lysed enzymatically with lysozyme at 10 mg/ml and mechanically with glass beads. The protein of interest was isolated from the released pool of protein by affinity chromatography. The recombinant PrsA and PpiC were desalted and concentrated by diafiltration using the Amicon Ultra-15 Centrifugal Filter Units of 10,000 MWCO.

### CD and SEC-MALS analysis

CD spectra were recorded at 20 °C and 100 °C using a Jasco J-810 spectropolarimeter equipped with a Peltier thermostatic cell holder (Model PTC-423-S, Jasco, Italy). Far-UV measurements (195–260 nm) were carried out using a 0.1 cm path length cell with the protein at a concentration of 0.14 mg/mL in 20 mM sodium phosphate buffer, pH 7.5. CD spectra were averaged over three independent scans. Thermal denaturation was performed from 20 to 100 °C with an increment of temperature of 1 °C/min. Temperature midpoint was analysed using Boltzman sigmoidal fit in Origin software. The unfolding process was monitored recording the CD signal at 222 nm. Molar ellipticity per mean residue, [θ] in deg cm^2^‧dmol^− 1^, was calculated from the equation: [θ] = [θ]obs‧MRW‧(10‧l‧C)-1, where [θ]obs is the ellipticity measured in degrees, MRW is the mean residue molecular mass, C is the protein concentration in g/l and l is the optical path length of the cell in cm. Mean residue molecular mass is calculated from MRW = M/(N-1), where M is the molecular mass of the protein (in Dalton) and N is the number of amino acids.

Light scattering measurements were performed using size exclusion chromatography (Superdex 200 pg Increase 10/300, Cytiva, Italy) coupled with a light scattering detector (miniDAWN TREOS, Wyatt Technology Corporation, CA, US) and to a differential refractive index detector (Shodex RI-101, Wyatt Technology Corporation, California, US). The measurements were carried out loading ~ 1 mg of the protein sample on the column equilibrated in 50 mM Tris–HCl, 150 mM NaCl, 2.5% (v/v) glycerol (pH 7.8) as running buffer, at a flow rate of 0.4 ml/min. Data were recorded and analysed using the Astra software (version 5.3.4, Wyatt Technology Corporation, California, US).

### Rabbit immunization with recombinantly produced proteins

Recombinant PrsA was used to produce polyclonal sera in New Zealand rabbits by the company BioGenes GmbH (Germany), which conforms to European animal welfare regulations, regulating ethical issues on laboratory animal treatment. Immunization work at BioGenes GmbH was under approval from National Institutes of Health (NIH)/ Office of Laboratory Animal Welfare (OLAW) (ID number #A5755–01).

Rabbits were selected before immunization by assessing their level of pre-existing opsonic antibodies. Later, they were immunized with four subcutaneous injections, all containing protein and a proprietary adjuvant from Biogenes. At day 1, pre-immune serum was collected, and the animals received 200 µg of pure protein. Following that first immunization, they received three boosts with 100 µg of protein at day 7, day 14 and day 42. Anti-protein sera were collected on day 49. Euthanasia was carried out in combination of bolt shot (mechanical stunning; NPCB) and final bleeding from the carotis of the animal. The animals are fixed between the knees of the trained operator. The bolt shot (NPCB) is applied on the back of the head, behind the eyes. Immediately after the shot, the animal is hung upside down by its legs by a second supporting operator and the throat cut is performed (final bleeding from carotis). Ketamin and Xylazin is used as anesthetics. The collected sera were heat-inactivated at 56 °C for 30 min and then stored frozen at -20 °C. Table S3 lists the different rabbit sera used in this study.

### Immunodetection of specific antibodies by ELISA

ELISA were performed as previously described by Romero-Saavedra at al. (2019) [31]. Briefly, the recombinant proteins were used at 1 µg/ml to coat Nunc-immuno MaxiSorp 96-well plates overnight. After washing, plates were blocked for one hour with PBS-BSA (phosphate buffer saline with 3% of Bovine Serum Albumin). Sera were adjusted at different concentrations ranging from 60 to 15 µg/ml for cross-binding studies and 1.8 to 0.45 µg/ml for specificity studies in PBS-BSA, added to the plate and incubated for one hour. After washing, alkaline phosphatase conjugated anti-rabbit immunoglobulin G produced in goat was diluted to 1:1000 and incubated for one hour. Finally, wells were washed five times and Ortho-nitrophenyl-b-D-galactopyranoside at 1 mg/ml was added to the plates. Absorbance was read after two hours at 405 nm.

### Opsonophagocytic killing assay

In vitro opsonophagocytic assays were performed as described elsewhere [32, 33]. The four components were prepared as followed: (a) the pre- and anti-protein sera were heat inactivated (HI) by incubation at 56 °C for 30 min and diluted to the desired concentration in RPMIF (Roswell Park Memorial Institute 1640 medium with 15% HI fetal bovine serum), (b) the lyophylised baby rabbit complement (Cedarlane Laboratories) was resuspended in RPMIF and diluted at dilutions of 1:30 for use with *S. aureus* strains and 1:15 for enterococci. Potentially present IgGs were removed by incubation with an excess amount of the tested strain for one hour at 4 °C under light agitation. After adsorption, the complement was centrifugated and filtrated to be used in the assay, (c) White blood cells (WBC) were freshly isolated from healthy donors. 25 ml of fresh blood was incubated for 45 min at 37 °C with 25 ml of heparin-dextran buffer (4.5 g NaCl, 10 g dextran, 32.8 mg heparin-sulfate sodium salt in 500 ml injectable water). The upper phase was collected, centrifuged and washed in RPMIF. Lysis of red blood cell was performed by incubating the cells in a 1% ammonium chloride solution for 20 min. After centrifugation and subsequent wash, the remaining white blood cells were adjusted to a concentration of 18.10^6^ cells/ml in RPMIF, (d) bacteria were grown until OD600 nm reached 0.4. After collection by centrifugation and a wash, the bacterial cells were diluted in RPMIF. All components were incubated together for 90 min at 37 °C under light agitation. The suspension was diluted and plated on agar plates, grown overnight and counted the next day. Percentages of killing were calculated by comparing the number of colony-forming units obtained after incubation with or without white blood cells (WBCneg) using the following formula: {[(mean CFU WBCneg at t90) – (mean CFU at t90)]/(mean CFU WBCneg at t90)} x 100. Every assay was conducted with four controls to confirm how each biological material behaved in the relevant experiment. The number of colonies recorded for every control was contrasted with the number of colonies isolated from a sample of bacteria incubated in the absence of any additional agent. Three negative controls evaluated: complement-mediated phagocytosis (tubes containing only complement and PMNs), complement-associated lysis (tubes containing only complement), and WBC sensitivity. Commercially available intravenous immunoglobulin preparations (Intratect, Biotest) was used as a positive control.

### Quantification and statistical analysis

Data were statistically compared using the software GraphPad PRISM version 5.00. For in vitro experiments, the unpaired two-tailed T-test with a 95% confidence interval was used. Bars and whiskers represent mean values ± SEM. NS, not significant (*P* > 0.05). *P ≤ 0.05, ** *P* ≤ 0.01, *** *P* ≤ 0.001.

## Results

### Serum of rabbit immunized with enterococcal protein PpiC contains *S. aureus*-opsonic antibodies

**Fig. 1:**
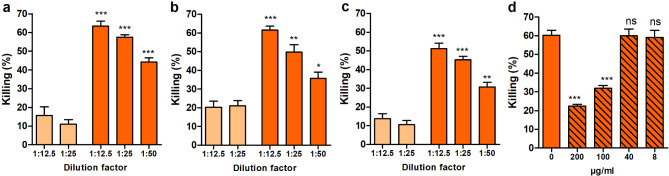
Cross-opsonic activity of anti-PpiC in various *S. aureus* strains. Opsonophagocytic assay against *S. aureus* (**A**) MW2, (**B**) LAC, and (**C**) Reynolds. Strains were tested for opsonic killing mediated by different dilutions of sera, anti-PpiC (orange) obtained from a terminal bleed of a rabbit immunized with PpiC, and pre-PpiC (light orange), pre-immune serum drawn from the rabbit before immunization. Statistical differences between pre- and anti-PpiC at similar concentrations were tested by unpaired two-tailed T-test with a 95% confidence interval. Bars and whiskers represent mean values ± standard error of the mean (SEM). NS, not significant (*P* > 0.05). *P ≤ 0.05, ** *P* ≤ 0.01, *** *P* ≤ 0.001. (**D**) Opsonophagocytic inhibition assay of anti-PpiC against *S. aureus* MW2. Antibodies diluted to 1:25 were incubated with the purified PpiC at concentrations ranging from 200 µg/ml to 8 µg/ml (diagonal stripes). Opsonic killing observed was compared to the positive control, anti-PpiC at 1:25 (orange). Statistical differences were calculated by unpaired two-tailed T-test with a 95% confidence interval. Bars and whiskers represent mean values ± SEM. NS, not significant (*P* > 0.05), *** *P* ≤ 0.001

The peptidyl-prolyl cis-trans isomerase PpiC has been previously identified as a valuable antigen to prevent enterococcal infections [20]. Previous findings also suggest that PPIases are ubiquitous among different species and essential for the growth of Gram-positive bacteria [22, 23]. Therefore, we decided to test whether rabbit serum raised against PpiC can mediate opsonophagocytic killing of *S. aureus*. Polyclonal serum was previously obtained by immunization of New Zealand rabbit with the recombinant protein PpiC from *E. faecium* E155. After several injections with the antigen, the blood was drawn, and serum was isolated [20]. Three different *S. aureus* strains: MW2, LAC, and Reynolds were tested by Opsonophagocytic assay (OPA) against the anti-PpiC serum. For each strain, the results show a significant increase in the percentage of killing when comparing rabbit sera collected before (pre-) and after immunization with PpiC (anti-PpiC). Anti-PpiC antibodies mediated a concentration-dependent opsonophagocytic killing up to 65% at the lowest tested dilution (1:12.5), which was significantly higher when compared to the pre-immunize sera (Fig. 1ABC). To verify that the observed killing was due to the presence of PpiC-specific antibodies, opsonophagocytic inhibition assays were performed against the *S. aureus* strain MW2. The polyclonal antibodies were incubated with different concentrations of the recombinantly produced PpiC overnight and used in following steps of the assay. Results presented in Fig. 1D show that killing mediated by opsonization with anti-PpiC was inhibited by incubation with recombinantly produced PpiC in a dose-dependent manner. This indicates that the antibodies directed against PpiC in the serum are responsible for the opsonic killing observed against the tested *S. aureus* strains. These results led us to hypothesize that a staphylococcal protein should share one or several epitopes with PpiC.

### PrsA is bioinformatically predicted to be the antigen responsible for cross-reactivity with *E. faecium*

The ability of the anti-PpiC serum to mediate the killing of different *S. aureus* strains, suggested that *S. aureus* possesses a homologous protein, recognized by anti-PpiC antibodies. BlastP analysis against non-redundant protein sequences of *S. aureus* (taxid:1280) identified 412 sequences (> 50% sequence coverage), sharing with PpiC of *E. faecium* sequence identities between 26 and 29%. The identified sequence set describes parvulin-like PPIase, denoted as PrsA in *S. aureus*. Consistent with the restricted range of sequence identities of these identified proteins with PpiC of *E. faecium*, we observe an almost full sequence conservation in the *S. aureus* family (96–100%, figure S1 and S2). Although a direct search for homologs using the *S. aureus* MW2 set (taxid: 196,620) produced no results, due to missing annotation of the bacterial strains in the sequence set, the interrogation of the *S. aureus* MW2 genome using a consensus sequence from the *S. aureus* dataset (taxid:1280) uniquely identified PrsA from *S. aureus* MW2, making this protein the best candidate to explain the cross-opsonic activity observed for anti-PpiC sera.

Aiming at the development of vaccine antigens, we also checked whether PrsA and PpiC share any homology with human proteins. In this case, BlastP analysis against non-redundant protein sequences of human origin (taxid 9605) produced no significant homology with either PrsA of *S. aureus* or PpiC of *E. faecium.*

### *S. aureus* PrsA is a stable dimeric protein

**Fig. 2:**
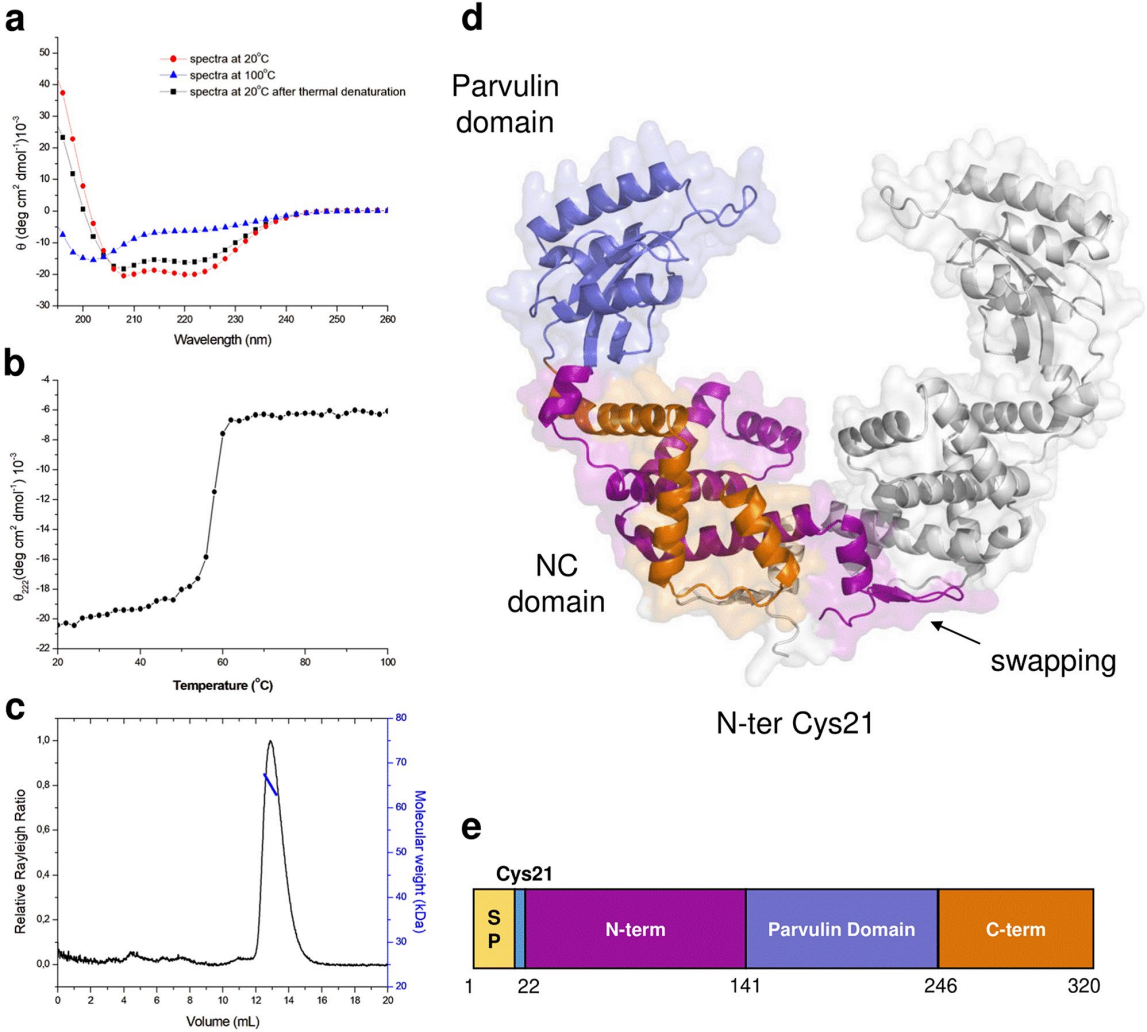
Biophysical characterization of PrsA. (**A**) CD spectra of PrsA in 20 mM sodium phosphate, pH 7.4. Spectra are reported for measurements at 20 °C (red), at 100 °C (blue) and after refolding at 20 °C (black). (**B**) thermal denaturation curve measured at 222 nm. (**C**) Analytical SEC-MALS of PrsA. The black curve represents the Rayleigh ratio (left scale) against the retention volume. (**D**) Structural model of PrsA as computed using AlphaFold v2.0. (**E**) Structure-based domain boundaries in PrsA

The identified gene encoding for the protein PrsA was amplified from the *S. aureus* MW2 genome and cloned into an expression plasmid to recombinantly produce the protein in *Escherichia coli*. A structural characterization of PrsA in solution, using Circular Dichroism (CD) spectroscopy, shows a high content of α-helical secondary structure (Fig. 2A). Indeed, CD spectra present a maximum near 190 nm and two minima at 208 and 222 nm (Fig. 2A), with an almost reversible denaturation process. Thermal unfolding, analyzed by recording the CD signal at 222 nm, shows a cooperative transition corresponding to a melting temperature (Tm) of 57.2 ± 0.2 °C (Fig. 2B). Analysis of PrsA oligomerisation state, performed using size exclusion chromatography coupled to light scattering (SEC-MALS, Size Exclusion Chromatography-Multi Angle Light Scattering), unambiguously revealed a dimeric state of the protein (Fig. 2 C), showing a molecular mass of 65.5 ± 0.1 kDa. Using this information, we modeled PrsA structure using artificial intelligence and the program AlphaFold v2.0 in a template mode. The resulting highly reliable structure (pLDDT > 90 for the protein region S28-N302) is a symmetric bowl-shaped dimer, that present swapping of their N-terminal regions (residues 21–51). Each chain formed by two domains, the catalytic parvulin domain and an NC domain (Fig. 2D). Like PpiC of *E. faecium*, PrsA sequence (320 residues) contains an N-terminal cysteine (Fig. 2DE) that is enzymatically modified upon signal sequence cleavage with a diacylglycerol residue for membrane association. Following the signal peptide, PrsA contains three regions: a large N-terminal region, a parvulin PPIase domain and a small C-terminal region (Fig. 2E). In each chain, the parvulin domain consists of a four-stranded antiparallel β-sheet surrounded by four α-helices typical of parvulin-type PPIase domains (S141-P246) [34], whereas the NC domain is almost fully α-helical, and formed by N-terminal (G51 -D140 and the swapping C21-I50 of the adjacent chain) and the C-terminal (T247-N302) regions (Fig. 2D).

### Anti-PpiC rabbit serum binds to the recombinantly produced PrsA

**Fig. 3:**
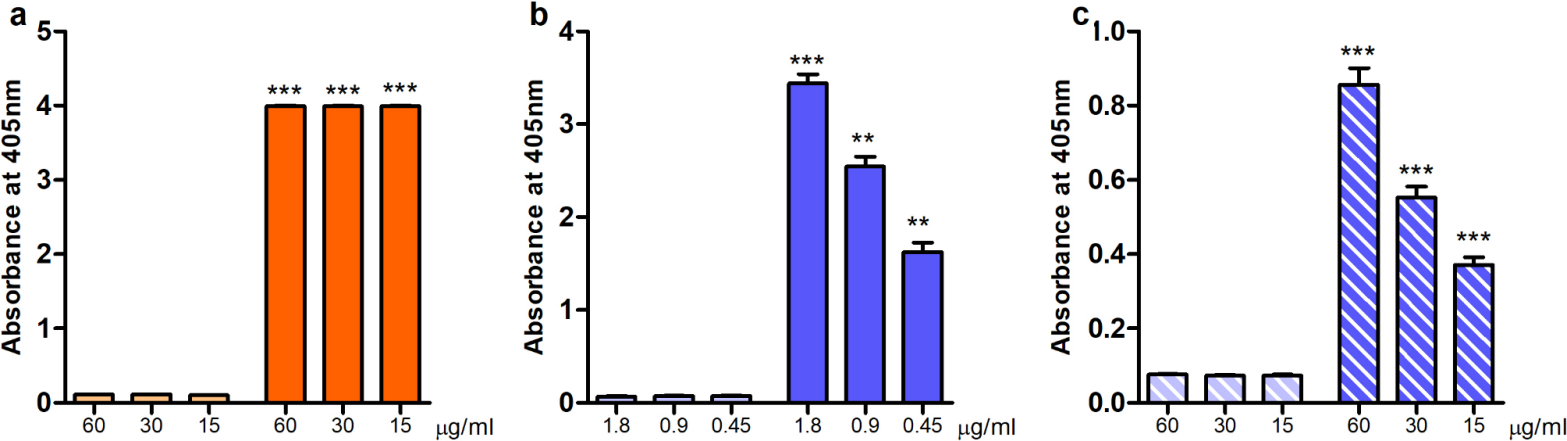
Immunoreactivity of the different anti-protein sera. (**A**) The binding of pre- and anti-PpiC rabbit sera to the recombinantly produced PrsA was assessed by ELISA. Nunc-Immuno MaxiSorp 96-well plates were coated with PrsA and incubated with various concentrations of either pre- (light orange) or anti-PpiC sera (orange). (**B**) Specific antibodies towards the staphylococcal protein PrsA in pre- (light blue) and anti-PrsA (dark blue) were assessed following the same ELISA protocol. (**C**) Cross-immunoreactivity against recombinantly produced PpiC against pre- (light blue - diagonal stripes) and anti-PrsA sera (dark blue – diagonal stripes) were determined as described above but using the recombinantly produced protein PpiC to coat the plates. Values for the same dilution were statistically compared using an unpaired two-tailed T-test with a 95% confidence interval. Bars and whiskers represent mean values ± SEM. ** *P* ≤ 0.01, *** *P* ≤ 0.001

To biologically assess the involvement of PrsA in the cross-reactivity effect seen with anti-PpiC serum, the purified PrsA protein was used to coat Nunc-Immuno MaxiSorp 96-well plates. Both sera collected before and after immunization of rabbits with PpiC were added to the plates to test for cross-binding to the hypothesized homologous protein. The results presented in Fig. 3A indicate that anti-PpiC serum contains antibodies binding to the staphylococcal protein PrsA, as opposed to the serum collected before immunization with PpiC. The results suggest that polyclonal serum, raised by immunization of rabbits with PpiC, and containing PpiC-specific antibodies, can also bind to PrsA. These data support the bioinformatics prediction of the protein PrsA as a good candidate to explain the cross-reactivity of anti-PpiC against *S. aureus*.

### Immunization of rabbits by the recombinant protein PrsA leads to the production of antibodies that recognize both PrsA and PpiC

We decided to further investigate the role of PrsA in the cross-opsonic effect observed with anti-PpiC. Polyclonal antibodies were raised in New Zealand rabbits: after collection of pre-immune serum (pre-PrsA), the recombinantly produced and purified protein was injected following the immunization schedule detailed in the method section. On day 49, rabbits were exsanguinated, and the serum was collected. The obtained anti-PrsA serum was tested by Enzyme-Linked ImmunoSorbent Assay (ELISA) for the presence of PrsA-specific antibodies. A significant increase of antibodies binding to PrsA is observed in the serum elicited after immunization. This result presented in Fig. 3B shows that anti-PrsA contains specific antibodies able to recognize the target protein. The immunization led to the production of specific antibodies, suggesting the immunogenic profile of the protein. Anti-PrsA was also tested for cross-binding to PpiC. Nunc-Immuno MaxiSorp 96-well plates were coated with recombinantly produced PpiC overnight and then incubated with pre- and anti-PrsA. Binding of the anti-protein serum to PpiC was significantly higher while compared to similar dilutions of the pre-immune serum (Fig. 3C). Immunization with PrsA led to the production of antibodies that are able to recognize both the enterococcal PPIase PpiC and the staphylococcal PrsA. These results strengthen our hypothesis that PrsA is the homologous protein in *S. aureus* responsible for the cross-reactivity of anti-PpiC against both Gram-positive strains. It also confirms that PrsA and PpiC share epitopes, targets of antibodies binding to both proteins.

### Pre-incubation of anti-PpiC with the protein PrsA leads to an inhibition of the opsonophagocytic killing

**Fig. 4:**
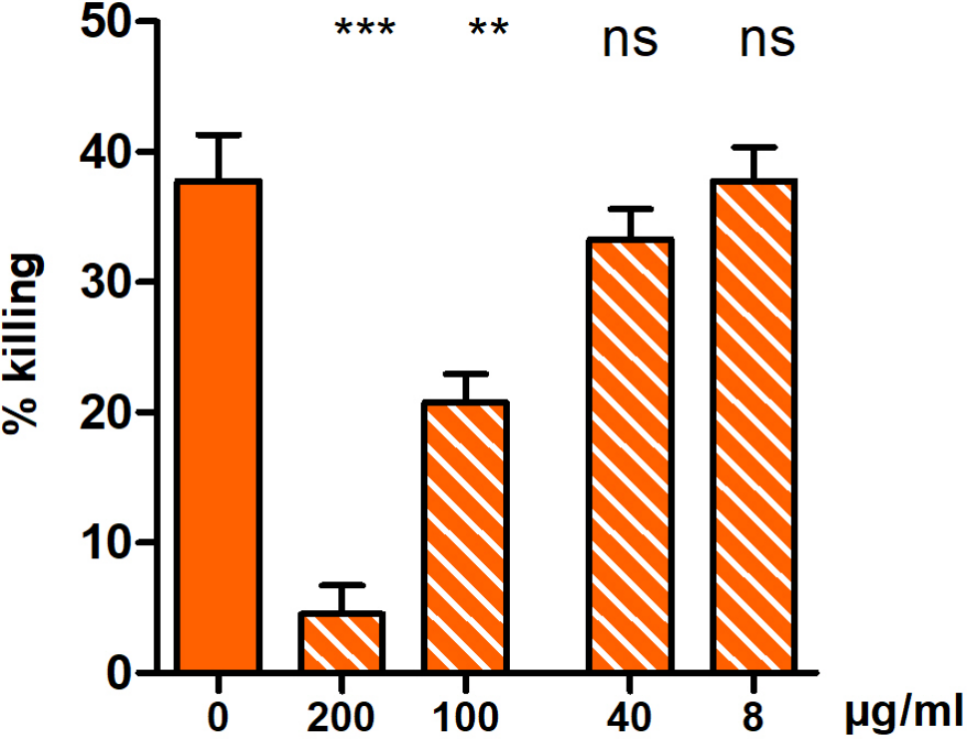
Inhibition of the opsonophagocytic killing of anti-PpiC by incubation with PrsA. OPiA against *S. aureus* MW2 was performed using the antibodies present in anti-PpiC diluted at 1:20 and previously incubated with PrsA. Anti-PpiC at 1:25 (orange) was mixed with several concentrations of PrsA, ranging from 8 to 200 µg/ml (orange – vertical stripes). After an overnight incubation, the inhibited sera were used in OPA. The killing obtained with no inhibitors (0 µg/ml) and the killings seen with each concentration (8–40 – 100 – 200 µ/ml) were statistically compared using an unpaired two-tailed T-test with a 95% confidence interval. Bars and whiskers represent mean values ± SEM. NS, not significant (*P* > 0.05), *** *P* ≤ 0.001

As seen in Fig. 3, antibodies raised against PpiC can bind to PrsA, as well as anti-PrsA to PpiC. The cross-binding effect of both anti-protein sera to their homologs confirms that the proteins share epitopes. However, to confirm that PrsA is responsible for the cross-reactivity observed in Fig. 1, we decided to perform an opsonophagocytic inhibition assay. Briefly, anti-PpiC was incubated overnight with a ranging concentration of PrsA (8 to 200 µg/ml). The next day, those potentially inhibited sera were tested following the same protocol as for a classical OPA. The results presented in Fig. 4 indicate that the overnight incubation of anti-PpiC with PrsA greatly reduced the killing mediated by the antibodies with no inhibitors. The decrease in killing is dose-dependent and show a statistical difference when compared to the uninhibited serum. The data show that PrsA is recognized by the opsonic antibodies present in anti-PpiC. The results strongly suggest that PrsA is the protein in *S. aureus* responsible for the cross-reactive opsonic killing mediated by the antibodies raised against the enterococcal protein PpiC.

### Immunization with PrsA induces the production of opsonic and cross-reactive antibodies

**Fig. 5:**
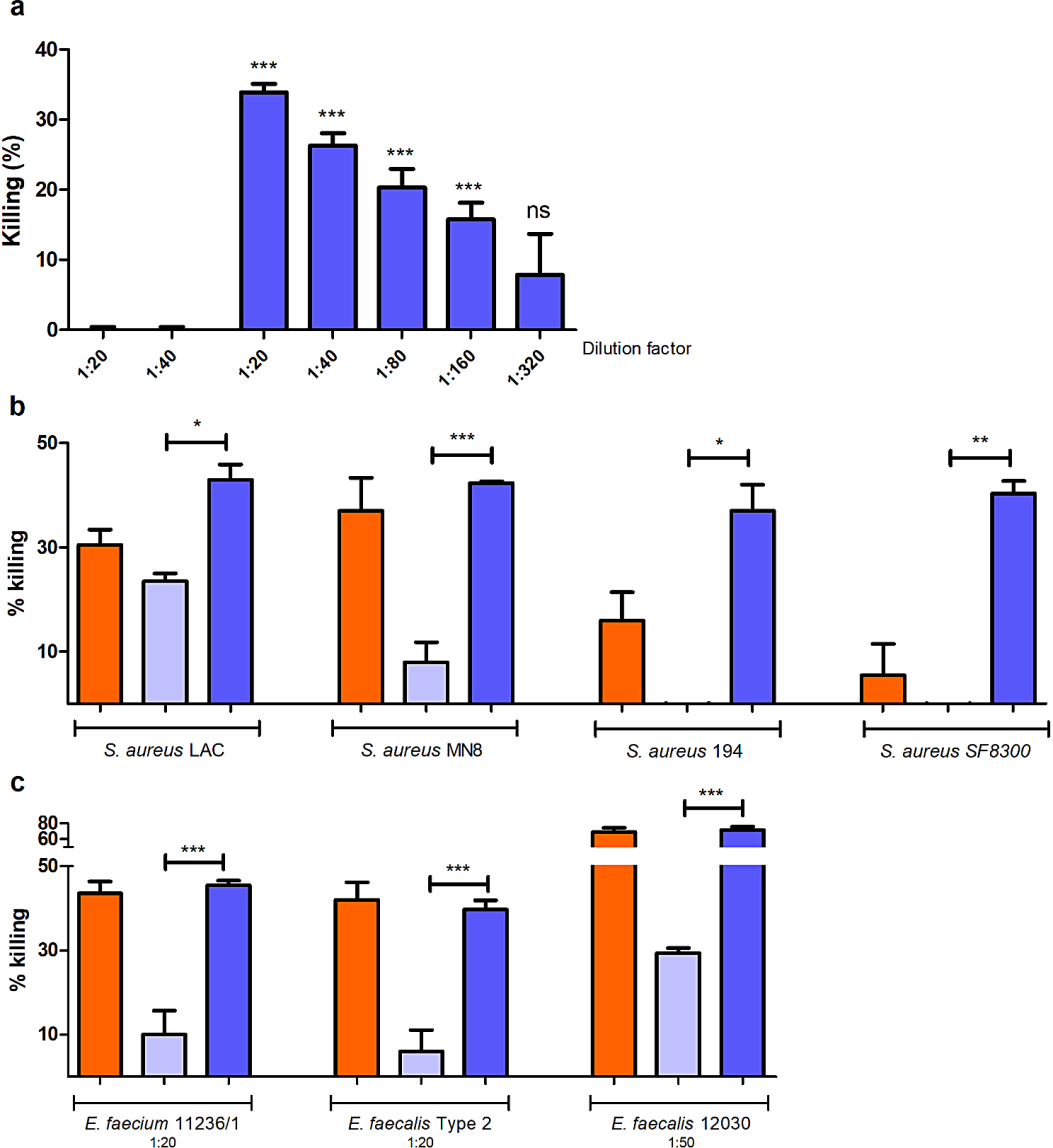
Opsonophagocytic killing activity of anti-PrsA. (**A**) *Staphylococcus aureus* MW2 was tested for killing mediated by opsonic antibodies-containing anti-PrsA (dark blue) and pre-PrsA was used as negative control (light blue). (**B**) Killings mediated by antibodies in anti-PpiC (orange), pre- (light blue) and anti-PrsA (dark blue) were also assessed against several *S. aureus* strains. Sera were diluted at 1:20 for all the four strains tested: LAC, MN8, 194 and SF8300. (**C**) Three enterococcal strains were also tested with anti-PpiC as well as pre- and anti-PrsA. Sera were diluted at 1:20 for use in OPA against *Enterococcus faecium* 11236/1 and *Enterococcus faecalis* Type 2, and at 1:50 while tested against *Enterococcus faecalis 12030*. Statistical differences between pre- and anti-PrsA at the same dilution were determined using unpaired two-tailed T-test with a 95% confidence interval. Bars and whiskers represent mean values ± SEM. NS, not significant (*P* > 0.05). *P ≤ 0.05, ** *P* ≤ 0.01, *** *P* ≤ 0.001

To investigate the potential opsonic activity of antibodies elicited by the immunization with PrsA, pre- and anti-PrsA rabbit sera were tested in OPA against *S. aureus* MW2 (Fig. 5A). A significant increase in antibodies-mediating killing was observed. A wide range of concentration was tested to titer out the opsonic activity. At a serum dilution of 1:20, 35% killing was observed against *S. aureus* MW2. The killing behaved in a dose-dependent manner and could be almost entirely abolish at 1:320 dilution. The capacity of polyclonal antibodies directed towards PrsA to mediate opsonic killing of other *S. aureus* strains was assessed to check for cross-opsonic activity (Fig. 5B). Also, as the previous data showed cross-binding of anti-PrsA and anti-PpiC to their respective identified homologous, and that anti-PpiC presents with opsonic antibodies against a *S. aureus* strain MW2, rabbit sera were tested against various enterococcal strains (Fig. 5C). Antibodies raised by immunization with PrsA mediated a significantly higher percentage of killing when comparing pre-immune serum. In every strain tested, killing mediated by antibodies contained in anti-PpiC was either equal or slightly lower as compared to the killing obtained by mediation of anti-PrsA antibodies. Antibodies raised against PrsA can mediate the opsonic killing of both enterococci and *S. aureus*.

### Shared high-antigenicity regions in PpiC and PrsA

**Fig. 6:**
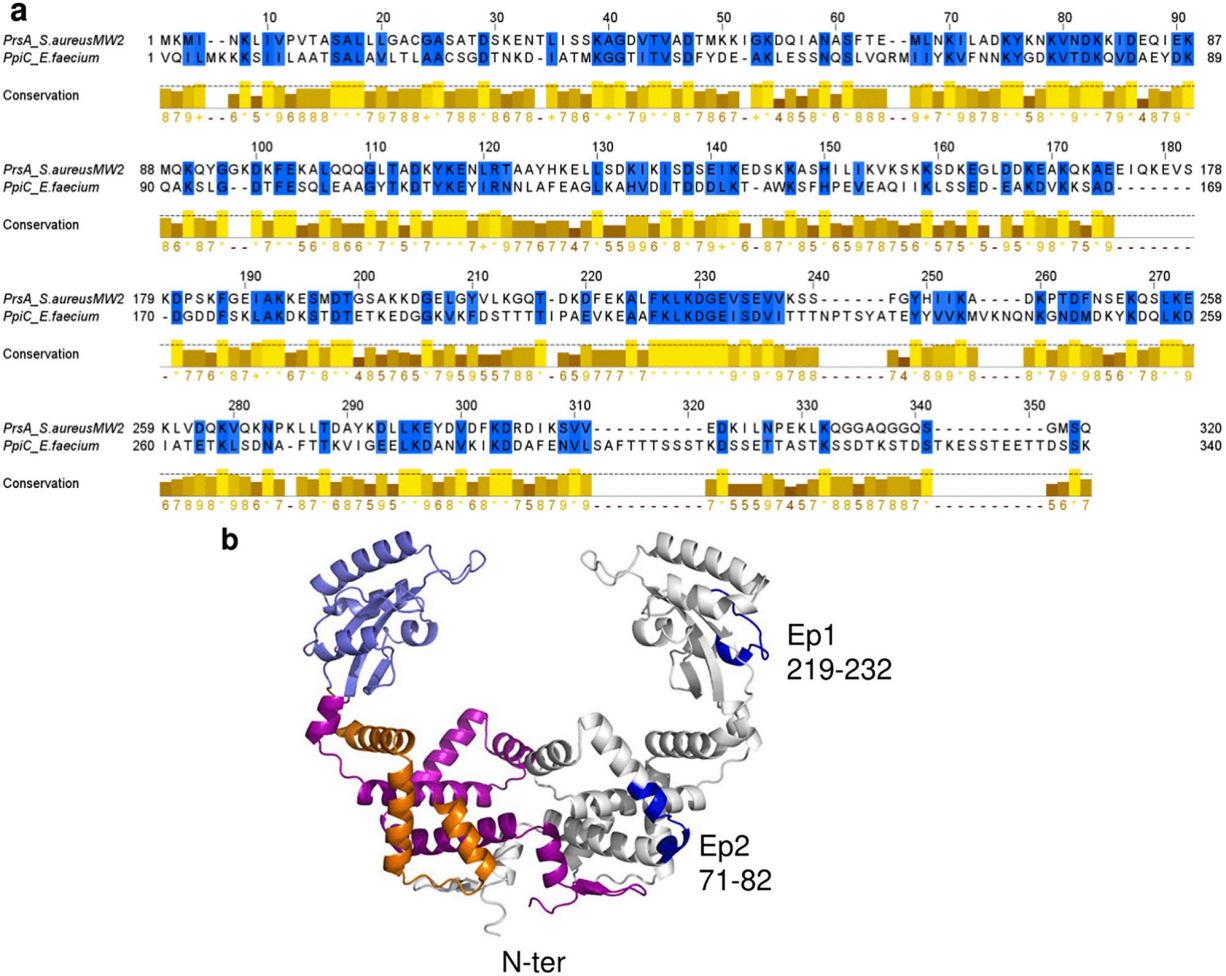
Prediction of possible epitopes responsible for cross-reactivity. (**A**) Pairwise sequence alignment, performed using EMBOSS Needle. (**B**) Mapping of the two identified regions 219-ALFKLKDGEVSEVV-232 (Ep1) and 71-KYKNKVNDKKID-82 (Ep2) on PrsA model structure. Ep1 and Ep2, drawn in blue on the right grey chain, are located on the parvulin and the NC domain, respectively

In an attempt to identify molecular determinants of the observed cross-reactivity, we first performed pairwise sequence alignment (Fig. 6). The overall pairwise sequence identity of the two proteins, computed using EMBOSS Needle is 24.9%, with the strongest sequence differences located in their C-terminal regions (Fig. 6A). However, strongly conserved regions are observed between residues 219–232 (210–223 in PpiC, seqid – 71.4%, similarity 92.9%), followed by the region 71–82 (73–84 in PpiC, seqid 58.3%, similarity 83.3%) (Fig. 6A; Table 1). These regions are located on the parvulin domain and in the NC domains, respectively (Fig. 6B). Antigen prediction was performed on the whole PrsA and PpiC sequences and the specific conserved sequences, using Vaxijen (Table 1). Consistent with our experimental results, both proteins are predicted to be strong antigens, with high antigenicity scores according to Vaxijen (0.77 and 0.86, respectively) (Table 1). Significantly stronger antigenicity scores characterize the two identified sequence regions (Table 1) in both PrsA and PpiC (Table 1). AllergenFP v1.0 and ToxinPred were used to assess the putative allergenicity and toxicity, respectively (Table 1).

**Table 1:** Prediction of antigenicity, allergenicity and toxicity of PrsA and PpiC sequences. “-” stands for either non-toxic or non-allergenic, “+” for either toxic or allergenic

Sequence	Vaxijen score (threshold 0.40)	Toxicity	Allergenicity
Full length PpiC	**0.86**	-	-
Full length PrsA	**0.77**	-	-
PpiC 210–223 (AAFKLKDGEISDVI)	**1.21**	-	+
PrsA 219–232 (ALFKLKDGEVSEVV)	**1.06**	-	-
PpiC 73–84 (KYGDKVTDKQVD)	**1.67**	-	-
PrsA 71–82 (KYKNKVNDKKID)	**1.92**	-	-

## Discussion

Gram-positive ESKAPE pathogens represent a great threat due to their prevalence among hospitals with vulnerable patient populations [1, 14, 35]. Together, enterococci and *S. aureu*s account for 35% of surgical site infections, and 26% of all types of healthcare-associated infections reported in the US between 2011 and 2014 [36]. These pathogens are also the most common organisms causing nosocomial blood stream infections [35, 36]. Given their ability to induce life-threatening infections and to develop antibiotic resistances, preventive treatments are urgently needed. Not only do effective vaccines prevent infections, but also they are associated with the reduction of antibiotic use, cost of treatment and length of hospitalization, making them a great asset for our society [9, 37, 38]. Unfortunately, despite the numerous attempts, as well as the great number of discovered antigens, so far no vaccines have been successfully developed to prevent infections by either enterococci or *S. aureus* [18–20, 30, 31, 39–42].

In a previous study, our team presented the PPIase, PpiC from *E. faecium* as a promising vaccine antigen against several strains of *E. faecium* and *E. faecalis* [20, 31]. Given that PPIases are present in most types of cells, and that they have an essential role in the folding of proteins, hence acting on bacterial growth and resistance to β-lactam [21–23], we hypothesized that polyclonal antibodies raised against PpiC might be cross-reactive against *S. aureus*.

We show in this study that, after rabbit immunization with PpiC, the serum contains cross-opsonic antibodies capable of mediating killing of a wide variety of *S. aureus* strains, beside *E. faecium* and *E. faecalis* strains. To follow up on these results, we bioinformatically identified and biophysically characterised PrsA, the PpiC homologous protein in *S. aureus*, most likely responsible for the observed cross-reactivity. We demonstrated the great potential of PrsA as a vaccine antigen by using a variety of immunological assays. Significant increases in PrsA-specific antibodies after immunization of rabbits was observed, indicating that the PPIase is indeed immunogenic. We also showed that anti-PpiC serum binds to PrsA and inversely, anti-PrsA binds to the enterococcal PPIase PpiC, proving the implication of both proteins in the cross-reactivity studied and suggesting the presence of shared epitopes. By using the opsonophagocytic assay, we demonstrated that anti-PrsA, like anti-PpiC, contain cross-opsonic antibodies able to mediate killing of a wide variety of *S. aureus*, as well as *E. faecium* and *E. faecalis* strains. Despite the low sequence identity between the two proteins (about 24%), we observed specific regions with high sequence conservation and high antigenicity prediction scores. We may surmise that these regions may act as epitopes against both *S. aureus* and enterococci; however, experimental assessment is needed to validate this hypothesis.

The development of vaccines presents many challenges. Some important aspects are the time and expenses needed to obtain novel formulations through long and costly clinical trials. In this study, we present an antigen that shows promising results for immunization against more than one pathogen. We also describe an innovative approach to identify antigens that could work against several target, which we believe is a smart way to tackle these challenges [43].

Besides being responsible for catalyzing the post-translocational folding of exported proteins [22, 44, 45], the membrane-anchored foldase protein PrsA also plays an important role in physiology and pathogenicity of many pathogenic bacteria such as streptococci, *Helicobacter pylori* and *Listeria monocytogenes* [44–46]. PrsA has also been shown to be involved in virulence of streptococci [47]. Penicillin-binding proteins are found to be unstable in PrsA null mutant, supporting the idea that PrsA is required for the folding of penicillin-binding proteins and therefore essential to the bacterial growth [22]. Consistent with our results, PrsA was also predicted as a potential vaccine target in MRSA by reverse vaccinology and subtractive genomics approaches [48].

The data from our study, together with the current information found in the literature, suggests that PPIases should be further investigated as pan-vaccinomics targets. Indeed, these ubiquitous enzymes were found to be interesting vaccine targets in several organisms. PrsA was identified by reverse vaccinology in *Streptococcus sanguinis*, a pathogen responsible for infective endocarditis [49]. Additionally, bacterial Macrophage Infectivity Potentiator (MIP)-like PPIases are known to be candidates in vaccines protective against both animals and humans. *Neisseria gonorrhoeae* and *Neisseria meningitidis*, respectively responsible for meningitis and sepsis, or the sexually transmitted disease gonorrhea, both express a MIP-PPIase which appear to play a role in intracellular life of the pathogens, helping escape the immune system. Furthermore, patients infected with *N. gonorrhoeae* contained MIP-PPIase-specific antibodies in their blood, suggesting its antigenicity and potential use as a vaccine target. The review by Humbert et al. lists several bacterial strains where MIP-like PPIase were successfully studied as vaccine antigens [50]. Given the ubiquitous characteristic of PPIase, concerns regarding the potential targeting of commensal bacteria can arise. However, vaccines already in clinical use showed no indication of specific antibodies affecting the microflora. The non-exhaustive list of pathogens having PPIases identified as vaccine targets support the idea those enzymes should be further investigated in the frame of pan-vaccinomics. Such approaches are a great hope in the ongoing fight against antimicrobial resistance and for control of infections caused by pathogens listed by the WHO [51].

## Conclusions

While the antibiotic resistance threat keeps rising, great hope can be placed in the development of vaccines, especially in the case of the two dangerous Gram-positive ESKAPE bacteria *S. aureus* and enterococci. Identification of potent antigens is the key to successfully develop preventive treatments. Our study shows that the evaluation of already discovered antigens that have essential roles within a bacterial cell should be conducted, and can reveal a cross-reactive effect. Investigation of this effect led us to the discovery a new antigen, PrsA, for which we demonstrate the potential as an antigen to use in vaccine formulation against the two nosocomial pathogens. Our findings support the idea that pan-vaccinomics is reachable and that PPIases should be investigated as potential effective antigens.

### Electronic supplementary material

Below is the link to the electronic supplementary material.

Supplementary Material 1

Supplementary Material 2

## Data Availability

Data and materials described in this article will be made available on request.

## Abbreviations

BHI: Brain Heart Infusion
BSI: Bloodstream Infections
CD: Circular Dichroism
CDC: Center for Disease Control and Prevention
E. faecalis: Enterococcus faecalis
E. faecium: Enterococcus faecium
ELISA: Enzyme-Linked ImmunoSorbent Assay
HAIs: Hospital Acquired Infections
IgG: Immunoglobulin G
LDDT: Local Distance Difference Test
MIP: Macrophage Infectivity Potentiator
MRSA: Methicillin-Resistant S. aureus
OPA: Opsonophagocytic Assay
PBS-BSA: Phosphate Buffer Saline with 3% of Bovine Serum Albumin
PPIase: Peptidyl-Prolyl cis/trans Isomerase
RPMIF: Roswell Park Memorial Institute 1640 medium with 15% fetal bovine serum
S. aureus: Staphylococcus aureus
SEC-MALS: Size Exclusion Chromatography-Multi Angle Light Scattering
SEM: Standard Error of the Mean
SSI: Surgical Site Infections
VRE: Vancomycin-Resistant Enterococci
US: United States
WHO: World Health Organization
