# Supplementary material for: Investigation of cross-opsonic effect leads to the discovery of PPIase-domain containing protein vaccine candidate to prevent infections by Gram-positive ESKAPE pathogens
Source: BMC Microbiol. 2024 Jul 27;24:280. doi: 10.1186/s12866-024-03427-w (PMC11282748; doi:10.1186/s12866-024-03427-w)
Supplement: Supplementary file 1 — Supplementary Material 1 [file 12866_2024_3427_MOESM1_ESM.docx]

Additional file 1

Supporting information

Investigation of cross-opsonic effect leads to the discovery of PPIase-domain containing protein vaccine candidate to prevent infections by Gram-positive ESKAPE pathogens.

Océane Sadones^1^, Eliza Kramarska^2^, Diana Laverde^1^, Rita Berisio^2^, Johannes Huebner^1*^, and Felipe Romero-Saavedra^1^

1. Division of pediatric infectious disease, Hauner children’s hospital, LMU, Munich, Germany.
2. Institute of Biostructures and Bioimaging, Italian Research Council (CNR), Naples, Italy.

*Correspondence: [johannes.huebner@med.uni-muenchen.de](mailto:johannes.huebner@med.uni-muenchen.de)

Table S1: Bacterial strains used for this study.

Table S2: Primers used for this study.

Table S3: Sera used for this study.

# Bacterial strains

Table S1: Bacterial strains used for this study.

| Strains | Description | Reference |
| --- | --- | --- |
| *Staphylococcus aureus* |  |  |
| *S. aureus* MW2 | Community-acquired MRSA isolate from the US (North Dakota) | [1] |
| *S. aureus* LAC | Community-acquired MRSA isolate from the US (Los Angeles) | [2] |
| *S. aureus* Reynolds | Clinical isolate | [3] |
| *S. aureus* MN8 | Clinical isolate | [4] |
| *S. aureus* 194 | Isolate from food environment | [5] |
| *S. aureus* SF8300 | Community-acquired MRSA isolate from the US (San Francisco) | [2] |
| Enterococci |  |  |
| *E. faecium* VRE11236/1 | Vancomycin-resistant isolated from a patient in Germany (Munich) | [6] |
| *E. faecalis* 12030 | Isolated from a patient in the US (Cleaveland) | [7] |
| *E. faecalis* Type 2 | Isolated from a patient in Japan (Sapporo) | [8] |
| *Escherichia coli* |  |  |
| *E. coli* M15/pQE30PpiC | M15 harboring pRep4 and pQE30PpiC plasmids | [9] |
| *E. coli* M15/pQE30PrsA | M15 harboring pRep4 and pQE30PrsA plasmids | This study |

**Production of recombinant proteins**

Table S2: Primers used for this study.

| Name | 5’-3’ sequence* | Restriction site |
| --- | --- | --- |
| PrsA_BamHI_Fw | **GACTCAggatcc**GCTTGTGGCGCTAGTGCC | BamHI |
| PrsA_KpnI_Rv | **GACTCAggtacc**TTATTGGCTCATGCCGGATTG | KpnI |

*Bases in orange lowercase letters correspond to the restriction site. Underlined green bases are not complementary to the target sequence.

**Rabbit immunization**

Table S3: Sera used for this study.

| Sera | Description | Reference |
| --- | --- | --- |
| Pre-PpiC | Pre-immune serum collected from rabbits immunized with PpiC | [9] |
| Anti-PpiC | Anti-protein serum collected from rabbits immunized with PpiC | [9] |
| Pre-PrsA | Pre-immune serum collected at day 0 from rabbits immunized with PrsA | This study |
| Anti-PrsA | Anti-protein serum collected at day 49 from rabbits immunized with PrsA | This study |

## References

[1] CDC, “Four pediatric deaths from community-acquired methicillin-resistant Staphylococcus aureus — Minnesota and North Dakota, 1997-1999,” *MMWR Morb Mortal Wkly Rep*, vol. 48, no. 32, pp. 707–710, Aug. 1999.

[2] B. A. Diep *et al.*, “Contribution of Panton-Valentine Leukocidin in Community-Associated Methicillin-Resistant Staphylococcus aureus Pathogenesis,” *PLoS ONE*, vol. 3, no. 9, p. e3198, Sep. 2008, doi: 10.1371/journal.pone.0003198.

[3] D. McKenney, J. Hübner, E. Muller, Y. Wang, D. A. Goldmann, and G. B. Pier, “The ica locus of Staphylococcus epidermidis encodes production of the capsular polysaccharide/adhesin,” *Infect Immun*, vol. 66, no. 10, pp. 4711–4720, Oct. 1998, doi: 10.1128/IAI.66.10.4711-4720.1998.

[4] B. N. Kreiswirth *et al.*, “The toxic shock syndrome exotoxin structural gene is not detectably transmitted by a prophage,” *Nature*, vol. 305, no. 5936, pp. 709–712, Oct. 1983, doi: 10.1038/305709a0.

[5] P. Di Ciccio *et al.*, “Biofilm formation by Staphylococcus aureus on food contact surfaces: Relationship with temperature and cell surface hydrophobicity,” *Food Control*, vol. 50, pp. 930–936, Apr. 2015, doi: 10.1016/j.foodcont.2014.10.048.

[6] F. Romero-Saavedra *et al.*, “Conjugation of Different Immunogenic Enterococcal Vaccine Target Antigens Leads to Extended Strain Coverage,” *The Journal of Infectious Diseases*, vol. 220, no. 10, pp. 1589–1598, Oct. 2019, doi: 10.1093/infdis/jiz357.

[7] J. Huebner *et al.*, “Isolation and chemical characterization of a capsular polysaccharide antigen shared by clinical isolates of Enterococcus faecalis and vancomycin-resistant Enterococcus faecium,” *Infect Immun*, vol. 67, no. 3, pp. 1213–1219, Mar. 1999, doi: 10.1128/IAI.67.3.1213-1219.1999.

[8] S. Maekawa, M. Yoshioka, and Y. Kumamoto, “Proposal of a New Scheme for the Serological Typing of *Enterococcus faecalis* Strains,” *Microbiology and Immunology*, vol. 36, no. 7, pp. 671–681, Jul. 1992, doi: 10.1111/j.1348-0421.1992.tb02070.x.

[9] F. Romero-Saavedra *et al.*, “Identification of Peptidoglycan-Associated Proteins as Vaccine Candidates for Enterococcal Infections,” *PLoS ONE*, vol. 9, no. 11, p. e111880, Nov. 2014, doi: 10.1371/journal.pone.0111880.
