# Supplementary material for: Investigation of cross-opsonic effect leads to the discovery of PPIase-domain containing protein vaccine candidate to prevent infections by Gram-positive ESKAPE pathogens
Source: BMC Microbiol. 2024 Jul 27;24:280. doi: 10.1186/s12866-024-03427-w (PMC11282748; doi:10.1186/s12866-024-03427-w)
Supplement: Supplementary file 2 — Supplementary Material 2 [file 12866_2024_3427_MOESM2_ESM.docx]

Additional file 2

Supporting information

Investigation of cross-opsonic effect leads to the discovery of PPIase-domain containing protein vaccine candidate to prevent infections by Gram-positive ESKAPE pathogens.

Océane Sadones^1^, Eliza Kramarska^2^, Diana Laverde^1^, Rita Berisio^2^, Johannes Huebner^1*^, and Felipe Romero-Saavedra^1^

1. Division of pediatric infectious disease, Hauner children’s hospital, LMU, Munich, Germany.
2. Institute of Biostructures and Bioimaging, Italian Research Council (CNR), Naples, Italy.

*Correspondence: [johannes.huebner@med.uni-muenchen.de](mailto:johannes.huebner@med.uni-muenchen.de)

Figure S1: Visual representation of alignment of *S. aureus* AdcA sequences

Figure S2: The dataset of *S. aureus* PrsA used for alignment

**Figure S1: Visual representation of alignment of *S. aureus* AdcA sequences using JalView programme after Clustal Omega processing.**

Yellow bars represent conservation of amino acids based on the JalView conservation annotation, black bars present residue consensus with the most present amino acid present on the bars.

**
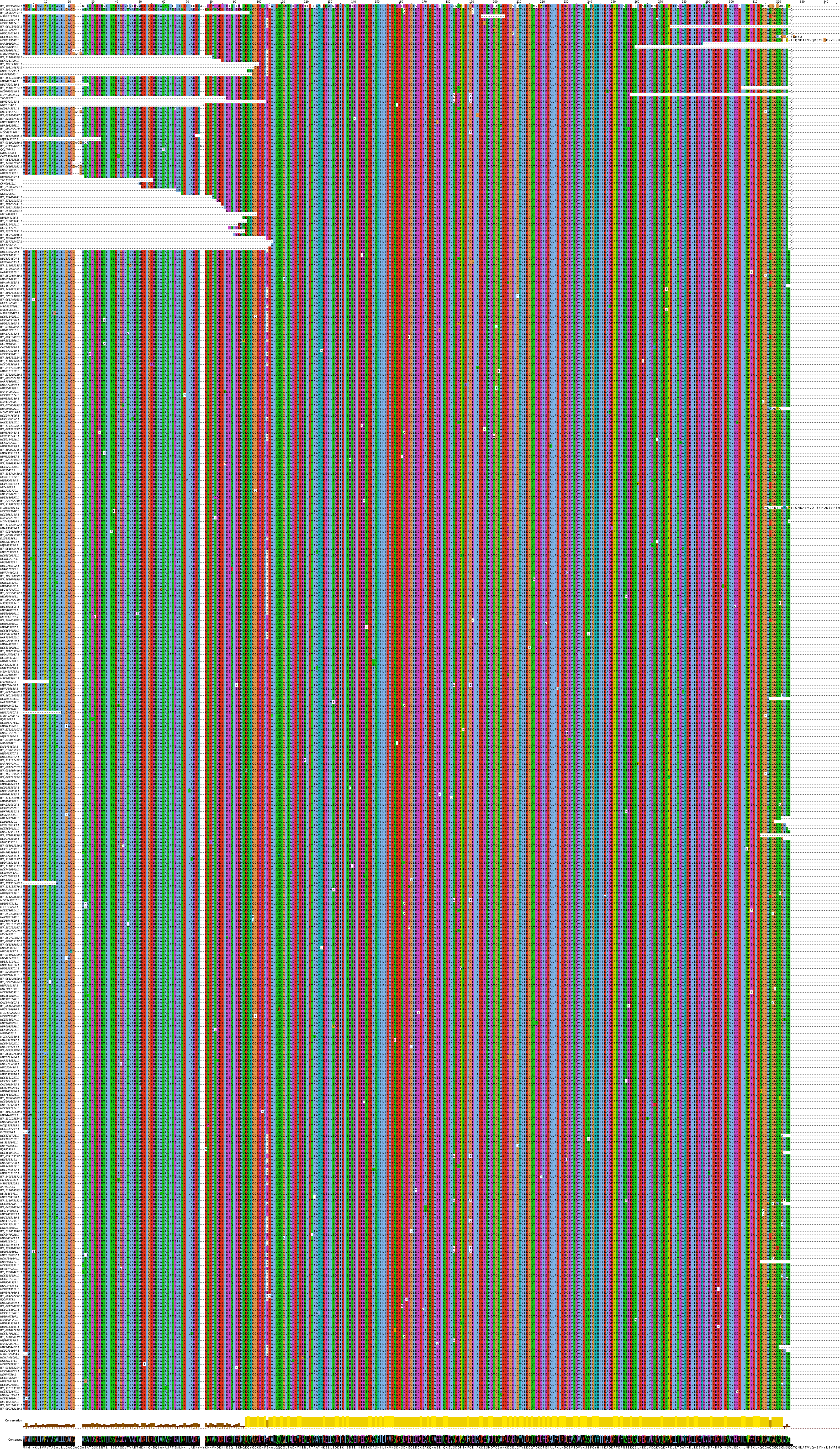
**

**Figure S2: The dataset of *S. aureus* PrsA used for alignment obtained from BlastP database with 50% sequence coverage**

>HDC5213484.1 peptidylprolyl isomerase [Staphylococcus aureus]

MKMINKLIVPVTASALLLGACGASATDSKENTLISSKAGDVTVADTMKKIGKDQIANASFTEMLNKILADKYKNKVNDKKIDEQIEKMQKQYGGKDKFEKALQQQGLTADKYKENLRTAAYHKELLSDKIKISDSEIKEDSKKASHILIKVKSKKSDKEGLDDKEAKQKAEEIQKEVSKDPSKFGEIAKKESMDTGSAKKGGELGYVLKGQTDKDFEKALFKLKDGEVSDVVKSSFGYHIIKADKPTDFNSEKQSLKEKLVDQKVQKNPKLLTDAYKDLLKEYDVDFKDRDIKSVVEDKILNPEKLKQGGAQGGQSGMSQ

>WP_115399457.1 foldase protein PrsA [Staphylococcus aureus]

MKMINKLIVPVTASALLLGACGASATDSKENTLISSKAGDVTVADTMKKIGKDQIANASFTEMLNKILADKYKNKVNDKKIDEQIEKMQKQYGGKDKFEKALQQQGLTADKYKENLRTAAYHKELLSDKIKISDSEIKEDSKKASHILIKVKSKKSDKEGLDDKEAKQKAEEIQKEVSKDPSKFGEIAKKESMDTGSAKKGGELGYVLKGQTDKDFEKALFKLKDGEVSDVVKSSFGYHIIKADKPTDFNSEKQSLKEKLVDQKVQKNPKLLTDAYKDLLKEYDVDFKDRDIKSVVEDKILNPEKLKQGGAKGGQSGMSQ

>HDC5186827.1 peptidylprolyl isomerase [Staphylococcus aureus]

MKMINKLIVPVTASALLLGACGACATDSKENTLISSKAGDVTVADTMKKIGKDQIANASFTEMLNKILADKYKNKVNDKKIDEQIEKMQKQYGGKDKFKKALQQQGLTADKYKENLRTAAYHKELLSDKIKISDSEIKEDSKKASHILIKVKSKKSDKEGLDDKEAKQKAEEIQKEVSKDPSKFGEIAKKESMDTGSAKKDGELGYVLKGQTDKDFEKALFKLKDGEVSDVVKSSFGYHIIKADKPTDFNSEKQSLKEKLVDQKVQKNPKLLTDAYKDLLKEYDVDFKDRDIKSVVEDKILNPEKLKQGGAQGGQSGMSQ

>WP_111078152.1 foldase protein PrsA [Staphylococcus aureus]

MKMINKLIVPVTASALLLGACGASATDSKENTLISSKAGDVTVADTMKKIGKDQIANASFTEMLNKILADKYKNKVNDKKIDEQIEKMQKQYGGKDKFEKALQQQGLTADKYKENLRTAAYHKELLSDKIKISDSEIKEDSKKASHILIKVKSKKSDKEGLDDKEAKQKAEEIQKEVAKDPSKFAEIAKKESMDTGSAKKDGELGYVLKGQTDKDFEKALFKLKDGEVSDVVKSSFGYHIIKADKPTDFNREKQSLKEKLVDQKVQKNPKLLTDAYKDLLKEYDVDFKDRDIKSVVEDKILNPEKLKQGGAQGGQSGRSQ

>WP_262607580.1 foldase protein PrsA [Staphylococcus aureus]

MKMINKLIVLVTASALLLGACGASATDSKENTLISSKAGDVTVADTMKKIGKDQIANASFTEMLNKILADKYKNKVNDKKIDEQIEKMQKQYGGKDKFEKALQQQGLTADKYKENLRTAAYHKELLSDKIKISDSEIKEDSKKASHILIKVKSKKSDKEGLDDKEAKQKAEEIQKEVSKDPSKFGEIAKKESMDTGSAKKDGELGYVLKGQTDKDFEKALFKLKDGEVSDVVKSSFGYHIIKADKPTDFNSEKQSLKEKLVDQKVQKNPKLLTDAYKDLLKEYDVDFKDRDIKSVVEDKILNPEKLKQGGAQGGQSGMSQ

>WP_160186291.1 foldase protein PrsA [Staphylococcus aureus]

MKMINKLIVPVTASALLLGACGASATDSKENTLISSKAGDVTVADTMKKIGKDQIANASFTEMLNKIFADKYKNKVNDKKIDEQIEKMQKQYGGKDKFEKALQQQGLTADKYKENLRTAAYHKELLSDKIKISDSEIKEDSKKASHILIKVKSKKSDKEGLDDKEAKQKAEEIQKEVSKDPSKFGEIAKKESMDTGSAKKDGELGYVLKGQTDKDFEKALFKLKDGEVSDVVKSSFGYHIIKADKPTDFNSEKQSLKEKLVDQKVQKNPKLLTDAYKDLLKEYDVDFKDRDIKSVVEDKILNPEKLKQGGAQGGQSGMSQ

>MCM0579148.1 peptidylprolyl isomerase [Staphylococcus aureus]

MKMINKLIVPVTASALLLGACGASATDSKENTLISSKAGDVTVADTMKKIGKDQIANASFTEMLNKILADKYKNKVNDKKIDEQIEKMQKQYGGKDKFEKALQQQGLTADKYKENLRTAAYHKELLSDKIKISDSEIKEDSKKASHILIKVKSKKSDKEGLDDKEAKQKAEEIQKEVSKDPSKFGEIAKKESMDTGSAKKDGELGYVLKGQTDKDFEKALFKLKDGEVSDVVKSSFGYHIIKANKPTDFNSEKQSLKEKLVDQKVQKNPKLLTDAYKDLLKEYDVDFKDRDIKSVVEDKILNPEKLKQGGAQGGQSGMSQ

>HCG2587764.1 peptidylprolyl isomerase [Staphylococcus aureus]

MKMINKLIVPVTASALLLGACGASATDSKENTLISSKAGDVTVADTMKKIGKDQIANASFTEMLNKILANKYKNKVNDKKIDEQIEKMQKQYGGKDKFEKALQQQGLTADKYKENLRTAAYHKELLSDKIKISDSEIKEDSKKASHILIKVKSKKSDKEGLDDKEAKQKAEEIQKEVSKDPSKFGEIAKKESMDTGSAKKDGELGYVLKGQTDKDFEKALFKLKDGEVSDVVKSSFGYHIIKADKPTDFNSEKQSLKEKLVDQKVQKNPKLLTDAYKDLLKEYDVDFKDRDIKSVVEDKILNPEKLKQGGAQGGQSGMSQ

>WP_089535766.1 foldase protein PrsA [Staphylococcus aureus]

MKMINKLIVPVTASALLLGACGASATDSKENTLISSKAGDVTVADTMKKIGKDQIANASFTEMLNKILADKYKNKVNDKKIDEQIEKMQKQYGGKDKFEKALQQQGLTADKYKENLRTAAYHKELLSDKIKISDSEIKEDSKKASHILIKVKSKKSDKEGLDDKEAKQKAEEIQKEVSKDPSKFGEIAKKESMDTGSAKKDGELGYVLKGQTDKDFEKALFKLKDGEVSDVVKSSFGYHIIKADKPTDFNSEKQSLKEKLVDQKVQKNPKLLTDAYKDLLKEYNVDFKDRDIKSVVEDKILNPEKLKQGGAQGGQSGMSQ

>HDC3991213.1 peptidylprolyl isomerase [Staphylococcus aureus]

MKMINKLIVPVTASALLLGACGASATDSKENTLISSKAGDVTVADTMKKIGKDQIANASFTEMLNKILADKYKNKVNDKKIDEQIEKMQKQYGGKDKFEKALQQQGLTADKYKENLRTAAYHKELLSDKIKISDSEIKEDSKKASHILIKVKSKKSDKEDLDDKEAKQKAEEIQKEVSKDPSKFGEIAKKESMDTGSAKKDGELGYVLKGQTDKDFEKALFKLKDGEVSDVVKSSFGYHIIKADKPTDFNSEKQSLKEKLVDQKVQKNPKLLTDAYKDLLKEYDVDFKDRDIKSVVEDKILNPEKLKQGGAQGGQSGMSQ

>HDH6780483.1 peptidylprolyl isomerase [Staphylococcus aureus]

MKMINKLIVPVTASALLLGACGASATDSKKNTLISSKAGDVTVADTMKKIGKDQIANASFTEMLNKILADKYKNKVNDKKIDEQIEKMQKQYGGKDKFEKALQQQGLTADKYKENLRTAAYHKELLSDKIKISDSEIKEDSKKASHILIKVKSKKSAKEGLDDKEAKQKAEEIQKEVSKDPSKFGEIAKKESMDTGSAKKDGELGYVLKGQTDKDFEKALFKLKDGEVSDVVKSSFGYHIIKADKPTDFNSEKQSLKEKLVDQKVQKNPKLLTDAYKDLLKEYDVDFKDRDIKSVVEDKILNPEKLKQGGAQGGQSGMSQ

>WP_109162114.1 foldase protein PrsA [Staphylococcus aureus]

MKMINKLIVPVTASALLLGACGASATDSKENTLISSKAGDVTVADTMKKIGKDQIANASFTEMLNKILADKYKNKVNDKKIDEQIEKMQKQYGGKDKFEKALQQQGLTADKYKENLRTAAYHKELLSDKIKISDSEIKEDSKKASHILIKVKSKKSAKEGLDDKEAKQKAEEIQKEVSKDPSKFGKIAKKESMDTGSAKKDGELGYVLKGQTDKDFEKALFKLKDGEVSDVVKSSFGYHIIKADKPTDFNSEKQSLKEKLVDQKVQKNPKLLTDAYKDLLKEYDVDFKDRDIKSVVEDKILNPEKLKQGGAQGGQSGMSQ

>HDH6201917.1 peptidylprolyl isomerase [Staphylococcus aureus LTCF-15-62]

MKMINKLIVPVTASALLLGACGASATDSKENTLISSKAGDVTVADTMKKIGKDQIANASFTEMLNKILADKYKNKVNDKKIDEQIEKMQKQYGGKDKFEKALQQQGLTADKYKENLRTAAYHKELLSDKIKISDSEIKEDSKKASHILIKVKSKKIDKEGLDDKEAKQKAEEIQKEVSKDPSKFGEIAKKESMDTGSAKKDGELGYVLKGQTDKDFEKALFKLKDGEVSDVVKSSFGYHIIKADKPTDFNSEKQSLKEKLVDQKVQKNPKLLTDAYKDLLKEYDVDFKDRDIKSVVEDKILNPEKLKQGGAQGGQSGMSQ

>WP_061740013.1 foldase protein PrsA [Staphylococcus aureus]

MKMIKKLIVPVTASALLLGACGASATDSKENTLISSKAGDVTVADTMKKIGKDQIANASFTEMLNKILADKYKNKVNDKKIDEQIEKMQKQYGGKDKFEKALQQQGLTADKYKENLRTAAYHKELLSDKIKISDSEIKEDSKKASHILIKVKSKKSDKEGLDDKEAKQKAEEIQKEVSKDPSKFGEIAKKESMDTGSAKKDGELGYVLKGQTDKDFEKALFKLKDGEVSDVVKSSFGYHIIKADKPTDFNSEKQSLKEKLVDQKVQKNPKLLTDAYKDLLKEYDVDFKDRDIKSVVEDKILNPEKLKQGGAQGGQSGMSQ

>WP_303751324.1 foldase protein PrsA [Staphylococcus aureus]

MKMINKLIVPVTASALLLGACGASATDSKENTLISSKAGDVTVADTMKKIGKDQIANASFTEMLNKILADKYKNKVNDKKIDEQIEKMQKQYGGKDKFEKALQQQGLTADKYKENLRTAAYHKELLSDKIKISDSEIKEDSKKASHILIKVKSKKSDKEGLDDKEAKQKAEEIQKEVSKDPSKFGEIAKKESMDTGSAKKDGELGYVLKGQTDKEFEKALFKLKDGEVSDVVKSSFGYHIIKADKPTDFNSEKQSLKEKLVDQKVQKNPKLLTDAYKDLLKEYDVDFKDRDIKSVVEDKILNPEKLKQGGAQGGQSGMSQ

>HCQ2233395.1 peptidylprolyl isomerase [Staphylococcus aureus]

MKMINKLIVPVTASALLLGACGASATDSKENTLISSKAGDVTVADTMKKIGKDQIANASFTEMLNKILADKYKDKVNDKKIDEQIEKMQKQYGGKDKFEKALQQQGLTADKYKENLRTAAYHKELLSDKIKISDSEIKEDSKKASHILIKVKSKKSDKEGLDDKEAKQKAEEIQKEVSKDPSKFGEIAKKESMDTGSAKKDGELGYVLKGQTDKDFEKALFKLKDGEVSDVVKSSFGYHIIKADKPTDFNSEKQSLKEKLVDQKVQKNPKLLTDAYKDLLKEYDVDFKDRDIKSVVEDKILNPEKLKQGGAQGGQSGMSQ

>HAR5297370.1 peptidylprolyl isomerase [Staphylococcus aureus]

MKMINKLIVPVTASALLLGACGASATDSKENTLISSKAGDVTVADTMKKIGKDQIANASFTEMLNKILADKYKNKVTDKKIDEQIEKMQKQYGGKDKFEKALQQQGLTADKYKENLRTAAYHKELLSDKIKISDSEIKEDSKKASHILIKVKSKKSDKEGLDDKEAKQKAEEIQKEVSKDPSKFGEIAKKESMDTGSAKKDGELGYVLKGQTDKDFEKALFKLKDGEVSDVVKSSFGYHIIKADKPTDFNSEKQSLKEKLVDQKVQKNPKLLTDAYKDLLKEYDVDFKDRDIKSVVEDKILNPEKLKQGGAQGGQSGMSQ

>HDE3973356.1 peptidylprolyl isomerase [Staphylococcus aureus]

MKMINKLIVPATASALLLGACGASATDSKENTLISSKAGDVTVADTMKKIGKDQIANASFTEMLNKILADKYKNKVNDKKIDEQIEKMQKQYGGKDKFEKALQQQGLTADKYKENLRTAAYHKELLSDKIKISDSEIKEDSKKASHILIKVKSKKSDKEGLDDKEAKQKAEEIQKEVSKDPSKFGEIAKKESMDTGSAKKDGELGYVLKGQTDKDFEKALFKLKDGEVSDVVKSSFGYHIIKADKPTDFNSEKQSLKEKLVDQKVQKNPKLLTDAYKDLLKEYDVDFKDRDIKSVVEDKILNPEKLKQGGAQGGQSGMSQ

>HCX1102088.1 peptidylprolyl isomerase [Staphylococcus aureus]

MKMINKLIVPVTASALLLGACGASATDSKENTLISSKAGDVTVADTMKKIGKDQIANASFTEMLNKILADKYKNKVNDKKIDEQIEKMQKQYGGKDKFEKALQQQGLTADKYKENLRTAAYHKELLSDKIKISDSEIKEDSKKASHILIKVKSKKSDKEGLDDKEAKQKAEEIQKEVSKDPSKFGEIAKKESMDTGSAKKDGELGYVLKGQTDKDFEKALFKLKDGEVSDVVKSSFGYHIIKADKPTDFNSEKQSLKEKLVDQKVQKNPKLLTDAYKDLLKDYDVDFKDRDIKSVVEDKILNPEKLKQGGAQGGQSGMSQ

>ELL5582965.1 peptidylprolyl isomerase [Staphylococcus aureus]

MKMINKLIVPVTASALLLGACGASATDSKENTLISSKAGDVTVADTMKKIGKDQIANASFTEMLNKILADKYKNKVNDKKIDEQIEKMQKQYGGKDKFKKALQQQGLTADKYKENLRTAAYHKELLSDKIKISDSEIKEDSKKASHILIKVKSKKSDKEGLDDKEAKQKAEEIQKEVSKDPSKFGEIAKKESMDTGSAKKGGELGYVLKGQTDKDFEKALFKLKDGEVSDVVKSSFGYHIIKADKPTDFNSEKQSLKEKLVDQKVQKNPKLLTDAYKDLLKEYDVDFKDRDIKSVVEDKILNPEKLKQGGAQGGQSGMSQ

>HDL0580191.1 peptidylprolyl isomerase [Staphylococcus aureus]

MKMISKLIVPVTASALLLGACGASATDSKENTLISSKAGDVTVADTMKKIGKDQIANASFTEMLNKILADKYKNKVNDKKIDEQIEKMQKQYGGKDKFEKALQQQGLTADKYKENLRTAAYHKELLSDKIKISDSEIKEDSKKASHILIKVKSKKSDKEGLDDKEAKQKAEEIQKEVAKDPSKFAEIAKKESMDTGSAKKDGELGYVLKGQTDKDFEKALFKLKDGEVSDVVKSSFGYHIIKADKPTDFNSEKQSLKEKLVDQKVQKNPKLLTDAYKDLLKEYDVDFKDRDIKSVVEDKILNPEKLKQGGAQGGQSGMSQ

>HDE0589380.1 peptidylprolyl isomerase [Staphylococcus aureus]

MKMINKLIVPVTASALLLGACGASATDSKENTLISSKAGDVTVADTMKKIGKDQIANASFTEMLNKILADKYKNKVNDKKIDEQIEKMQKQYGGKDKFEKALQQQGLTADKYKENLRTAAYHKELLSDKIKISDSEIKEDSKKASHILIKVKSKKSDKEGLDDKEAKQKAEEIQKEVSKDPSKFGEIAKKESMDTGSAKKDGELGYVLKGQTDKDFKKALFKLKDGEVSDVVKSSFGYHIIKADKPTDFNSEKQSLKEKLVDQKVQKNPKLLTDAYKDLLKEYDVDFKDRDIKSVVEDKILNPEKLKQGGAQGGQSGMSQ

>HDD0310254.1 peptidylprolyl isomerase [Staphylococcus aureus]

MKMINKLIVPVTASALLLGACGASATDSKENTLISSKAGDVTVADTMKKIGKDQIANASFTEMLNKILADKYKNKVNDKKIDEQIEKMQKQYGGKDKFEKALQQQGLTADKYKENLRTAAYHKELLSDKIKISDSEIKEDSKKASHILIKVKSKKSDKEGLDDKEAKQKAEEIQKEVSKDPSKFGEIAKKESMDTGSAKKDGKLGYVLKGQTDKDFEKALFKLKDGEVSDVVKSSFGYHIIKADKPTDFNSEKQSLKEKLVDQKVQKNPKLLTDAYKDLLKEYDVDFKDRDIKSVVEDKILNPEKLKQGGAQGGQSGMSQ

>WP_061391637.1 foldase protein PrsA [Staphylococcus aureus]

MKMINKLIVPVTASALLLGACGASATDSKENTLISSKAGDVTVADTMKKIGKDQIANASFTEMLNKILADKYKNKVNDKKIDEQIEKMQKQYGGKDKFEKALQQQGLTADKYKENLRTAAYHKELLSDKIKISDSEIKEDSKKASHILIKVKSKKSDKEGLDDKEAKQKAEEIQKEVSKDPSKFGEIAKKKSMDTGSAKKDGELGYVLKGQTDKDFEKALFKLKDGEVSDVVKSSFGYHIIKADKPTDFNSEKQSLKEKLVDQKVQKNPKLLTDAYKDLLKEYDVDFKDRDIKSVVEDKILNPEKLKQGGAQGGQSGMSQ

>HAR3700776.1 peptidylprolyl isomerase [Staphylococcus aureus]

MKMINKLIVPVTASALLLGACGASATDSKENTLISSKAGDVTVADTMKKIGKDQIANASFTEMLNKILADKYKNKVNDKKIDEQIEKMQKQYGGKDKFEKALQQQGLTADKYKENLRTAAYHKELLSDKIKISDSEIKEDSKKASHILIKVKSKKSDKEGLDDKEAKQKAKEIQKEVSKDPSKFGEIAKKESMDTGSAKKDGELGYVLKGQTDKDFEKALFKLKDGEVSDVVKSSFGYHIIKADKPTDFNSEKQSLKEKLVDQKVQKNPKLLTDAYKDLLKEYDVDFKDRDIKSVVEDKILNPEKLKQGGAQGGQSGMSQ

>WP_054189557.1 foldase protein PrsA [Staphylococcus aureus]

MKMINKLIVPVTASALLLGACGASATDSKENTLISSKAGDVTVADTMKKIGKDQIANASFTEMLNKILADKYKNKVNDKKIDEQIEKMQKQYGGKDKFEKALQQQGLTADKYKENLRTAAYHKELLSDKIKISDSEIKEDSKKASHILIKVKSKKSDKEGLDDKEAKQKAEEIQKEVAKDPSKFAEIAKKESMDTGSAKKDGELGYVLKGQTDKDFEKALFKLKDGEVSDVVKSSFGYHIIKADKPTDFNSEKQSLKEKLVDQKVQKNPKLLTDAYKDLLKEYDVDFKDRDIKSVVEDKILNPEKLKQGGAQGGQSGMSQ

>MDG5456010.1 peptidylprolyl isomerase [Staphylococcus aureus]

MKMINKLIVPVTASALLLGACGASATDSKENTLISSKAGDVTVADTMKKIGKDQIANASFTEMLNKILADKYKNKVNDKKIDEQIEKMQKQYGGKDKFEKALQQQGLTADKYKENLRTAAYHKELLSDKIKISDSEIKEDSKKASHILIKVKSKKSDKEGLDDKEAKQKAEEIQKEVAKDPSKFAEIAKKESMDTGSAKKDGELGYVLKGQTDKDFEKALFKLKDGEVSDVVKSSFGYHIIKADKPTDFNSEKQSLKEKLVDQKVQKNPKLLTDAYKDLLKEYDVDFKDRDIKSVVEDKILNPEKLKQGGAQGGQSGISQ

>HCY6498027.1 peptidylprolyl isomerase [Staphylococcus aureus]

MKMINKLIVPVTASALLLGACGASATDSKENTLISSKAGDVTVADTMKKIGKDQIANASFTEMLNKILADKYKNKVNDKKIDEQIEKMQKQYGGKDKFEKALQQQGLTADKYKENLRTAAYHKELLSDKIKISDSEIKEDSKKASHILIKVKSKKSDKEGLDDKEAKQKAEEIQKEASKDPSKFGEIAKKESMDTGSAKKDGELGYVLKGQTDKDFEKALFKLKDGEVSDVVKSSFGYHIIKADKPTDFNSEKQSLKEKLVDQKVQKNPKLLTDAYKDLLKEYDVDFKDRDIKSVVEDKILNPEKLKQGGAQGGQSGMSQ

>HDJ3073370.1 peptidylprolyl isomerase [Staphylococcus aureus]

MKMINKLIVPVTASALLLGACGASATDSKENTLISSKAGDVTVADTMKKIGKDQIANASFTEMLNKILADKYKNKVNDKKIDEQIEKMQKQYGGKDKFEKALQQQGLTADKYKENLRTAAYHKELLSDKIKISDSEIKEDSKKASHILIKVKSKKSDKEGLDDKEAKQKAEEIQKEVAKDPSKFGEIAKKESMDTGSAKKDGELGYVLKGQTDKDFEKALFKLKDGEVSDVVKSSFGYHIIKADKPTDFNSEKQSLKEKLVDQKVQKNPKLLTDAYKDLLKEYDVDFKDRDIKSVVEDKILNPEKLKQGGAQGGQSGMSQ

>HDG8718089.1 peptidylprolyl isomerase [Staphylococcus aureus]

MKMINKLIVPVTASALLLGACGASATDSKENTLISSKAGDVTVADTMKKIGKDQIANASFTEMLNKILADKYKNKVNDKKIDEQIEKMQKQYGGKDKFEKALQQQGLTADKYKENLRTAAYHKELLSDKIKISDSEIKEDSKKASHILIKVKSKKSDKEGLDDKEAKQKAEEIQKEVSKDPSEFGEIAKKESMDTGSAKKDGELGYVLKGQTDKDFEKALFKLKDGEVSDVVKSSFGYHIIKADKPTDFNSEKQSLKEKLVDQKVQKNPKLLTDAYKDLLKEYDVDFKDRDIKSVVEDKILNPEKLKQGGAQGGQSGMSQ

>WP_104408762.1 foldase protein PrsA [Staphylococcus aureus]

MKMINKLIVPVTASALLLGACGASATDSKENTLISSKAGDVTVADTMKKIGKDQIANASFTEMLNKILADKYKNKVNDKKIDEQIEKMQKQYGGKDKFEKALQQQGLTADKYKENLRTAAYHKELLSDKIKISDSEIKEDSKKASHILIKVKSKKSDKEGLDDKEAKQKAEEIQKEVSKDPSKFGKIAKKESMDTGSAKKDGELGYVLKGQTDKDFEKALFKLKDGEVSDVVKSSFGYHIIKADKPTDFNSEKQSLKEKLVDQKVQKNPKLLTDAYKDLLKEYDVDFKDRDIKSVVEDKILNPEKLKQGGAKGGQSGMSQ

>HDG6886278.1 peptidylprolyl isomerase [Staphylococcus aureus]

MKMINKLIVPVTASALLLGACGASATDSKENTLITSKAGDVTVADTMKKIGKDQIANASFTEMLNKILADKYKNKVNDKKIDEQIEKMQKQYGGKDKFEKALQQQGLTADKYKENLRTAAYHKELLSDKIKISDSEIKEDSKKASHILIKVKSKKSDKEGLDDKEAKQKAEEIQKEVSKDPSKFGEIAKKESMDTGSAKKDGELGYVLKGQTDKDFEKALFKLKDGEVSDVVKSSFGYHIIKADKPTDFNSEKQSLKEKLVDQKVQKNPKLLTDAYKDLLKEYDVDFKDRDIKSVVEDKILNPEKLKQGGAQGGQSGMSQ

>WP_153910638.1 foldase protein PrsA [Staphylococcus aureus]

MKMINKLIVPVTASALLLGACGASATDSKENTLISSKAGDVTVADTMKKIGKDQIANASFTEMLNKILADKYKNKVNDKKIDEQIEKMQKQYGGKDKFEKALQQQGLTADKYKENLRTAAYHKELLSDKIKISDSEIKEDSKKASHILIKVKSKKSDKEGLDDKEAKQKAEEIQKEVAKDPSKFAEIAKKESMDTGSAKKDGELGYVLKGQTDKDFEKALFKLKDGEVSDVVKSSFGYHIIKADKPTDFNSEKQSLKEKLVDQKVQKNPKLLTDAYKDLLKEYDVDFKDRDIKSVVEDKILNPEKLKQGGAQGGKSGMSQ

>HBI8815543.1 peptidylprolyl isomerase [Staphylococcus aureus]

MKMINKLIVPVTASALLLGACGASATDSKENTLISSKAGDVTVADTMKKIGKDQISNASFTEMLNKILADKYKNKVNDKKIDEQIEKMQKQYGGKDKFEKALQQQGLTADKYKENLRTAAYHKELLSDKIKISDSEIKEDSKKASHILIKVKSKKSDKEGLDDKEAKQKAEEIQKEVSKDPSKFGEIAKKESMDTGSAKKDGELGYVLKGQTDKDFEKALFKLKDGEVSDVVKSSFGYHIIKADKPTDFNSEKQSLKEKLVDQKVQKNPKLLTDAYKDLLKEYDVDFKDRDIKSVVEDKILNPEKLKQGGAQGGQSGMSQ

>HDE4914705.1 peptidylprolyl isomerase [Staphylococcus aureus]

MKMINKLIVPVTASALLLGACGASATDSKENTLISSKAGDVTVADTMKKIGKDQIANASFTEMLNKILADKYKNKVNDKKIDEQIEKMQKQYGGKDKFEKALQQQGLTADKYKENLRTAAYHKELLSDKIKISDSEIKEDSKKSSHILIKVKSKKSDKEGLDDKEAKQKAEEIQKEVSKDPSKFGEIAKKESMDTGSAKKDGELGYVLKGQTDKDFEKALFKLKDGEVSDVVKSSFGYHIIKADKPTDFNSEKQSLKEKLVDQKVQKNPKLLTDAYKDLLKEYDVDFKDRDIKSVVEDKILNPEKLKQGGAQGGQSGMSQ

>MCC0871369.1 peptidylprolyl isomerase [Staphylococcus aureus]

MKMINKLIVPVTASALLLGACGASATDSKENTLISSKAGDITVADTMKKIGKDQIANASFTEMLNKILADKYKNKVNDKKIDEQIEKMQKQYGGKDKFEKALQQQGLTADKYKENLRTAAYHKELLSDKIKISDSEIKEDSKKASHILIKVKSKKSDKEGLDDKEAKQKAEEIQKEVSKDPSKFAEIAKKESMDTGSAKKDGELGYVLKGQTDKDFEKALFKLKDGEVSDVVKSSFGYHIIKADKPTDFNSEKQSLKEKLVDQKVQKNPKLLTDAYKDLLKEYDVDFKDRDIKSVVEDKILNPEKLKQGGAQGGQSGMSQ

>HCY7093907.1 peptidylprolyl isomerase [Staphylococcus aureus]

MKMINKLIVPVTASALLLGACGASATDSKENTLISFKAGDVTVADTMKKIGKDQIANASFTEMLNKILADKYKNKVNDKKIDEQIEKMQKQYGGKDKFEKALQQQGLTADKYKENLRTAAYHKELLSDKIKISDSEIKEDSKKASHILIKVKSKKSDKEGLDDKEAKQKAEEIQKEVSKDPSKFGEIAKKESMDTGSAKKDGELGYVLKGQTDKDFEKALFKLKDGEVSDVVKSSFGYHIIKADKPTDFNSEKQSLKEKLVDQKVQKNPKLLTDAYKDLLKEYDVDFKDRDIKSVVEDKILNPEKLKQGGAQGGQSGMSQ

>WP_259378833.1 foldase protein PrsA [Staphylococcus aureus]

MKMINKLIVPVTASALLLGACGASATDSKENTLISSKAGDVTVADTMKKIGKDQIANASFTEMLNKILADKYKNKVNDKKIDEQIEKMQKQYGGKDKFEKALQQQGLTADKYKENLRTAAYHKELLSDKIKISDSEIKEDSKKASHILIKVKSKKSAKEGLDDKEAKQKAEEIQKEVSKDPSKFGEIAKKESMDTGSAKKDGELGYVLKGQTDKDFEKALFKLKDGEVSDVVKSSFGYHIIKADKPTDFNSEKQSLKEKLVDQKVQKNPKLLTDAYKDLLKEYDVDFKDRDIKSVVEDKILNPEKLKQGGAQRGQSGMSQ

>NGB68787.1 peptidylprolyl isomerase [Staphylococcus aureus]

MKMINKLIVPVTASALLLGACGASATDSKENTLISSKAGDVTVADTMKKIGKDQIANASFTEMLNKILADKYKNKVNDKKIDEQIEKMQKQYGGKDKFEKALQQQGLTADKYKENLRTAAYHKELLSDKIKISDSEIKEDSKKASHILIKVKSTKSDKEGLDDKEAKQKAEEIQKEVSKDPSKFGEIAKKESMDTGSAKKDGELGYVLKGQTDKDFEKALFKLKDGEVSDVVKSSFGYHIIKADKPTDFNSEKQSLKEKLVDQKVQKNPKLLTDAYKDLLKEYDVDFKDRDIKSVVEDKILNPEKLKQGGAQGGQSGMSQ

>HDC5784388.1 peptidylprolyl isomerase [Staphylococcus aureus]

MKMINKLIVPVTASALLLGACGASATDSKENTLISSKAGDVTVADTMKKIGKDQIANASFTEMLNKILADKYKNKVNDKKIDEQIEKMQKQYGGKDKFEKALQQQGLTADKYKENLRTDAYHKELLSDKIKISDSEIKEDSKKASHILIKVKSKKSDKEGLDDKEAKQKAEEIQKEVSKDPSKFGEIAKKESMDTGSAKKDGELGYVLKGQTDKDFEKALFKLKDGEVSDVVKSSFGYHIIKADKPTDFNSEKQSLKEKLVDQKVQKNPKLLTDAYKDLLKEYDVDFKDRDIKSVVEDKILNPEKLKQGGAQGGQSGMSQ

>WP_141060419.1 foldase protein PrsA [Staphylococcus aureus]

MKMINKLIVPVTASALLLGACGASATDSKENTLISSKAGDVTVADTMKKIGKDQIANASFTEMLNKILADKYKNKVNDKKIDEQIEKMQKQYGGKDKFEKALQQQGLTADKYKENLRTAAYHKELLSDKIKISDSEIKEDSKKASHILIKVKSKKSAKEGLDDKEAKQKAEEIQKEVSKDPSKFGEIAKKESMDTGSAKKDGELGYVLKGQTDKDFEKALFKLKDGEVSDVVKSSFGYHIIKADKPTDFNSEKQSLKEKLVDQKVQKNPKLLTDAYKDLLKEYDVDFKDRDIKSVVEDKILNPEKLKQGGAQGGQSGMSQ

>WP_070004955.1 foldase protein PrsA [Staphylococcus aureus]

MKMINKLIVPVTASALLLGACGASATDSKENTLISSKAGNVTVADTMKKIGKDQIANASFTEMLNKILADKYKNKVNDKKIDEQIEKMQKQYGGKDKFEKALQQQGLTADKYKENLRTAAYHKELLSDKIKISDSEIKEDSKKASHILIKVKSKKSDKEGLDDKEAKQKAEEIQKEVSKDPSKFGEIAKKESMDTGSAKKDGELGYVLKGQTDKDFEKALFKLKDGEVSDVVKSSFGYHIIKADKPTDFNSEKQSLKEKLVDQKVQKNPKLLTDAYKDLLKEYDVDFKDRDIKSVVEDKILNPEKLKQGGAQGGQSGMSQ

>HCZ0210440.1 peptidylprolyl isomerase [Staphylococcus aureus]

MKMINKLIVPVTASALLLGACGASATDSKENTLISSKAGDVTVADTMKKIGKDQIANASFTEMLNKILADKYKNKVNDKKIDEQIEKMQKQYGGKDKFEKALQQQGLTADKYKENLRTAAYHKELLSDKIKISDSEIKEDSKKASHILIKVKSKKSDKEGLDDKEAKQKAEEIQKEVSKDPSKFGEIAKKESMDTGSAKKNGELGYVLKGQTDKDFEKALFKLKDGEVSDVVKSSFGYHIIKADKPTDFNSEKQSLKEKLVDQKVQKNPKLLTDAYKDLLKEYDVDFKDRDIKSVVEDKILNPEKLKQGGAQGGQSGMSQ

>HCY8179126.1 peptidylprolyl isomerase [Staphylococcus aureus]

MKMINKLIVPVTASALLLGACGASATDSKENTLISSKAGDVTVADTMKKIGKDQIANASFTEMLNKILSDKYKNKVNDKKIDEQIEKMQKQYGGKDKFEKALQQQGLTADKYKENLRTAAYHKELLSDKIKISDSEIKEDSKKASHILIKVKSKKSDKEGLDDKEAKQKAEEIQKEVSKDPSKFGEIAKKESMDTGSAKKDGELGYVLKGQTDKDFEKALFKLKDGEVSDVVKSSFGYHIIKADKPTDFNSEKQSLKEKLVDQKVQKNPKLLTDAYKDLLKEYDVDFKDRDIKSVVEDKILNPEKLKQGGAQGGQSGMSQ

>HDD7189268.1 peptidylprolyl isomerase [Staphylococcus aureus]

MKMINKLIVPVTASALLLGACGASATDSKENTLISSKAGDVTVADTMKKIGKDQIANASFTEMLNKILADKYKNKVNDKKIDEQIEKMQKQYGGKDKFEKALQQQGLTADKYKENLRTAAYHKELLSDKIKISDSEIKEDSKKASHILIKVKSKKSNKEGLDDKEAKQKAEEIQKEVSKDPSKFGEIAKKESMDTGSAKKDGELGYVLKGQTDKDFEKALFKLKDGEVSDVVKSSFGYHIIKADKPTDFNSEKQSLKEKLVDQKVQKNPKLLTDAYKDLLKEYDVDFKDRDIKSVVEDKILNPEKLKQGGAQGGQSGMSQ

>HDG4985103.1 peptidylprolyl isomerase [Staphylococcus aureus]

MKMINKLIVPVTASALLLGACGASATDSKENILISSKAGDVTVADTMKKIGKDQIANASFTEMLNKILADKYKNKVNDKKIDEQIEKMQKQYGGKDKFEKALQQQGLTADKYKENLRTAAYHKELLSDKIKISDSEIKEDSKKASHILIKVKSKKSDKEGLDDKEAKQKAEEIQKEVSKDPSKFGEIAKKESMDTGSAKKDGELGYVLKGQTDKDFEKALFKLKDGEVSDVVKSSFGYHIIKADKPTDFNSEKQSLKEKLVDQKVQKNPKLLTDAYKDLLKEYDVDFKDRDIKSVVEDKILNPEKLKQGGAQGGQSGMSQ

>WP_276225107.1 foldase protein PrsA [Staphylococcus aureus]

MKMINKLIVPVTASALLLGACGASATDSKENTLISSKAGDVTVADTMKKIGKDQIANASFTEMLNKILADKYKNKVNDKKIDEQIEKMQKQYGGKDKFEKALQQQGLTADKYKENLRTAAYHKELLSDKIKISDSEIKEDSKKASHILIKVKSKKSDKEGLDDKEAKQKAEEIQKEVSKDPNKFGEIAKKESMDTGSAKKDGELGYVLKGQTDKDFEKALFKLKDGEVSDVVKSSFGYHIIKADKPTDFNSEKQSLKEKLVDQKVQKNPKLLTDAYKDLLKEYDVDFKDRDIKSVVEDKILNPEKLKQGGAQGGQSGMSQ

>MBR9069941.1 peptidylprolyl isomerase [Staphylococcus aureus]

MKMINKLIIPVTASALLLGACGASATDSKENTLISSKAGDVTVADTMKKIGKDQIANASFTEMLNKILADKYKNKVNDKKIDEQIEKMQKQYGGKDKFEKALQQQGLTADKYKENLRTAAYHKELLSDKIKISDSEIKEDSKKASHILIKVKSKKSDKEGLDDKEAKQKAEEIQKEVSKDPSKFGEIAKKESMDTGSAKKDGELGYVLKGQTDKDFEKALFKLKDGEVSDVVKSSFGYHIIKADKPTDFNSEKQSLKEKLVDQKVQKNPKLLTDAYKDLLKEYDVDFKDRDIKSVVEDKILNPEKLKQGGAQGGQSGMSQ

>HDA6899101.1 peptidylprolyl isomerase [Staphylococcus aureus]

MKMINKLIVPVTASALLLGACGASATDSKENTLISSKAGDVTVADTMKKIGKDQIANASFTEMLNKILADKYKNKVNDKKIDEQIEKMQKQYGGKDKFEKALQQQGLTADKYKENLRTAAYHKELLSDKIKISDSEIKEDSKKASHILIKVKSKKSDKEVLDDKEAKQKAEEIQKEVSKDPSKFGEIAKKESMDTGSAKKDGELGYVLKGQTDKDFEKALFKLKDGEVSDVVKSSFGYHIIKADKPTDFNSEKQSLKEKLVDQKVQKNPKLLTDAYKDLLKEYDVDFKDRDIKSVVEDKILNPEKLKQGGAQGGQSGMSQ

>WP_130108534.1 foldase protein PrsA [Staphylococcus aureus]

MKMINKLIVPVTASALLLGACGASATDSKENTLISSKAGDVTVADTMKKIGKDQIANASFTEMLNKILADKYKNKVNDKKIDEQIEKMQKQYGGKDKFEKALQQQGLTADKYKENLRTAAYHKELLSDKIKISDSEIKEDSKKASHILIKVKSKKSDKEGLDDKEAKQKAEEIQKEVSKDPSKFGEIAKKESMDTGSAKKDGELGYVLKGQTDKDFEKALFKLKDGEVSDVVKSSFGYHIIKADKPTDFNSEKQSLKEKSVDQKVQKNPKLLTDAYKDLLKEYDVDFKDRDIKSVVEDKILNPEKLKQGGAQGGQSGMSQ

>HDB6438595.1 peptidylprolyl isomerase [Staphylococcus aureus]

MKMINKLIVPVTASALLLGACGASATDSKENTLISSKAGDITVADTMKKIGKDQIANASFTEMLNKILADKYKNKVNDKKIDEQIEKMQKQYGGKDKFEKALQQQGLTADKYKENLRTAAYHKELLSDKIKISDSEIKEDSKKASHILIKVKSKKSDKEGLDDKEAKQKAEEIQKEVSKDPSKFGEIAKKESMDTGSAKKDGELGYVLKGQTDKDFEKALFKLKDGEVSDVVKSSFGYHIIKADKPTDFNSEKQSLKEKLVDQKVQKNPKLLTDAYKDLLKEYDVDFKDRDIKSVVEDKILNPEKLKQGGAQGGQSGMSQ

>HDI7446703.1 peptidylprolyl isomerase [Staphylococcus aureus]

MKMMNKLIVPVTASALLLGACGASATDSKENTLISSKAGDVTVADTMKKIGKDQIANASFTEMLNKILADKYKNKVNDKKIDEQIEKMQKQYGGKDKFEKALQQQGLTADKYKENLRTAAYHKELLSDKIKISDSEIKEDSKKASHILIKVKSKKSDKEGLDDKEAKQKAEEIQKEVSKDPSKFGEIAKKESMDTGSAKKDGELGYVLKGQTDKDFEKALFKLKDGEVSDVVKSSFGYHIIKADKPTDFNSEKQSLKEKLVDQKVQKNPKLLTDAYKDLLKEYDVDFKDRDIKSVVEDKILNPEKLKQGGAQGGQSGMSQ

>HCX0767761.1 peptidylprolyl isomerase [Staphylococcus aureus]

MKMINKLIVPVTASALLLGACGASATDSKENTLISSKAGDVTVADTMKKIGKDQIANASFTEMLNKILADKYKNKVNDKKIDEQIEKMQKQYGGKDKFEKALQQQGLTADKYKENLRTAAYHKELLSDKIKISDSEIKEDSKKASHILIKVKSKKSDKEGLDDKEAKQKAEEIQKEVSKDPSKFGEIAKKESMDTGSAKKDGELGYVLKGQTDKDFEKALFKLKDGEVSDVVKSSFGYHIIKADKPTDFNSEKQSLKEKLVDQKVQKNPKLLTNAYKDLLKEYDVDFKDRDIKSVVEDKILNPEKLKQGGAQGGQSGMSQ

>HDD2820433.1 peptidylprolyl isomerase [Staphylococcus aureus]

MKMINKLIVPVTASALLLGACGASATDSKENTLISSKAGDVTVADTMKKIGKDQIANASFTEMLNKILADKYKNKVNDKKIDEQIEKMQKQYGGKDKFEKALQQQGLTADKYKENLRTAAYHKELLSDKIKISDSEIKEDSKKASHILIKVKSKKSDKEGLDDKEAKQKAEEIQKEVSKDPSKFGEIAKKESMDTGSAKKDGELGYVLKGQTDKDFEKALFKLKDGEVSDVVKSSFGYHIIKADKPTDFNSEKQSLKEKLVDQKVQKNPKLLTDAYKDLLKEYDVNFKDRDIKSVVEDKILNPEKLKQGGAQGGQSGMSQ

>HDE0424038.1 peptidylprolyl isomerase [Staphylococcus aureus]

MKMINKLIVPVTASALLLGACGASATDSKENTLISSKSGDVTVADTMKKIGKDQIANASFTEMLNKILADKYKNKVNDKKIDEQIEKMQKQYGGKDKFEKALQQQGLTADKYKENLRTAAYHKELLSDKIKISDSEIKEDSKKASHILIKVKSKKSAKEGLDDKEAKQKAEEIQKEVSKDPSKFGEIAKKESMDTGSAKKDGELGYVLKGQTDKDFEKALFKLKDGEVSDVVKSSFGYHIIKADKPTDFNSEKQSLKEKLVDQKVQKNPKLLTDAYKDLLKEYDVDFKDRDIKSVVEDKILNPEKLKQGGAQGGQSGMSQ

>WP_111228688.1 foldase protein PrsA [Staphylococcus aureus]

MKMINKLIVPVTASALLLGACGASATDSKENTLISSKAGDVTVADTMKKIGKDQIANASFTEMLNKILADKYKNKVNDKKIDEQIEKMQKQYGGKDKFEKALQQQGLTADKYKENLRTAAYHKELLSDKIKISDSEIKEDSKKASHILIKVKSKKSDKEGLDDKEAKQKAEEIQKEVSKDPSKFGEIAKKESMDTGSAKKDGELGYVLKGQTDKDFEKALFKLKDGEVSDVVKSSFGYHIINADKPTDFNSEKQSLKEKLVDQKVQKNPKLLTDAYKDLLKEYDVDFKDRDIKSVVEDKILNPEKLKQGGAKGGQSGMSQ

>HDA2921047.1 peptidylprolyl isomerase [Staphylococcus aureus]

MKMINKLIVPVTASALLLGACGASATDSKENTLISSKAGDVTVADTMKKIGKDQIANASFTEMLNKILADKYKNKVNDKKIDEQIEKMQKQYGGKDKFEKALQQQGLTADKYKENLRTAAYHKELLSDKIKISDSEIKEDSKKASHILIKVKYKKSDKEGLDDKEAKQKAEEIQKEVSKDPSKFGEIAKKESMDTGSAKKDGELGYVLKGQTDKDFEKALFKLKDGEVSDVVKSSFGYHIIKADKPTDFNSEKQSLKEKLVDQKVQKNPKLLTDAYKDLLKEYDVDFKDRDIKSVVEDKILNPEKLKQGGAQGGQSGMSQ

>HDF6433848.1 peptidylprolyl isomerase [Staphylococcus aureus]

MKMINKLIVPVTASALLLAACGASATDSKENTLISSKAGDVTVADTMKKIGKDQIANASFTEMLNKILADKYKNKVNDKKIDEQIEKMQKQYGGKDKFEKALQQQGLTADKYKENLRTAAYHKELLSDKIKISDSEIKEDSKKASHILIKVKSKKSDKEGLDDKEAKQKAEEIQKEVSKDPSKFGEIAKKESMDTGSAKKDGELGYVLKGQTDKDFEKALFKLKDGEVSDVVKSSFGYHIIKADKPTDFNSEKQSLKEKLVDQKVQKNPKLLTDAYKDLLKEYDVDFKDRDIKSVVEDKILNPEKLKQGGAQGGQSGMSQ

>WP_103145529.1 foldase protein PrsA [Staphylococcus aureus]

MKMINKLIVPVTASALLLGACGASATDSKENTLISSKAGDVTVADTMKKIGKDQIANASFTEMLNKILADKYKNKVNDKKIDEQIEKMQKQYGGKDNFEKALQQQGLTADKYKENLRTAAYHKELLSDKIKISDSEIKEDSKKASHILIKVKSKKSDKEGLDDKEAKQKAEEIQKEVSKDPSKFGEIAKKESMDTGSAKKDGELGYVLKGQTDKDFEKALFKLKDGEVSDVVKSSFGYHIIKADKPTDFNSEKQSLKEKLVDQKVQKNPKLLTDAYKDLLKEYDVDFKDRDIKSVVEDKILNPEKLKQGGAQGGQSGMSQ

>CAC6766285.1 Foldase protein PrsA precursor [Staphylococcus aureus]

MKMINKLIVPVTASALLLGACGASATDSKENTLISSKAGDVTVADTMKKIGKDQIANASFTEMLNKILADKYKNKVNDKKIDEQIEKMQKQYGGKDKFEKALQQQGLTADKYKENLRTAAYHKELLSDKIKISDSEIKENSKKASHILIKVKSKKSDKEGLDDKEAKQKAEEIQKEVSKDPSKFGEIAKKESMDTGSAKKDGELGYVLKGQTDKDFEKALFKLKDGEVSDVVKSSFGYHIIKADKPTDFNSEKQSLKEKLVDQKVQKNPKLLTDAYKDLLKEYDVDFKDRDIKSVVEDKILNPEKLKQGGAQGGQSGMSQ

>WP_061822218.1 foldase protein PrsA [Staphylococcus aureus]

MKMINKLIVPVTASALLLGACGASATDSKENTLISSKAGDVTVADTMKKIGKDQIANASFTEMLNKILADKYKNKVNDKKIDEQIEKMQKQYGGKDKFEKALQQQGLTADKYKENLRTAAYHKELLSDKIKISDSEIKEDSKKASHILIKVKPKKSDKEGLDDKEAKQKAEEIQKEVSKDPSKFGEIAKKESMDTGSAKKDGELGYVLKGQTDKDFEKALFKLKDGEVSDVVKSSFGYHIIKADKPTDFNSEKQSLKEKLVDQKVQKNPKLLTDAYKDLLKEYDVDFKDRDIKSVVEDKILNPEKLKQGGAQGGQSGMSQ

>HEI1280801.1 peptidylprolyl isomerase [Staphylococcus aureus]

MKMINKLIVPVTASALLLGACGASATDSKENTLISSKAGDVTVADTMKKIGKDQIANASFTEMLNKILADKYKNKVNDKKIDEQIEKMQKQYGGKDKFEKALQQQGLTADKYKENLRTAAYHKELLSDKIKISDSEIKEDSKKASHILIKVKSKKSDKEGLDDKEAKQKAEEIQKEVSKDPSKFGEIAKKESMDTGSAKKDGELGYVLKGQTDKDFEKALFKLKDGEVSDVVKSSFGYHIIKADKPTDFNSEKQSLKEKLVDQKVQKNPKLLTDAYKDLLKEYDVDFKDRNIKSVVEDKILNPEKLKQGGAQGGQSGMSQ

>MCB8236914.1 peptidylprolyl isomerase [Staphylococcus aureus]

MKMINKLIVPVTASALLLGACGASATDSKENTLISSKAGDVTVADTMKKIGKDQIANASFTEMLNKILADKYKNKVNDKKIDEQIEKMQKQYGGKDKFEKALQQQGLTADKYKENLRTAAYHKELLSDKIKISDSEIKEDSKKASHILIKVKSKKSDKEGLDDKEAKQKAEEIQKEVSKDPSKFGEIAKKESMDTGSAKKDGELGYVLKGQTDKDFEKALFKLKDGEVSDVVKSSFGYHIIKADKPTDFNSEKQSLKEKLVDQKVQKNPKLLTDAYKDLLKEYDVDFKDRDIKSVVEDKILNPEKLKQGWRTRRTIRHEPITQNRATVVQISYHDRSVFSH

>WP_109028245.1 foldase protein PrsA [Staphylococcus aureus]

MKMINKLIVPVTASALLLGACGASATDSKENTLISSKAGDVTVADTMKKIGKDQIANASFTEMLNKILADKYKNKVNDKKIDEQIEKMQKQYGGKDKFEKALQQQGLTADKYKENLRTAAYHKELLSDKIKISDSEIKEDSKKASHILIKVKSKKSDKEGLDDKEAKQKAEEIQKEVSKDPSKFGEIAKKESMDTGSAKKDGELGYVLKGQTDKDFEKALFKLKDGEVSDVVKSSFGYHIVKADKPTDFNSEKQSLKEKLVDQKVQKNPKLLTDAYKDLLKEYDVDFKDRDIKSVVEDKILNPEKLKQGGAQGGQSGMSQ

>HCX1687824.1 peptidylprolyl isomerase [Staphylococcus aureus]

MKMINKLIVPVTASALLLGACGASATDSKENTLISSKAGDVTVADTMKKIGKDQIANASFTEMLNKILADKYKNKVNDKKIDEQIEKMQKQYGGKDKFEKALQQQGLTADKYKENLRTAAYHKELLSDKIKISDSEIKEDSKKASHILIKVKSKKSDKEGLDDKEAKQKAEEIQKEVSKDPSKFGEIAKKESMDTGSAKKDGELGYVLKGQTDKDFEKALFKLKDGEVSDVVKSSFGYHVIKADKPTDFNSEKQSLKEKLVDQKVQKNPKLLTDAYKDLLKEYDVDFKDRDIKSVVEDKILNPEKLKQGGAQGGQSGMSQ

>MEO4637555.1 peptidylprolyl isomerase [Staphylococcus aureus]

MKMINKLIVPVTASALLLGACGASATDSKENTLISSKAGDVTVADTMKKIGKDQIANASFTEMLNKILADKYKNKVNDKKIDEQIEKMQKQYGGKDKFEKALQQQGLTADKYKENLRTAAYHKELLSDKIKISDSEIKEDSKKASHILIKVKSKKSDKEGLDDKEAKQKAEEVQKEVSKDPSKFGEIAKKESMDTGSAKKDGELGYVLKGQTDKDFEKALFKLKDGEVSDVVKSSFGYHIIKADKPTDFNSEKQSLKEKLVDQKVQKNPKLLTDAYKDLLKEYDVDFKDRDIKSVVEDKILNPEKLKQGGAQGGQSGMSQ

>WP_070015838.1 foldase protein PrsA [Staphylococcus aureus]

MKMINKLIVPVTASALLLGACGASATDSKENTLISSKAGDVTVADTMKKIGKDQIANASFTEMLNKILADKYKNKVNDKKIDEQIEKMQKQYGGKDKFEKALQQQGLTADKYKENLRTAAYHKELLSDKIKISDSEIKEDSKKASHILIKVKSKKSDKEGLDDKEAKQKAEEIQKEVSKDPSKFGEIAKKESMDTGSAKKDGELGYVLKGQTDKDFEKALFKLKDGEVSDVVKSSFGYHIIKADKPTDFNSEKQSLKEKLVDQKVQKNPKLLTEAYKDLLKEYDVDFKDRDIKSVVEDKILNPEKLKQGGAKGGQSGMSQ

>HDJ7766468.1 peptidylprolyl isomerase [Staphylococcus aureus]

MKMINKLIVPVTASALLLGACGASATDSKENTLISSKAGDVTVADTMKKIGKDQIANASFTEMLNKILADKYKNKVNDKKIDEQIKKMQKQYGGKDKFEKALQQQGLTADKYKENLRTAAYHKELLSDKIKISDSEIKEDSKKASHILIKVKSKKSDKEGLDDKEAKQKAEEIQKEVAKDPSKFAEIAKKESMDTGSAKKDGELGYVLKGQTDKDFEKALFKLKDGEVSDVVKSSFGYHIIKADKPTDFNSEKQSLKEKLVDQKVQKNPKLLTDAYKDLLKEYDVDFKDRDIKSVVEDKILNPEKLKQGGAQGGQSGMSQ

>WP_312011137.1 peptidylprolyl isomerase [Staphylococcus aureus]

MKMINKLIVPVTASALLLGACGASATDSKENTLISSKAGDVTVADTMKKIGKDQIANASFTEMLNKILTDKYKNKVNDKKIDEQIEKMQKQYGGKDKFEKALQQQGLTADKYKENLRTAAYHKELLSDKIKISDSEIKEDSKKASHILIKVKSKKSDKEGLDDKEAKQKAEEIQKEVSKDPSKFGEIAKKESMDTGSAKKDGELGYVLKGQTDKDFEKALFKLKDGEVSDVVKSSFGYHIIKADKPTDFNSEKQSLKEKLVDQKVQKNPKLLTDAYKDLLKEYDVDFKDRDIKSVVEDKILNPEKLKQGGAQGGQSGMSQ

>MCS4729339.1 peptidylprolyl isomerase [Staphylococcus aureus]

MKMINKLIVPVTASALLLGACGASATDSKENTLISSKAGDVTVADTMKKIGKDQIANASFTEMLNKILADKYKNKVNDKKIDEQIEKMQKQYGGKDKFEKALQQQGLTADKYKENLRTTAYHKELLSDKIKISDSEIKEDSKKASHILIKVKSKKSDKEGLDDKEAKQKAEEIQKEVSKDPSKFGEIAKKESMDTGSAKKDGELGYVLKGQTDKDFEKALFKLKDGEVSDVVKSSFGYHIIKADKPTDFNSEKQSLKEKLVDQKVQKNPKLLTDAYKDLLKEYDVDFKDRDIKSVVEDKILNPEKLKQGGAQGGQSGMSQ

>WP_276210229.1 foldase protein PrsA [Staphylococcus aureus]

MKMINKLIVPVTASALLLGACGASATDSKENTLISSKAGDVTVADTMKKIGKDQIANASFTEMLNKILADKYKNKVNDKKIDEQIEKMQKQYGGKDKFEKALQQQGLTADKYKENLRTAAYHKELLSDKIKISDSEIKEDSKKASHILIKVKSKKSDKEGLDDKEAKQKAEEIQKEVSKDPSKFGEIAKKESMDTGSAKKDGELGYVLKGQTDKDFEKALFKLKDGEVSDVVKSSFGYHIIKADKPTDFNSEKQSLKEKLVDQKIQKNPKLLTDAYKDLLKEYDVDFKDRDIKSVVEDKILNPEKLKQGGVQGGQSGMSQ

>WP_315977873.1 peptidylprolyl isomerase [Staphylococcus aureus]

MKMINKLIVPVTASALLLGACGASATDSKENTLISSKAGDVTVADTMKKIGKDQIANASFTEMLNKILADKYKNKVNDKKIDEQIEKMQKQYGGKDKFEKALQQQGLTADKYKENLRTAAYHKELLSDKIKISDSEIKEDSKKASHILIKVKSKKSDKEGLDDKEAKQKAEEIQKEVSKDPSKFGEIAKKESMDTGSAKKDGELGYVLKGQTDKDFEKALFKLKDGEVSDVVKSSFGYHIIKADKPTDFNSEKQSLKEKLVDQKIQKNPKLLTDAYKDLLKEYDVDFKDRDIKSVVEDKILNPEKLKQGGAQGGQSGMSQ

>NGH56073.1 peptidylprolyl isomerase [Staphylococcus aureus]

MKMINKLIVPVTASALLLGACGASATDSKENTLISSKAGDVTVADTMKKIGKDQIANASFTEMLNKILADKYKNKVNDKKIDEQIEKMQKQYGGKDKFEKALQQQGLTADKYKENLRTAAYHKELLSDKIKISDSEIKEDSKKASHILIKVKSKKSDKEGLDDKEAKQKAEEIQKEVSKDPSKFGEIAKKESMDTGSAKKDGELGYVLKGQTDKDFEKALFKLKDGEVSDVVKSSFGYHIIKADKPTDFNSEKQSLKEKLVDQKVQKNPKLLTDAYKDLLKEYDVDFKDRDIKSVIEDKILNPEKLKQGGAQGGQSGMSQ

>WP_000782120.1 foldase protein PrsA [Staphylococcus aureus]

MKMINKLIVPVTASALLLGACGASATDSKENTLISSKAGDVTVADTMKKIGKDQIANASFTEMLNKILADKYKNKVNDKKIDEQIEKMQKQYGGKDKFEKALQQQGLTADKYKENLRTAAYHKELLSDKIKISDSEIKEDSKKASHILIKVKSKKSDKEGLDDKEAKQKAEEIQKEVSKDPSKFGEIAKKESMDTGSAKKDGELGYVLKGQTDKDFEKALFKLKDGEVSDVVKSSFGYHIIKADKPTDFNSQKQSLKEKLVDQKVQKNPKLLTDAYKDLLKEYDVDFKDRDIKSVVEDKILNPEKLKQGGAQGGQSGMSQ

>HCY8359998.1 peptidylprolyl isomerase [Staphylococcus aureus]

MKMINKLIVPVTASALLLGACGASATDSKENTLISSKAGDVTVADTMKKIGKDQIANASFTEMLNKILADKYKNKVNDKKIDEQIEKMQKQYGGKDKFEKALQQQGLTADKYKENLRTAAYHKELLSDKIKISDSEIKEDSKKASHILIKVKSKKSDKEGLDDKEAKQKAEEIQKEVSKDPSKFGEIAKKESMDTGSAKKDGELGYVLKRQTDKDFEKALFKLKDGEVSDVVKSSFGYHIIKADKPTDFNSEKQSLKEKLVDQKVQKNPKLLTDAYKDLLKEYDVDFKDRDIKSVVEDKILNPEKLKQGGAQGGQSGMSQ

>HCX9022156.1 peptidylprolyl isomerase [Staphylococcus aureus]

MKMINKLIVPVTASALLLGACGASATDSKENTLISSKAGDVTVADTMKKIGKDQIANASFTEMLNKILADKYKNKVSDKKIDEQIEKMQKQYGGKDKFEKALQQQGLTADKYKENLRTAAYHKELLSDKIKISDSEIKEDSKKASHILIKVKSKKSDKEGLDDKEAKQKAEEIQKEVSKDPSKFGEIAKKESMDTGSAKKDGELGYVLKGQTDKDFEKALFKLKDGEVSDVVKSSFGYHIIKADKPTDFNSEKQSLKEKLVDQKVQKNPKLLTDAYKDLLKEYDVDFKDRDIKSVVEDKILNPEKLKQGGAQGGQSGMSQ

>HDR0085599.1 peptidylprolyl isomerase [Staphylococcus aureus]

MKMINKLIVPVTASALLLGACGASATDSKENTLISSKAGDVTVADTMKKIGKDQIANASFTEMLNKILADKYKNKVNDKKIDEQIEKMQKQYGGKDKFEKALQQQGLTADKYKENLRTAAYHKELLPDKIKISDSEIKEDSKKASHILIKVKSKKSDKEGLDDKEAKQKAEEIQKEVSKDPSKFGEIAKKESMDTGSAKKDGELGYVLKGQTDKDFEKALFKLKDGEVSDVVKSSFGYHIIKADKPTDFNSEKQSLKEKLVDQKVQKNPKLLTDAYKDLLKEYDVDFKDRDIKSVVEDKILNPEKLKQGGAQGGQSGMSQ

>HDE0547518.1 peptidylprolyl isomerase [Staphylococcus aureus]

MKMINKLIVPVTASALLLGACGANATDSKENTLISSKAGDVTVADTMKKIGKDQIANASFTEMLNKILADKYKNKVNDKKIDEQIEKMQKQYGGKDKFEKALQQQGLTADKYKENLRTAAYHKELLSDKIKISDSEIKEDSKKASHILIKVKSKKSAKEGLDDKEAKQKAEEIQKEVSKDPSKFGEIAKKESMDTGSAKKDGELGYVLKGQTDKDFEKALFKLKDGEVSDVVKSSFGYHIIKADKPTDFNSEKQSLKEKLVDQKVQKNPKLLTDAYKDLLKEYDVDFKDRDIKSVVEDKILNPEKLKQGGAQGGQSGMSQ

>MBO8627698.1 peptidylprolyl isomerase [Staphylococcus aureus]

MKMINKLIVPVTASALLLGACGASATDSKENTLISSKAGDVTVADTMKKIGKDQIANASFTEMLNKILADKYKNKVNDKKIDEQIEKMQKQYGGKDKFKKALQQQGLTADKYKENLRTAAYHKELLSDKIKISDSEIKEDSKKASHILIKVKSKKSDKEGLDDKEAKQKAEEIQKEVSKDPSKFGEIAKKESMDTGSAKKDGELGYVLKGQTDKDFEKALFKLKDGEVSDVVKSSFGYHIIKANKPTDFNSEKQSLKEKLVDQKVQKNPKLLTDAYKDLLKEYDVDFKDRDIKSVVEDKILNPEKLKQGGAQGGQSGMSQ

>HDD6563865.1 peptidylprolyl isomerase [Staphylococcus aureus]

MKMINKLIVPVTASALLLGACGASATDSKENTLISSKAGDVTVADTMKKIGKDQIANASFTEMLNKILADKYKNKVNDKKIDEQIEKMQKQYGGKDKFEKALQQQGLTADKYKENLRTAAYHKELLSDKIKISDSEIKEDSKKASHILIKVKSKKSDKEGLDDKEAKQKAEEIQKEVSKDPSKFGEIAKKESMDTGSAKKDGELGYVLKGQTDKDFEKALFKLKDGEVSDVVKSSFGYHIIKADKPTDFNSEKQSLKEKLVDQKVQKNPKLLTDAYKDLLKEYDVDFKDHDIKSVVEDKILNPEKLKQGGAQGGQSGMSQ

>WP_101259094.1 foldase protein PrsA [Staphylococcus aureus]

MKMINKLIVPVTASALLLGACGASATDSKENTLISSKAGDVTVADTMKKIGKDQIANASFTEMLNKILADKYKNKVNDKKIDEQIEKMQKQYGGKDKFEKALQQQGLTADKYKENVRTAAYHKELLSDKIKISDSEIKEDSKKASHILIKVKSKKSDKEGLDDKEAKQKAEEIQKEVSKDPSKFGEIAKKESMDTGSAKKDGELGYVLKGQTDKDFEKALFKLKDGEVSDVVKSSFGYHIIKADKPTDFNSEKQSLKEKLVDQKVQKNPKLLTDAYKDLLKEYDVDFKDRDIKSVVEDKILNPEKLKQGGAQGGQSGMSQ

>WP_046594594.1 foldase protein PrsA [Staphylococcus aureus]

MKMINKLIVPVTASALLLGACGASATDSKENTLISSKAGDVTVADTMKKIGKDQIANASFTEMLNKILADKYKNKVNDKKIDEQIEKMQKQYGGKDKFEKALQQQGLTADKYKENLRTAAYHKELLSDKIKISDSEIKEDSKKASHILIKVKSKKSDKEGLDDKESKQKAEEIQKEVSKDPSKFGEIAKKESMDTGSAKKDGELGYVLKGQTDKDFEKALFKLKDGEVSDVVKSSFGYHIIKADKPTDFNSEKQSLKEKLVDQKVQKNPKLLTDAYKDLLKEYDVDFKDRDIKSVVEDKILNPEKLKQGGAQGGQSGMSQ

>WP_158191360.1 foldase protein PrsA [Staphylococcus aureus]

MKMINKLIVPVTASALLLGACGASATDSKENTLISSKAGDVTVADTMKKIGKDQIANASFTEMLNKILADKYKNKVNDKKIDEQIEKMQKQYGGKDKFEKALQQQGLTADKYKENLRTAAYHKELLSDKIKISDSEIKEDSKKASHILIKVKSKKSDKEGLDDKEAKQKAEEIQKDVSKDPSKFGEIAKKESMDTGSAKKDGELGYVLKGQTDKDFEKALFKLKDGEVSDVVKSSFGYHIIKADKPTDFNSEKQSLKEKLVDQKVQKNPKLLTDAYKDLLKEYDVDFKDRDIKSVVEDKILNPEKLKQGGAQGGQSGMSQ

>HDZ3345872.1 peptidylprolyl isomerase [Staphylococcus aureus]

MKMINKLIVPVTASALLLGACGACGASATDSKENTLISSKAGDVTVADTMKKIGKDQIANASFTEMLNKILADKYKNKVNDKKIDEQIEKMQKQYGGKDKFEKALQQQGLTADKYKENLRTAAYHKELLSDKIKISDSEIKEDSKKASHILIKVKSKKSDKEGLDDKEAKQKAEEIQKEVSKDPSKFGEIAKKESMDTGSAKKDGELGYVLKGQTDKDFEKALFKLKDGEVSDVVKSSFGYHIIKADKPTDFNSEKQSLKEKLVDQKVQKNPKLLTDAYKDLLKEYDVDFKDRDIKSVVEDKILNPEKLKQGSAQGGQSGMSQ

>HBC4895564.1 peptidylprolyl isomerase [Staphylococcus aureus]

MKMINKLIVPVTASALLLGACGASATDSKENTLISSKAGDVTVADTMKKIGKDQIANASFTEMLNKILADKYKNKVNDKKIDEQMEKMQKQYGGKDKFEKALQQQGLTADKYKENLRTAAYHKELLSDKIKISDSEIKEDSKKASHILIKVKSKKSDKEGLDDKEAKQKAEEIQKEVSKDPSKFGEIAKKESMDTGSAKKDGELGYVLKGQTDKDFEKALFKLKDGEVSDVVKSSFGYHIIKADKPTDFNSEKQSLKEKLVDQKVQKNPKLLTDAYKDLLKEYDVDFKDRDIKSVVEDKILNPEKLKQGGAQGGQSGMSQ

>HCZ8356864.1 peptidylprolyl isomerase [Staphylococcus aureus]

MNMINKLIVPVTASALLLGACGASATDSKENTLISSKAGDVTVADTMKKIGKDQIANASFTEMLNKILADKYKNKVNDKKIDEQIEKMQKQYGGKDKFEKALQQQGLTADKYKENLRTAAYHKELLSDKIKISDSEIKEDSKKASHILIKVKSKKSDKEGLDDKEAKQKAEEIQKEVSKDPSKFGEIAKKESMDTGSAKKDGELGYVLKGQTDKDFEKALFKLKDGEVSDVVKSSFGYHIIKADKPTDFNSEKQSLKEKLVDQKVQKNPKLLTDAYKDLLKEYDVDFKDRDIKSVVEDKILNPEKLKQGGAQGGQSGMSQ

>HDA4641525.1 peptidylprolyl isomerase [Staphylococcus aureus]

MKMINKLIVPVTASALLLGACGASATDSKENTLISSKAGDVTVADTMKKIGKDQIANASFTEMLNKILADKYKNKVNDKKIDEQIEKMQKQYGGKDKFEKALQQQGLTADKYKENLRTAAYHKELLSDKIKISDSEIKEDSKKASHILIKVKSKKSDKEGLDDKEAKQKAEEIQKEVSKDPSKFGEIAKKESMDTGSAKKNGELGYVLKGQTDKDFEKALFKLKDGEVSDVVKSSFGYHIIKADKPTDFNSEKQSLKEKLVDQKVQKNPKLLTDAYKDLLKEYDVDFKDRDIKSVVEDKILNPEKLKQGGAKGGQSGMSQ

>HDE5002998.1 peptidylprolyl isomerase [Staphylococcus aureus]

MKMINKLIVPVTASALLLGACGASATDSKENTLISSKAGDVTVADTMKKIGKDQIANASFTEMLNKILADKYKNKVNDKKIDEQIEKMQKQYGGKDKFEKALQQQGLTADKYKENLRTAAYHKELLSDKIKISDSEIKEDSKKASHILIKVKSKKSDKEGLDDKEAKQKAEEIQKEVSKDPSKFGEIAKKESMDTASAKKDGELGYVLKGQTDKDFEKALFKLKDGEVSDVVKSSFGYHIIKADKPTDFNSEKQSLKEKLVDQKVQKNPKLLTDAYKDLLKEYDVDFKDRDIKSVVEDKILNPEKLKQGGAQGGQSGMSQ

>WP_061737878.1 foldase protein PrsA [Staphylococcus aureus]

MKMINKLIVPVTASALLLGACGASATDSKENTLISSKAGDVTVADTMKKIGKDQIANASFTEMLNKILADKYKNKVNDKKIDEQIEKMQKQYGGKDKFEKALQQQGLTADKYKENLRTAAYHKELLSDKIKISDSEIKEDSKKASHILIKVKSKKSDKEGLDDKEAKQKAEEIQKEVSKDPSKFGEIAKKESMDTGSAKKDGELGYVLKGQTDKDFEKALFKLKDGEVSDVVKSSFGYHIIKADKPTDFNSEKQSLKEKLVDQKVQKNSKLLTDAYKDLLKEYDVDFKDRDIKSVVEDKILNPEKLKQGGAQGGQSGMSQ

>HBI8765835.1 peptidylprolyl isomerase [Staphylococcus aureus]

MKMINKLIVPVTASALLLSACGASATDSKENTLISSKAGDVTVADTMKKIGKDQIANASFTEMLNKILADKYKNKVNDKKIDEQIEKMQKQYGGKDKFEKALQQQGLTADKYKENLRTAAYHKELLSDKIKISDSEIKEDSKKASHILIKVKSKKSDKEGLDDKEAKQKAEEIQKEVSKDPSKFGEIAKKESMDTGSAKKDGELGYVLKGQTDKDFEKALFKLKDGEVSDVVKSSFGYHIIKADKPTDFNSEKQSLKEKLVDQKVQKNPKLLTDAYKDLLKEYDVDFKDRDIKSVVEDKILNPEKLKQGGAQGGQSGMSQ

>HDE9700097.1 peptidylprolyl isomerase [Staphylococcus aureus]

MKMINKLIVPVTASALLLGACGASATDSKENTLISSKAGDVTVADTMKKIGKDQIANASFTEMLNKILADKYKNKVNDKKIDEQIEKMQKQYGGKDKFEKALQQQGLTADKYKENLRTAAYHKELLSDKIKISDSEIKEDSKKASHILIKVKSKKSDKEGLDDKEAKQKAEEIQKEVSKDPSKFGEIAKKESMDTGSAKKDGELGYVLKGQTNKDFEKALFKLKDGEVSDVVKSSFGYHIIKADKPTDFNSEKQSLKEKLVDQKVQKNPKLLTDAYKDLLKEYDVDFKDRDIKSVVEDKILNPEKLKQGGAQGGQSGMSQ

>HCZ0159086.1 peptidylprolyl isomerase [Staphylococcus aureus]

MKMINKLIVPVTASALLLGACGASATDSKENTLISSKAGDVTVADTMKKIGKDQIANASFTEMLNKILADKYKNKVNDKKIDEQIEKMQKQYGGKDKFEKALQQQGLTADKYKENLRTAAYHKELLSDKIKISDSEIKEDSKKASHILIKVKSKKSDKEGLDDKEAKQKAEEIQKEVSKDPSKFGEIAKKESMDTGSAKKDGELGYVLKGQTDKDFEKALFKLKDGEVSDVVKSSFGYHIIKADKPTDFNSEKQSLKEKLVDQKVQKNPKLLTDAYKDLLKEYDVDFKDRDIKSVVEDKILNPEKLKQGGAQGGQSGEPITQNRATVVQKSYHGRSVFSH

>WP_061653032.1 foldase protein PrsA [Staphylococcus aureus]

MKMINKLIVPVTASALLLGACGACGASATDSKENTLISSKAGDVTVADTMKKIGKDQIANASFTEMLNKILADKYKNKVNDKKIDEQIEKMQKQYGGKDKFEKALQQQGLTADKYKENLRTAAYHKELLSDKIKISDSEIKEDSKKASHILIKVKSKKSDKEGLDDKEAKQKAEEIQKEVSKDPSKFGEIAKKESMDTGSAKKDGELGYVLKGQTDKDFEKALFKLKDGEVSDVVKSSFGYHIIKADKPTDFNSEKQSLKEKLVDQKVQKNPKLLTDAYKDLLKEYDVDFKDRDIKSVVEDKILNPEKLKQGGAQGGQSGMSQ

>HDD3915320.1 peptidylprolyl isomerase [Staphylococcus aureus]

MKMINKLIVPVTASALLLGACGASATDSKENTLISSKAGDVTVADTMKKIGKDQIANASFTEMLNKILADKYKNKVNDKKIDEQIEKMQKQYGGKDKFEKALQQQGLTADKYKENLRTAAYHKELLSDKIKISDSEIKEDSKKASHILIKVKSKKSDKEGLDDKEAKQKAEEIQKEVSKDSSKFGEIAKKESMDTGSAKKDGELGYVLKGQTDKDFEKALFKLKDGEVSDVVKSSFGYHIIKADKPTDFNSEKQSLKEKLVDQKVQKNPKLLTDAYKDLLKEYDVDFKDRDIKSVVEDKILNPEKLKQGGAQGGQSGMSQ

>WP_053015359.1 foldase protein PrsA [Staphylococcus aureus]

MKMINKLIVPVTASALLLGACGASATDSKENTLISSKAGYVTVADTMKKIGKDQIANASFTEMLNKILADKYKNKVNDKKIDEQIEKMQKQYGGKDKFEKALQQQGLTADKYKENLRTAAYHKELLSDKIKISDSEIKEDSKKASHILIKVKSKKSDKEGLDDKEAKQKAEEIQKEVSKDPSKFGEIAKKESMDTGSAKKDGELGYVLKGQTDKDFEKALFKLKDGEVSDVVKSSFGYHIIKADKPTDFNSEKQSLKEKLVDQKVQKNPKLLTDAYKDLLKEYDVDFKDRDIKSVVEDKILNPEKLKQGGAQGGQSGMSQ

>HBE7445063.1 peptidylprolyl isomerase [Staphylococcus aureus]

MKMINKLIVPVTASALLLGACGASATDSKENTLISSKAGDVTVADTMKKIGKDQIANASFTEMLNKILADKYKNKVNDKKIDEQIEKMQKQYGGKDKFEKALQQQGLTADKYKENLRTAAYHKELLSDKIKISDSEIKEDSKKASHILIKVKSKKSDKEGLDDKESKQKAEEIQKEVSKDPSKFGEIAKKESMDTGSAKKDGELGYVLKGQTDKDFEKALFKLKDGEVSDVVKSSFGYHIIKADKPTDFNSEKQSLKEKLVDQKVQKNPKLLTDAYKDLLKEYDVDFKDRDIKSVVEDKILNPEKLKQAGAQGGQSGMSQ

>HCC5685158.1 peptidylprolyl isomerase [Staphylococcus aureus]

MKMINKLIVPVTASALLLGACGASATDSKENTLISSKAGDVTVADTMKKIGKDQIANASFTEMLNKILADKYKNKVNDKKIDEQIEKMQKQYGGKDKFEKALQQQGLTADKYKENLRTAAYHKELLSDKIKISDSEIKEDSKKASHILIKVKSKKSDKEGLDDKEAKQKAEEIQKEVSKDPSKFGEIAKKESMDTGSAKKDGELGYVLKGQTDKDFEKALFKLKDGEVSDVVKSSFGYHIIKADKPTDFNSEKQSLKEKLVDQKVQKNPKLLTDAYKDLLKEYDVGFKDRDIKSVVEDKILNPEKLKQGGAQGGQSGMSQ

>HDG5637054.1 peptidylprolyl isomerase [Staphylococcus aureus]

MKMINKLIVPVTASALLLGACGASATDSKENTLISSKAGDVTVADTMKKIGKDQIANASFTEMLNKILADKYKNKVNDKKIDEQIEKMQKQYGGKDKFEKALQQQGLTADKYKENLRTAAYHKELLSDKIKISDSEIKEDSKKASHILIKVKSKKSDKEGLDDKEAKQKAEEIQKEVSKDPSKFGEIAKKESMDTGSSKKDGELGYVLKGQTDKDFEKALFKLKDGEVSDVVKSSFGYHIIKADKPTDFNSEKQSLKEKLVDQKVQKNPKLLTDAYKDLLKEYDVDFKDRDIKSVVEDKILNPEKLKQGGAQGGQSGMSQ

>HDJ2900398.1 peptidylprolyl isomerase [Staphylococcus aureus]

MKMINKLIVPVTASALLLGACGASATDSKENTLISSKAGDVTVADTMKKIGKDQIANASFTEMLNKVLADKYKNKVNDKKIDEQIEKMQKQYGGKDKFEKALQQQGLTADKYKENLRTAAYHKELLSDKIKISDSEIKEDSKKASHILIKVKSKKSDKEGLDDKEAKQKAEEIQKEVSKDPSKFGEIAKKESMDTGSAKKDGELGYVLKGQTDKDFEKALFKLKDGEVSDVVKSSFGYHIIKADKPTDFNSEKQSLKEKLVNQKVQKNPKLLTDAYKDLLKEYDVDFKDRDIKSVVEDKILNPEKLKQGGAQGGQSGMSQ

>HAS8885559.1 peptidylprolyl isomerase [Staphylococcus aureus]

MKMINKLIVPVTASALLLGACGASATDSKENTLISSKAGDVTVADTMKKIGKDQIANASFTEMLNKILADKYKNKVNDKKIDEQIEKMQKQYGGKDKFEKALQQQGLTADKYKENLRTAAYHKELLSDKIKISDSEIKEDSKKASHILIKVKSKKSDKEGLDDKEAKQKAEEIQKEVSKDPSKFGEIAKKESMDTGSAKKDGELGYVLKGQTDKDFEKALFKLKDGEVSDVVKSSFGYHIIKADKPTDFNSEKQNLKEKLVDQKVQKNPKLLTDAYKDLLKEYDVDFKDRDIKSVVEDKILNPEKLKQGGAQGGQSGMSQ

>WP_147697957.1 foldase protein PrsA [Staphylococcus aureus]

MKMINKLIVPVTASALLLGACSASATDSKENTLISSKAGDVTVADTMKKIGKDQIANASFTEMLNKILADKYKNKVNDKKIDEQIEKMQKQYGGKDKFEKALQQQGLTADKYKENLRTAAYHKELLSDKIKISDSEIKEDSKKASHILIKVKSKKSDKEGLDDKEAKQKAEEIQKEVSKDPSKFGEIAKKESMDTGSAKKDGELGYVLKGQTDKDFEKALFKLKDGEVSDVVKSSFGYHIIKADKPTDFNSEKQSLKEKLVDQKVQKNPKLLTDAYKDLLKEYDVDFKDRDIKSVVEDKILNPEKLKQGGAQGGQSGMSQ

>HDK2923778.1 peptidylprolyl isomerase [Staphylococcus aureus]

MKMINKLIVPVTASALLLGACGASATDSKENTLISSKAGDVTVADTMKKIGKDQIANASFTEMLNKILADKYKNKVNDKKIDEQIEKMQKQYGGKDKFEKALQQQGLTADKYKENLRTAAYHKELLSDKIKISDSEIKEDSKKASHILIKVKSKKSDKEGLDDKEAKQKAEEIQKEVSKDPSKFGEIAKKESMDTGSAKKDGELGYVLKGQTDKDFEKALFKLKDGEVSDVVKSSFGYHIIKADKPTDFNSEKQSLKEKLVDRKVQKNPKLLTDAYKDLLKEYDVDFKDRDIKSVVEDKILNPEKLKQGGAQGGQSGMSQ

>HCZ9336274.1 peptidylprolyl isomerase [Staphylococcus aureus]

MKMINKLIVPVTASALLLGACGASATDSKENTLISSKAGDVTVADTMKKIGKDQIANASFTEMLNKILADKYKNKVNDKKIDEQIEKMQKQYGGKDKFEKALQQQGLTADKYKENLRTAAYHKELLSDKIKISDSEIKEDSKKASHILIKVKSKKSDKEGLDDKEAKQKAEEIQKEVSKDPSKFGEIAKKESMDTGSAKKDGELGYVLKGQTDKDFEKALFKLKDGEVSDVVKSSFGYHIIKADKPTDFNSEKQSLKEKLVDQKVQKNPKLLTDAYKDLLKEYDVDFKDRDIKSVVEDKILNPEKLKQGGAQGGQSGTSQ

>EHT68326.1 foldase protein prsA [Staphylococcus aureus subsp. aureus CIG290]

MINKLIVPVTASALLLGACGASATDSKENTLISSKAGDVTVADTMKKIGKDQIANASFTEMLNKILADKYKNKVNDKKIDEQIEKMQKQYGGKDKFEKALQQQGLTADKYKENLRTAAYHKELLSDKIKISDSEIKEDSKKASHILIKVKSKKSDKEGLDDKEAKQKAEEIQKEVSKDPSKFGEIAKKESMDTGSAKKDGELGYVLKGQTDKDFEKALFKLKDGEVSDVVKSSFGYHIIKADKPTDFNSEKQSLKEKLVDQKVQKNPKLLTDAYKDLLKEYDVDFKDRDIKSVVEDKILNPEKLKQGGAQGGQSGMSQ

>WP_271019659.1 foldase protein PrsA, partial [Staphylococcus aureus]

MKMINKLIVPVTASALLLGACGASATDSKENTLISSKAGDVTVADTMKKIGKDQIANASFTEMLNKILADKYKNKVNDKKIDEQIEKMQKQYGGKDKFEKALQQQGLTADKYKENLRTAAYHKELLSDKIKISDSEIKEDSKKASHILIKVKSKKSDKEGLDDKEAKQKAEEIQKEVSKDPSKFGEIAKKESMDTGSAKKDGELGYVLKGQTDKDFEKALFKLKDGEVSDVVKSSFGYHIIKADKPTDFNSEKQSLKEKLVDQKVQKNPKLLTDAYKDLLKEYDVDFKDRDIKSVVEDKILNPEKLK

>WP_061733525.1 foldase protein PrsA [Staphylococcus aureus]

MKMLNKLIVPVTASALLLGACGASATDSKENTLISSKAGDVTVADTMKKIGKDQIANASFTEMLNKILADKYKNKVNDKKIDEQIEKMQKQYGGKDKFEKALQQQGLTADKYKENLRTAAYHKELLSDKIKISDSEIKEDSKKASHILIKVKSKKSDKEGLDDKEAKQKAEEIQKEVSKDPSKFGEIAKKESMDTGSAKKDGELGYVLKGQTDKDFEKALFKLKDGEVSDVVKSSFGYHIIKADKPTDFNSEKQSLKEKLVDQKVQKNPKLLTDAYKDLLKEYDVDFKDRDIKSVVEDKILNPEKLKQGGAQGGQSGMSQ

>HDK7619362.1 peptidylprolyl isomerase [Staphylococcus aureus]

MKMINKLIVPVTASALLLGACGASATDSKENTLISSKAGDVTVADTMKKIGKDQIANASFTEMLNKILADKYKNKVNDKKIDEQIEKMQKQYGGKDKFEKALQQQGLTADKYKENLRTAAYHKELLSDKIKISDSEIKEDSKKASHILIKVKSKKSDKEGLDDKEAKQKAEEIQKEVSKDPSKFGEIAKKESMDTGSAKKDGELGYVLKGQTDKDFEKALFKLKDGEVSDVVKSSFGYHIIKADKPTDFNNEKQSLKEKLVDQKVQKNPKLLTDAYKDLLKEYDVDFKDRDIKSVVEDKILNPEKLKQGGAQGGQSGMSQ

>HCY8775380.1 peptidylprolyl isomerase [Staphylococcus aureus]

MKMINKLIVPVTASALLLGACGASATDSKENTLISSKAGDVTVADTMKKIGKDQIANASFTEMLNKILADKYKNKVNDKKIDEQIEKMQKQYGRKDKFEKALQQQGLTADKYKENLRTAAYHKELLSDKIKISDSEIKEDSKKASHILIKVKSKKSDKEGLDDKEAKQKAEEIQKEVSKDPSKFGEIAKKESMDTGSAKKDGELGYVLKGQTDKDFEKALFKLKDGEVSDVVKSSFGYHIIKADKPTDFNSEKQSLKEKLVDQKVQKNPKLLTDAYKDLLKEYDVDFKDRDIKSVVEDKILNPEKLKQGGAQGGQSGMSQ

>MCQ1302927.1 peptidylprolyl isomerase [Staphylococcus aureus]

MKMINKLIVPVTASALLLGACGASATDSKENTLISSKAGDVTVADTMKKIGKDQIANASFTEMLNKILADKYKNKVNDKKIDEQIEKMQKQYGGKDKFEKALQQQGLTADKYKENLRTAAYHKELLSDKIKISDSEIKEDSKKASHILIKVKSKKSDKEGLDYKEAKQKAEEIQKEVSKDPSKFGEIAKKESMDTGSAKKDGELGYVLKGQTDKDFEKALFKLKDGEVSDVVKSSFGYHIIKADKPTDFNSEKQSLKEKLVDQKVQKNPKLLTDAYKDLLKEYDVDFKDRDIKSVVEDKILNPEKLKQGGAQGGQSGMSQ

>HCV2006893.1 peptidylprolyl isomerase [Staphylococcus aureus]

MKMINKLIVPVTASALLLGACGASATDSKENTLISSKAGDVTVADTMKKIGKDQIANASFTEMLNKILADKYKNKVNDKKIDEQIEKMQKQYGGKDKFEKALQQQGLTADKYKENLRTAAYHKELLSDKIKISDSEIKEYSKKASHILIKVKSKKSDKEGLDDKEAKQKAEEIQKEVSKDPSKFGEIAKKESMDTGSAKKDGELGYVLKGQTDKDFEKALFKLKDGEVSDVVKSSFGYHIIKADKPTDFNSEKQSLKEKLVDQKVQKNPKLLTDAYKDLLKEYDVDFKDRDIKSVVEDKILNPEKLKQGGAQGGQSGMSQ

>HDC9194980.1 peptidylprolyl isomerase [Staphylococcus aureus]

MKMINKLIVPVTASALLLGACGASATDSKENTLISSKAGDVTVADTMKKIGKDQIANASFTEMLNKILADKYKNKVNDKKIDEQIEKMQKQYGGKDKFEKALQRQGLTADKYKENLRTAAYHKELLSDKIKISDSEIKEDSKKASHILIKVKSKKSDKEGLDDKEAKQKAEEIQKEVSKDPSKFGEIAKKESMDTGSAKKDGELGYVLKGQTDKDFEKALFKLKDGEVSDVVKSSFGYHIIKADKPTDFNSEKQSLKEKLVDQKVQKNPKLLTDAYKDLLKEYDVDFKDRDIKSVVEDKILNPEKLKQGGAQGGQSGMSQ

>WP_217658583.1 peptidylprolyl isomerase [Staphylococcus aureus]

MKMINKLIVPVTASALLLGACGASATDSKENTLISSKAGDVTVADTMKKIGKDQIANASFTEMLNKILADKYKNKVNDKKIDEQIEKMQKQYGGKDKFEKALQQQGLTADKYKENLRTAAYHKELLSDKIKISDSEIKEDSKKASHILIKVKSKKSDKEGLYDKEAKQKAEEIQKEVSKDPSKFGEIAKKESMDTGSAKKDGELGYVLKGQTDKDFEKALFKLKDGEVSDVVKSSFGYHIIKADKPTDFNSEKQSLKEKLVDQKVQKNPKLLTDAYKDLLKEYDVDFKDRDIKSVVEDKILNPEKLKQGGAQGGQSGMSQ

>HCW9571761.1 peptidylprolyl isomerase [Staphylococcus aureus]

MKMINKLIVPVTASVLLLGACGASATDSKENTLISSKAGDVTVADTMKKIGKDQIANASFTEMLNKILADKYKNKVNDKKIDEQIEKMQKQYGGKDKFEKALQQQGLTADKYKENLRTAAYHKELLSDKIKISDSEIKEDSKKASHILIKVKSKKSDKEGLDDKEAKQKAEEIQKEVSKDPSKFGEIAKKESMDTGSAKKDGELGYVLKGQTDKDFEKALFKLKDGEVSDVVKSSFGYHIIKADKPTDFNSEKQSLKEKLVDQKVQKNPKLLTDAYKDLLKEYDVDFKDRDIKSVVEDKILNPEKLKQGGAQGGQSGMSQ

>HEK7082779.1 peptidylprolyl isomerase [Staphylococcus aureus]

MKMINKLIVPVTASALLLGACGASATDSKENTLISSKAGDVTVADTMKKIGKDQIANASFTEMLNKILADKYKNKVNDKKIDEQIEKMQKQYGDKDKFEKALQQQGLTADKYKENLRTAAYHKELLSDKIKISDSEIKEDSKKASHILIKVKSKKSDKEGLDDKEAKQKAEEIQKEVSKDPSKFGEIAKKESMDTGSAKKDGELGYVLKGQTDKDFEKALFKLKDGEVSDVVKSSFGYHIIKADKPTDFNSEKQSLKEKLVDQKVQKNPKLLTDAYKDLLKEYDVDFKDRDIKSVVEDKILNPEKLKQGGAQGGQSGMSQ

>HCZ8722847.1 peptidylprolyl isomerase [Staphylococcus aureus]

MKMINKLIVPVTASALLLGACGASATDSKENTLISSKAGDVTVADTMKKIGKDQIANASFTEMLNKILADKYKNKVNDKKIDEQIEKMQKQYGGKDKFEKALQQQGLTADKYKENLRTAAYHKELLSDKIKISDSEIKEDSKKASHILIKVKSKKSDKEGLDDKEAKQKAEEIQKEVSKDPSKFGEIAKKESMDTGSAKKDGELGYVLKGQTDKDFEKALFKLKDGEVSDVVKSSFGYHIIKADKPTDFNSEKQSLKEKLVDQKVQKNPKLLTDAYKDLLKEYDVDFKDRDIKSVVEVKILNPEKLKQGGAQGGQSGMSQ

>HCY8922826.1 peptidylprolyl isomerase [Staphylococcus aureus]

MKMINKLIVPVTASALLLGACGASATDSKENTLISSKAGDVTVADTMKKIGKDQIANASFTEMLNKILADKYKNKVNDKKIDEQIEKMQKQYGGKDKFEKALQQQGLTADKYKENLRTAAYHKELLSDKIKISDSEIKEDSKKASHILIKVKSKKSDKEGLDDKEAKQKAEEIQKEVSKDPSKFGEIAKKESMDTGSAKKDGELGYVLKGQTDKDFEKALFKLKDGEVSDVVKSSFGYHIIKADKPTDFNSEKQSLKEKLVDQKVQKNPKLLTDAYKDLLKEYDVDFKDRDIKSVVEDKILNPEKLKQGGTQGGQSGMSQ

>HDE0407807.1 peptidylprolyl isomerase [Staphylococcus aureus]

MKMINKLIVPVTASALLLGACGASATDSKENTLISSKAGDVTVADTMKKIGKDQIANASFTEMLNKILADKYKNKVNDKKIDEQIEKMQKQYGGKDKFEKALQQQGLTADKYKENLRTAAYHKELLSDKIKISDSEIKEDSKKASHILIKVKSKKSDKEGLDDKEAKQKAEEIQKEVSKDPSKFGEIAKKESMDTGSAKKDGELGYVLKGQTDKDFEKALFKLKDGEVSDVVKSSFGYHIIKADKPTDFNSEKQSLKEKLVDQKVQKNPKLLTDAYKDLLKEYDVDFKDRDIKSVVEDKILNPEKLKQGGAQGGQFGMSQ

>WP_162636609.1 foldase protein PrsA [Staphylococcus aureus]

MKMINKLIVPVTASALLLGACGASATDSKENTLISSKAGDVTVADTMKKIGKDQIANASFTEMLNKILADKYKNKVNDKKIDEQIEKMQKQYGGKDKFEKALQQQGLTADKYKENLRTAAYHKELLSDKIKISDSEIKEDSKKASHILIKVKSKKSDKEGLDDKEAKQKAEEIQKEVSKDPSKFGEIAKKESMDTGSAKKDGELGYVLKGQTDKDFEKALFKLKDGEVSDVVKSSFGYHIIKADKPTDFNSEKQSLKEKLVDQKVQKNPKLLTDAYKDLLKEYDVDFKDRDIKSVVEDKILNPEKLKQGGAQGGQPGMSQ

>HDA2810805.1 peptidylprolyl isomerase [Staphylococcus aureus]

MKMINKLIVPVTASALLLGACGASATDSKENTLISSKAGDVTVADTMKKIGKDQIANASFTEMLNKILADKYKNKVNDKKIDEQIEKMQKQYGGKDKFEKALQQQGLTADKYKENLRTAAYHKELLSDKIKISDSEIKEDSKKASHILIKVKSKKSDKEGLDDKEAKQKAEEIQKEVSKDPSKFGEIAKKESMDTGSAKKDGELGYVLKGQTDKDFEKALFKLKDGEVSDVVKSSFGYHIIKADKPTDFNSEKQSLKEKLVDQKVQKNPKLLTDAYKDLLKEYDVDFKDRDIKSVVEDKILNPEKLKQGGAQGGQAGMSQ

>WP_000782119.1 MULTISPECIES: foldase protein PrsA [Staphylococcus]

MKMINKLIVPVTASALLLGACGASATDSKENTLISSKAGDVTVADTMKKIGKDQIANASFTEMLNKILADKYKNKVNDKKIDEQIEKMQKQYGGKDKFEKALQQQGLTADKYKENLRTAAYHKELLSDKIKISDSEIKEDSKKASHILIKVKSKKSDKEGLDDKEAKQKAEEIQKEVSKDPSKFGEIAKKESMDTGSAKKDGELGYVLKGQTDKDFEKALFKLKDGEVSDVVKSSFGYHIIKADKPTDFNSEKQSLKEKLVDQKVQKNPKLLTDAYKDLLKEYDVDFKDRDIKSVVEDKILNPEKLKQGGAQGGQSGMSQ

>HCU0762850.1 peptidylprolyl isomerase [Staphylococcus aureus]

MKMINKLIVPVTASALLLGACGASATDSKENTLISSKAGDVTVADTMKKIGKDQIANASFTEMLNKILADKYKNKVNDKKIDEQIEKMQKQYGGKDKFEKALQQQGLTADKYKENLRTAAYHKELLSDKIKISDSEIKEDSKKASHILIKVKSKKSDKEGLDDKEAKQKAEEIQKEVSKDPSKFGEIAKKESMDTGSAKKDGELGYVLKGQTDKDFEKALFKLKDGEVSDVVKSSFGYHIIKADKPTDFNSEKQSLKEKLVDQKVQKNPKLLTDAYKDLLKEYDVDFKDRDIKSVVEDKILNPEKLKQGGAQGGQSG

>HCZ0161917.1 peptidylprolyl isomerase [Staphylococcus aureus]

MKMINKLIVPVTASALLLGACGASATDSKENTLISSKAGDVTVADTMKKIGKDQIANASFTEMLNKILADKYKNKVNDKKIDEQIEKMQKQYGGKDKFEKALQQQGLTADKYKENLRTAAYHKELLSDKIKISDSEIKEDSKKASHILIKVKSKKSDKEGLDDKEAKQKAEEIQKEVSKDPSKFGEIAKKESMDTGSAKKDGELGYVLKGQTDKDFEKALFKLKDGEVSDVVKSSFGYHIIKADKPTDFNSEKQSLKEKLVDQKVQKNPKLLTDAYKDLLKEYDVDFKDRDIKSVVEDKILNSEKLKQGGAQGGQSGMSQ

>HCY7137699.1 peptidylprolyl isomerase [Staphylococcus aureus]

MKMINKLIVPVTASALLLGACGASATDSKENTLISSKAGDVTVADTMKKIGKDQIANASFTEMLNKILADKYKNKVNDKKIDEQIEKMQKQYGGKDKFEKALQQQGLTADKYKENLRTAAYHKELLSDKIKISDSEIKEDSKKASHILIKVKSKKSDKEGLDDKEAKQKAEEIQKEVSKDPSKFGEIAKKESMDTGSAKKDGELGYVLKGQTDKDFEKALFKLKDGEVSDVVKSSFGYHIIKADKPTDFNSEKQSLKEKLVDQKVQKNPKLLTDAYKDLLKEYDVDFKDRDIKSVVEDKILTPEKLKQGGAQGGQSGMSQ

>WP_115395705.1 foldase protein PrsA [Staphylococcus aureus]

MKMINKLIVPVTASALLLGACGASATDSKENTLISSKAGDVTVADTMKKIGKDQIANASFTEMLNKILADKYKNKVNDKKIDEQIEKMQKQYGGKDKFEKALQQQGLTADKYKENLRTAAYHKELLSDKIKISDSEIKKDSKKASHILIKVKSKKSDKEGLDDKEAKQKAEEIQKEVSKDPSKFGEIAKKESMDTGSAKKDGELGYVLKGQTDKDFEKALFKLKDGEVSDVVKSSFGYHIIKADKPTDFNSEKQSLKEKLVDQKVQKNPKLLTDAYKDLLKEYDVDFKDRDIKSVVEDKILNPEKLKQGGAQGGQSGMSQ

>WP_061658908.1 foldase protein PrsA [Staphylococcus aureus]

MKMINKLIVPVTASALLLGACGASATDSKENTLISSKAGDVTVADTMKKIGKDQIANASFTEMLNKILADKYKNKVNDKKIDEQIEKMQKQYGGKDKFEKALQQQGLTADKYKENLRTAAYHKELLSDKIKISDSEIKEDSKKASHILIKVKSKKSDKEGLDDKEAKQKAEEIQKEVSKDPSKFGEIAKKESMDTGSAKKDGELGYVLKGQTDKDFEKALFKLKDGEVSDVVKSSFGYHIIKADKPTDFNSKKQSLKEKLVDQKVQKNPKLLTDAYKDLLKEYDVDFKDRDIKSVVEDKILNPEKLKQGGAQGGQSGMSQ

>HCY7618235.1 peptidylprolyl isomerase [Staphylococcus aureus]

MKMINKLIVPVTASALLLGACGASATDSKENTLISSKAGDVTVADTMKKIGKDQIANASFTEMLNKILADKYKNKVNDKKIDEQIEKMQKQYGGKDKFEKALQQQGLTADKYKENLRTAAYHKELLSDKIKISDSEIKEDSKKASHILIKVKSKKSDKEGLDDKEAKQKAEEIQKEVSKDPSKFGEIAKKESMDTGSAKKDGELGYVLKGQTDKDFEKALFKLKDGEVSDVVKSSFGYHIIKADKPTDFNSEKQSLKEKLVDQKVQKNPKLLTDAYKDLLKEYDVDFKDRDIKSVVEDRILNPEKLKQGGAQGGQSGMSQ

>HDE8886582.1 peptidylprolyl isomerase [Staphylococcus aureus]

MKMINKLIVPVTASALLLGACGASATDSKENTLISSKAGDVTVADTMKKIGKDQIANASFTEMLNKILADKYKNKVNDKKIDEQIEKMQKQYGGKDKFEKALQQQGLTADKYKENLRTAAYHKELLSDKIKISDSEIKEDSKKASHILIKVKSKKSDKEGLDDKEAKQKAEEIQKEVSKDPSKFGEIAKKESMDTGSAKKDGELGYVLKGQTDKDFEKALFKLKDGEVSDVVKSSFGYHIIKADKPTDFNSEKQSLKEKLVDQKVQKNPRLLTDAYKDLLKEYDVDFKDRDIKSVVEDKILNPEKLKQGGAQGGQSGMSQ

>HDA7373573.1 peptidylprolyl isomerase [Staphylococcus aureus]

MKMINKLIVPVTASALLLGACGASATDSKENTLISSKAGDVTVADTMKKIGKDQIANASFTEMLNKILADKYKNKVNDKKIDEQIEKMQKQYGGKDKFEKALQQQGLTADKYKENLRTAAYHKELLSDKIKISDSEIKEDSKKASHILIKVKSKKSDKEGLDDKEAKQKAEEIQKEVSKDPSKFGEIAKKESMDTGSAKKDGELGYVLKGQTDKDFEKALFKLKDGEVSDVVKSSFGYHIIKADKPTDFNSEKQSLKEKLVDQKVQKNPKLLTDAYKDLLKEYDVDFKDRDIKSVVEDKILNPEKLKQGGAQGGQSGISQ

>MBH4578867.1 peptidylprolyl isomerase [Staphylococcus aureus]

MKMINKLIVPVTASALLLGACGASATDSKENTLISSKAGDVTVADTMKKIGKDQIANASFTEMLNKILADKYKNKVNDKKIDEQIEKMQKQYGGKDKFEKTLQQQGLTADKYKENLRTAAYHKELLSDKIKISDSEIKEDSKKASHILIKVKSKKSDKEGLDDKEAKQKAEEIQKEVSKDPSKFGEIAKKESMDTGSAKKDGELGYVLKGQTDKDFEKALFKLKDGEVSDVVKSSFGYHIIKADKPTDFNSEKQSLKEKLVDQKVQKNPKLLTDAYKDLLKEYDVDFKDRDIKSVVEDKILNPEKLKQGSAQGGQSGMSQ

>HCT9624121.1 peptidylprolyl isomerase [Staphylococcus aureus]

MKMINKLIVPVTASALLLGACGASATDSKENTLISSKAGDVTVADTMKKIGKDQIANASFTEMLNKILADKYKNKVNDKKIDEQIEKMQKQYGGKDKFEKALQQQGLTADKYKENLRTAAYHKELLSDKIKISDSEIKEDSKKASHILIKVKSKKSDKEGLDDKEAKQKAEEIQKEVSKDPSKFGEIAKKESMDTGSAKKDGELGYVLKGQTDKDFEKALFKLKDGEVSDVVKSSFGYHIIKADKPTDFNSEKQSLKEKLVDQKVQKNPKLLTDAYKDLLKEYDVDFKDRDIKSVVEDKILNPEKLKQGGAQGGQSGMS

>HCY3101362.1 peptidylprolyl isomerase [Staphylococcus aureus]

MKMINKLIVPVTASALLLGACGASATDSKENTLISSKAGDVTVADTMKKIGKDQIANASFTEMLNKILADKYKNKVNDKKIDEQIEKMQKQYGGKDKFEKALQQQGLTADKYKENLRTAAFHKELLSDKIKISDSEIKEDSKKASHILIKVKSKKSDKEGLDDKEAKQKAEEIQKEVSKDPSKFGEIAKKESMDTGSAKKDGELGYVLKGQTDKDFEKALFKLKDGEVSDVVKSSFGYHIIKADKPTDFNSEKQSLKEKLVDQKVQKNPKLLTDAYKDLLKEYDVDFKDRDIKSVVEDKILNPEKLKQGGAQGGQSGMSQ

>CAC5440607.1 Foldase protein PrsA precursor [Staphylococcus aureus]

MKMINKLIVPVTASALLLGACGASATDSKENTLISSKAGDVTVADTMKKIGKDQIANASFTEMLNKILADKYKNKVNDKKIDEQIEKMQKQYGGKDKFEKALQQQGLTADKYKENLRTAAYHKELLSDKIKISDSEIKEDSKKASHILIKVKSKKSDKEGLDDKEAKQKAEEIQKEVSKDPSKFGEIAKKESMDTGSAKKDGELGYVLKGQTDKDFEKALFKLKDGEVSDVVKSSFGYHIIKADKPTDFNSEKQSLKEKLVDQKVQKNPKLLTDAYKDLLKEYDVDFKDRDIKSVVEDKILNPEKLKQGGAQVGQSGMSQ

>HDP3061562.1 peptidylprolyl isomerase [Staphylococcus aureus]

MKMINKLIVPVTASALLLGACGASATDSKENTLISSKAGDVTVADTMKKIGKDQIANASFTEMLNKILADKYKNKVNDKKIDEQIEKMQKQYGGKDKFEKALQQQGLTADKYKENLRTAAYHKELLSDKIKISDSEIKEDSKKASHILIKVKSKKSDKEGLDDKEAKQKAEEIQKEVSKDPSKFGEIAKKESMDTGSAKKDGELGYVLKGQTDKDFEKALFKLKDGEVSDVVKSSFGYHIIKADKPTDFNSEKQSLKEKLVDQKVQKNPKLLTDAYKDLLKEFDVDFKDRDIKSVVEDKILNPEKLKQGGAQGGQSGMSQ

>HDP2960921.1 peptidylprolyl isomerase [Staphylococcus aureus]

MKMINKLIVPVTASALLLGACGASATDSKENTLISSKAGDVTVADTMKKIGKDQIANASFTEMLNKILADKYKNKVNDKKIDEQIEKMQKQYGGKDKFEKALQQQGLTADKYKENLRTAAYHKELLSDKIKISDSEIKEDSKKASHILIKVKSKKSDKEGLDDKEAKQKAEEIQKEVSKDPSKFGEIAKKESMDTGSAKKDGELGYVLKGQTDKDFEKALFKLKDGEVSDVVKSSFGYHIIKADKPTDFNSEKQSLKEKLVDQKVQKNPKLLTDAYKDLLKEYDVDFKDRDIKSVVEDKILNPEKLKQGGAADNPA

>HCY1633049.1 peptidylprolyl isomerase [Staphylococcus aureus]

MKMINKLIVPVTASALLLGACGASATDSKENTLISSKAGDVTVADTMKKIGKDQIANASFTEMLNKILADKYKNKVNDKKIDEQIEKMQKQYGGKDKFEKALQQQGLTADKYKENLRTAAYHKELLSDKIKISDSEIKEDSKKASHILIKVKSKKSDKEGLDDKEAKQKAEEIQKEVSKDPSKFGEIAKKESMDTGSAKKDGELGYVLKGQTDKDFEKALFKLKDGEVSDVVKSSFGYHIIKADKPTDFNSEKQSLKEKLVDQKVQKNPKLLTDAYKDLLKEYDVDFKDRDIKSVVEDKILNPEKLKQGGAQGGAQGGQSGMSQ

>HDZ8658194.1 peptidylprolyl isomerase [Staphylococcus aureus]

MKMINKLIVPVTASALLLGACGASTTDSKENTLISSKAGDVTVADTMKKIGKDQIANASFTEMLNKILADKYKNKVNDKKIDEQIEKMQKQYGGKDKFEKALQQQGLTADKYKENLRTAAYHKELLSDKIKISDSEIKEDSKKASHILIKVKSKKSDKEGLDDKEAKQKAEEIQKEVSKDPSKFGEIAKKESMDTGSAKKDGELGYVLKGQTDKDFEKALFKLKDGEVSDVVKSSFGYHIIKADKPTDFNSEKQSLKEKLVDQKVQKNPKLLTDAYKDLLKEYDVDFKDRDIKSVVEDKILNPEKLKQGGAQGGQSGMSQ

>ELK6828265.1 peptidylprolyl isomerase [Staphylococcus aureus]

MKMINKLIVPVTASALLLGACGASATDSKENTLISSKAGDVTVADTMKKIGKDQIANASFTEMLNKILADKYKNKVNDKKIDEQIEKMQKQYGGKDKFEKALQQQGLTADKYKENLRTAAYHKELLSDKIKISDSEIKEDSKKTSHILIKVKSKKSDKEGLDDKEAKQKAEEIQKEVSKDPSKFGEIAKKESMDTGSAKKDGELGYVLKGQTDKDFEKALFKLKDGEVSDVVKSSFGYHIIKADKPTDFNSEKQSLKEKLVDQKVQKNPKLLTDAYKDLLKEYDVDFKDRDIKSVVEDKILNPEKLKQGGAQGGQSGMSQ

>HEB2157290.1 peptidylprolyl isomerase [Staphylococcus aureus]

MKMINKLIVPVTASALLLGACGASATDSKENTLISSKAGDVTVADTMKKIGKDQIANASFTEMLNKILADKYKNKVNDKKIDEQIEKMQKQYGGKDKFEKALQQQGLTADKYKENLRTATYHKELLSDKIKISDSEIKEDSKKASHILIKVKSKKSDKEGLDDKEAKQKAEEIQKEVSKDPSKFGEIAKKESMDTGSAKKDGELGYVLKGQTDKDFEKALFKLKDGEVSDVVKSSFGYHIIKADKPTDFNSEKQSLKEKLVDQKVQKNPKLLTDAYKDLLKEYDVDFKDRDIKSVVEDKILNPEKLKQGGAQGGQSGMSQ

>HCU2196143.1 peptidylprolyl isomerase [Staphylococcus aureus]

MKMINKLIVPVTASALLLGACGASATDSKENTLISSKAGDVTVADTMKKIGKDQIANASFTEMLNKILADKYKNKVNDKKIDEQIEKMQKQYGGKDKFEKALQQQGLTADKYKENLRTAAYHKELLSDKIKISDSEIKEDSKKASHILIKVKSKKSDKEGLDDKEAKQKAEEIQKEVSKDPSKFGEIAKKESMDTGSAKKDGELGYVLKGQTDKDFEKALFKLKDGEVSDVVKSSFGYHIIKADKPTDFNSEKQSLKEKLVDQKVQKNPKLLTDAYKDLLKEYDVDFKDRDIKSVVEDKILNPEKLKQGGAQGGQSGM

>WP_111141916.1 foldase protein PrsA [Staphylococcus aureus]

MKMINKLIVPVTASALLLGACGASATDSKENTLISSKAGDVTVADTMKKIGKDQIANASFTEMLNKILADKYKNKVNDKKIDEQIEKMQKQYGGKDKFEKALQQQGLTADKYKENLRTAAYHKELLSDKIKISDSEIKEDSKKASHILIKVKSKKSDKEGLDDKEAKQKAEEIQKEDSKDPSKFGEIAKKESMDTGSAKKDGELGYVLKGQTDKDFEKALFKLKDGEVSDVVKSSFGYHIIKADKPTDFNSEKQSLKEKLVDQKVQKNPKLLTDAYKDLLKEYDVDFKDRDIKSVVEDKILNPEKLKQGGAQGGQSGMSQ

>HCV0561901.1 peptidylprolyl isomerase [Staphylococcus aureus]

MKMINKLIVPVTASALLLGACGASATDSKENTLISSKAGDVTVADTMKKIGKDQIANASFTEMLNKILADKYKNKVNDKKIDEQIEKMQKQYGGKDKFEKALQQQGLTADKYKENLRTAAYHKELLSDKIKISDSEIKEDSKKASHILIKVKSKKSDKEGLDDKKAKQKAEEIQKEVSKDPSKFGEIAKKESMDTGSAKKDGELGYVLKGQTDKDFEKALFKLKDGEVSDVVKSSFGYHIIKADKPTDFNSEKQSLKEKLVDQKVQKNPKLLTDAYKDLLKEYDVDFKDRDIKSVVEDKILNPEKLKQGGAQGGQSGMSQ

>WP_160199685.1 foldase protein PrsA [Staphylococcus aureus]

MKMINKLIVPVTASALLLGACGASATDSKENTLISSKAGDVTVADTMKKIGKDQIANASFTEMLNKILADKYKNKVNDKKIDEQIEKMQKQYGGKDKFEKALQQQGLTADKYKENLRTAAYHKELLSDKIKISDSEIKEDSKKASHILIKVKSKKSDKEGLDDKEAKQKAEEIQKEVSKDPSKFGEIAKKESMDTGSAKKDGELGYVLKGQTDKDFEKALFKLKDGEVSDVVKSSFGYHIIKADKPTDFNSEKQSLKEKLVDQKVQKNPKLLTDAYKDLLKEYDVDFKDRDIKSVVEDKILNPEKLKQGSAQGGQSGMSQ

>HDC7889623.1 peptidylprolyl isomerase [Staphylococcus aureus]

MKMINKLIVPVTASALLLGACGASATDSKENTLISSKAGDVTVADTMKKIGKDQIANASFTEMLNKILADKYKNKVNDKKIDEQIEKMQKQYGGKDKFEKALQQQGLTADKYKENLRTAAYHKELLSDKIKISDSEIKEDSKKASHILIKVKSKKSDKEGLDDKEAKQKAEEIQKEVSKDPSKFGEIAKKESMDTGSAKKDGELGYVLKGQTDKDFEKALFKLKDGEVSDVVKSSFGYHIIKADKPTDFNSEKQSLKEKLVDQKVQKNPKLLTDAYKDLLKEYDVDFKDRDIKSVVEDKILNPEKLKQSGAQGGQSGMSQ

>WP_160194563.1 foldase protein PrsA [Staphylococcus aureus]

MKMINKLIVPVTASALLLGACGASATDSKENTLISSKAGDVTVADTMKKIGKDQIANASFTEMLNKILADKYKNKVNDKKIDEQIEKMQKQYGGKDKFEKALQQQGLTADKYKENLRTAAYHKELLSDKIKISDSEIKEDSKKASHILIKVKSKKSDKEGLDDKEAKQKAEEIQKEVSKDPSKFGEIAKKESMDTGSAKKDGELGYVLKGQTDKDFEKALFKLKDGEVSDVVKSSFGYHIIKADKPTDFNSEKQSLKEKLVDQKVQKNPKLLTDAYKDLLKEYDVDFKDRDIKSVVEDKILNPEKLKQGGAQGGQSSMSQ

>HCT9610095.1 peptidylprolyl isomerase [Staphylococcus aureus]

MKMINKLIVPVTASALLLGACGASATDSKENTLISSKAGDVTVADTMKKIGKDQIANASFTEMLNKILADKYKNKVNDKKIDEQIEKMQKQYGGKDKFEKALQQQGLTADKYKENLRTAAYHKELLSDKIKISDSEIKEDSKKASHILIKVKSKKSDKEGLDDKEAKQKAEEIQKEVSKDPSKFGEIAKKESMDTGSAKKDGELGYVLKGQTDKDFEKALFKLKDGEVSDVVKSSFGYHIIKADKPTDFNSEKQSLKEKLVDQKVQKNPKLLTDAYKDLLKEYDVDFKDRDIKSVVEDKILNPKKLKQGGAQGGQSGMSQ

>WP_111187472.1 foldase protein PrsA [Staphylococcus aureus]

MKMINKLIVPVTASALLLGACGASATDSKENTLISSKAGDVTVADTMKKIGKDQIANASFTEMLNKILADKYKNKVNDKKIDEQIEKMQKQYGGKDKFEKALQQQGLTADKYKEKLRTAAYHKELLSDKIKISDSEIKEDSKKASHILIKVKSKKSDKEGLDDKEAKQKAEEIQKEVSKDPSKFGEIAKKESMDTGSAKKDGELGYVLKGQTDKDFEKALFKLKDGEVSDVVKSSFGYHIIKADKPTDFNSEKQSLKEKLVDQKVQKNPKLLTDAYKDLLKEYDVDFKDRDIKSVVEDKILNPEKLKQGGAQGGQSGMSQ

>HDA3716585.1 peptidylprolyl isomerase [Staphylococcus aureus]

MKMINKLIVPVTASALLLGACGASATDSKENTLISSKAGDVTVADTMKKIGKDQIANASFTEMLNKILADKYKNKVNDKKIDEQIEKMQKQYGGKDKFEKALQHQGLTADKYKENLRTAAYHKELLSDKIKISDSEIKEDSKKASHILIKVKSKKSDKEGLDDKEAKQKAEEIQKEVSKDPSKFGEIAKKESMDTGSAKKDGELGYVLKGQTDKDFEKALFKLKDGEVSDVVKSSFGYHIIKADKPTDFNSEKQSLKEKLVDQKVQKNPKLLTDAYKDLLKEYDVDFKDRDIKSVVEDKILNPEKLKQGGAQGGQSGMSQ

>HCW9513267.1 peptidylprolyl isomerase [Staphylococcus aureus]

MKMINKLIVPVTASALLLGACGASATDSKENTLISSKAGDVTVADTMKKIGKDQIANASFTEMLNKILADKYKNKVNDKKIDEQIEKMQKQYGGKDKFEKALQQQGLTADKYKENLRTAAYHKELLSDKIKISDSEIKEDSKKASHILIKVKSKKSDKEGLDDKEAKQKAEEIQKEVSKDPSKFGEIAKKESMDTGSAKKDGELGYVLKGQTDKDFEKALFKLKDGEVSDVVKSSFGYHIIKADKPTDFNSEKQSLKEKLVDQKVQKNPKLLTDAYKDLLKEYDVDFKDRDIKSVVEDKILNPEKLKQGGA

>WP_021758269.1 foldase protein PrsA [Staphylococcus aureus]

MKMINKLIVPVTASALLLGACGASATDSKENTLISSKAGDVTVADTMKKIGKDQIANASFTEMLNKILADKYKNKVNDKKIDEQIEKMQKQYGGKDKFEKALQQQGLTADKYKENLRTAAYHKELLSDKIKISDSEIKEDSKKASHILIKVKSKKSDKEGLDDKEAKQKAEEIQKEVSKDPSKFGEIAKKESMDTGSAKKDGELGYVLKGQTDKDFEKALFKLKDGEVSDVVKSSFGYHIIKADKPTDFNSEKQSLKEKLVDQKVQKNPKLLTDTYKDLLKEYDVDFKDRDIKSVVEDKILNPEKLKQGGAQGGQSGMSQ

>WP_316133390.1 peptidylprolyl isomerase [Staphylococcus aureus]

MKMINKLIVPVTASALLLGACGASATDSKENTLISSKAGDVTVADTMKKIGKDQIANTSFTEMLNKILADKYKNKVNDKKIDEQIEKMQKQYGGKDKFEKALQQQGLTADKYKENLRTAAYHKELLSDKIKISDSEIKEDSKKASHILIKVKSKKSDKEGLDDKEAKQKAEEIQKEVSKDPSKFGEIAKKESMDTGSAKKDGELGYVLKGQTDKDFEKALFKLKDGEVSDVVKSSFGYHIIKADKPTDFNSEKQSLKEKLVDQKVQKNPKLLTDAYKDLLKEYDVDFKDRDIKSVVEDKILNPEKLKQGGAQGGQSGMSQ

>WP_138742480.1 foldase protein PrsA [Staphylococcus aureus]

MKMINKLIVPVTASALLLGACGASATDSKENTLISSKAGDVTVADTMKKIGKDQIANASFTEMLNKILADKYKNKVNDKKIDEQIEKMQKQYGGKDKFEKALQQQGLTADKYKENLRTAAYHKELLSDKIKISDSEIKEDSKKASHILIKVKSKKSDKEGLDDKEAKQKAEEIQKEVSKDPSKFGEIAKKESMDTGSAKKDGELGYVLKGQTDKDFEKALFKLKDGEVSDVVKSSFGYHIIKADKPTDFNSEKQSLKEKLVDQKVQKNPKLLTDAYKDLLKEYDVDFKDRDIKSVVEDKILNPEKLKQGGAQGRQSGMSQ

>HCW9825429.1 peptidylprolyl isomerase [Staphylococcus aureus]

MKMINKLIVPVTASALLLGACGASATDSKENTLISSKAGDVTVADTMKKIGKDQIANASFTEMLNKILADKYKNKVNDKKIDEQIEKMQKQYGGKDKFEKALQQQGLTTDKYKENLRTAAYHKELLSDKIKISDSEIKEDSKKASHILIKVKSKKSDKEGLDDKEAKQKAEEIQKEVSKDPSKFGEIAKKESMDTGSAKKDGELGYVLKGQTDKDFEKALFKLKDGEVSDVVKSSFGYHIIKADKPTDFNSEKQSLKEKLVDQKVQKNPKLLTDAYKDLLKEYDVDFKDRDIKSVVEDKILNPEKLKQGGAQGGQSGMSQ

>HDP2932565.1 peptidylprolyl isomerase [Staphylococcus aureus]

MKMINKLIVPVTASALLLGACGASATDSKENTLISSKAGDVTVADTMKKIGKDQIANASFTEMLNKILADKYKNKVNDKKIDEQIEKMQKQYGGKDKFEKALQQQGLTADKYKENLRTAAYHKELLSDKIKISDSEIKEDSKKASHILIKVKSKKSDKEGLDDKEAKQKAEEIQKEVSKDPSKFGEIAKKESMDTGSTKKDGELGYVLKGQTDKDFEKALFKLKDGEVSDVVKSSFGYHIIKADKPTDFNSEKQSLKEKLVDQKVQKNPKLLTDAYKDLLKEYDVDFKDRDIKSVVEDKILNPEKLKQGGAQGGQSGMSQ

>HDT7014290.1 peptidylprolyl isomerase [Staphylococcus aureus]

MKMINKLIVPVTASALLLGACGASATDSKENTLISSKAGDVTVADTMKKIGKDQIANASFTEMLNKILADKYKNKVNDKKIDEQIEKMQKQYGGKDKFEKALQQQGLTADKYKENLRTAAYHKELLSDKIKISDSEIKEDSKKASHILIKVKSKKSDKEGLDDKEAKQKAEEIQKEVSKDPSKFGEIAKKESMDTGSAKKDGELGYVLKGQTDKDFEKALFKLKDGEVSDVVKSSFGYHIIKADKPTDFNSEKQSLKEKLVDQKVQKNPKLLTDAYKDLLKEYDVDFKDRDIKSVVEDKILNPEKLKQGGAQGEQSGMSQ

>HDD7669986.1 peptidylprolyl isomerase [Staphylococcus aureus]

MKMINKLIVPVTASALLLGACGASATDSKENTLISSKAGDVTVADTMKKIGKDQIANASFTEMLNKILADKYKNKVNDKKIDEQIEKMQKQYGGKDKFEKALQQQGLTADKYKENLRTAAYHKELLSDKIKISDSEIKEDSKKASHILIKVKSKKSDKEGLDDKEAKQKAEEIQKEVSKDPSKFGEIAKKESMDTGSAKKDGELGYVLKGQTDKDFEKALFKLKDGEVSDVVKSSFGYHIIKADKPTDFNSEKQSLKEKLVDQKVQKNPKLLTDAYKDLLKEYDVDFKDRDIKSVVEDKILNPEKLKPGGAQGGQSGMSQ

>WP_259380410.1 foldase protein PrsA [Staphylococcus aureus]

MKMINKLIVPVTASALLLGACGASATDSKENTLISSKAGDVTVADTMKKIGKDQIANASFTEMLNKILADKYKNKVNDKKIDEQIEKMQKQYGGKDKFEKALQQQGLTADKYKENLRTAAYHKELLSDKIKISDSEIKEDSKKASHILIKVKSKKSDKEGLDDKEAKQKAEEIQKEVSKDPSKFGEIAKKESMDTGSAKKDGELGYVLKGQTDKDFEKALFKLKDGEVSDVVKSSFGYHIIKADKPTDFNSEKQSLKEKLVDQKVQKNPKLLTDAYKDLLKEYDVDFKDRDIKSVVEDKILNPEKLKQGAAQGGQSGMSQ

>HCW7349144.1 peptidylprolyl isomerase [Staphylococcus aureus]

MKMINKLIVPVTASALLLGACGASATDSKENTLISSKAGDVTVADTMKKIGKDQIANASFTEMLNKILADKYKNKVNDKKIDEQIEKMQKQYGGKDKFEKALQQQGLTADKYKENLRTAAYHKELLSDKIKISDSEIKEDSKKASHILIKVKSKKSDKEGLDDKEAKQKAEEIQKEVSKDPSKFGEIAKKESMDTGSAKKDGELGYVLKGQTDKDFEKALFKLKDGEVSDVVKSSFGYHIIKADKPTDFNSEKQSLKEKLVDQKVQKNPKLLTDAYKDLLKEYDVDFKDRDIKSVVEDKILNPEKLKQGGAQAGQSGMSQ

>CAC5481888.1 Foldase protein PrsA precursor [Staphylococcus aureus]

MKMINKLIVPVTASALLLGACGASATDSKENTLISSKAGDVTVADTMKKIGKDQIANASFTEMLNKILADKYKNKVNDKKIDEQIEKMQKQYGGKDKFEKALQQQGLTADKYKENLRTAAYHKELLSDKIKISDSEIKEDSKKASHILIKVKSKKSDKEGLDDKEAKQKAEEIQKEVSKDPSKFGEIAKKESMDTGSAKKDGELGYVLKGQTDKDFEKALFKLKDGEVSDVVKSSFGYHIIKADKPTDFNSEKQSLKEKLVDHKVQKNPKLLTDAYKDLLKEYDVDFKDRDIKSVVEDKILNPEKLKQGGAQGGQSGMSQ

>NFZ49855.1 peptidylprolyl isomerase [Staphylococcus aureus]

MKMINKLIVPVTASALLLGACGASATDSKENTLISSKAGDVTVADTMKKIGKDQIANASFTEMLNKILADKYKNKVNDKKIDEQIEKMQKQYGGKDKFEKALQQQGLTADKYKENLRTAAYHKELLSDKIKISDSEIKEDSKKASHILIKVKSKKSDKEGLDDKEAKQKAEEIQKEVSKDPSKFGEIAKKESMDTGSAKKDGELGYVLKGQTDKDFEKALFKLKDGEVSDVVKSSFGYHIIKADKPTDFNSEKQSLKEKLVDQKVQRNPKLLTDAYKDLLKEYDVDFKDRDIKSVVEDKILNPEKLKQGGAKGGQSGMSQ

>HCZ1018804.1 peptidylprolyl isomerase [Staphylococcus aureus]

MKMINKLIVPVTASALLLGACGASATDSKENALISSKAGDVTVADTMKKIGKDQIANASFTEMLNKILADKYKNKVNDKKIDEQIEKMQKQYGGKDKFEKALQQQGLTADKYKENLRTAAYHKELLSDKIKISDSEIKEDSKKASHILIKVKSKKSDKEGLDDKEAKQKAEEIQKEVSKDPSKFGEIAKKESMDTGSAKKDGELGYVLKGQTDKDFEKALFKLKDGEVSDVVKSSFGYHIIKADKPTDFNSEKQSLKEKLVDQKVQKNPKLLTDAYKDLLKEYDVDFKDRDIKSVVEDKILNPEKLKQGGAQGGQSGMSQ

>HDJ7391155.1 peptidylprolyl isomerase [Staphylococcus aureus]

MKMINKLIVPVTASALLLGACGASATDSKENTLISSKAGDVTVADTMKKIGKDQIANASFTEMLNKILADKYKNKVNDKKIDEQIEKMQKQYGGKDKFEKALQQQGLTADKYKENLRTAAYHKELLSDKIKISDSEIKEDSKKASHIFIKVKSKKSDKEGLDDKEAKQKAEEIQKEVSKDPSKFGEIAKKESMDTGSAKKDGELGYVLKGQTDKDFEKALFKLKDGEVSDVVKSSFGYHIIKADKPTDFNSEKQSLKEKLVDQKVQKNPKLLTDAYKDLLKEYDVDFKDRDIKSVVEDKILNPEKLKQGGAQGGQSGMSQ

>HCQ2190203.1 peptidylprolyl isomerase [Staphylococcus aureus]

MKMINKLIVPVTASALLLGACGASATDSKENTLISSKAGDVTVADTIKKIGKDQIANASFTEMLNKILADKYKNKVNDKKIDEQIEKMQKQYGGKDKFEKALQQQGLTADKYKENLRTAAYHKELLSDKIKISDSEIKEDSKKASHILIKVKSKKSDKEGLDDKEAKQKAEEIQKEVSKDPSKFGEIAKKESMDTGSAKKDGELGYVLKGQTDKDFEKALFKLKDGEVSDVVKSSFGYHIIKADKPTDFNSEKQSLKEKLVDQKVQKNPKLLTDAYKDLLKEYDVDFKDRDIKSVVEDKILNPEKLKQGGAQGGQSGMSQ

>HCZ7799482.1 peptidylprolyl isomerase [Staphylococcus aureus]

MKMINKLIVPVTASALLLGACGASATDSKENTLISSKAGDVTVADTMKKIGKDQIANASFTEMLNKILADKYKNKVNDKKIDEQIEKMQKQYGGKDKFEKALQQQGLTADKYKENLRTAAYHKELLSDKIKISDSEIKEDSKKASHILIKVKSKKSDKEGLDDKEAKQKAEEIQKEVSKDPSKFGEIAKKESMDTGSAKKDGELGYVLKGQTDKDFEKALFKLKDGEVSDVVKSSFGYHIIKADKPTDFNSEKQSLKEKLIDQKVQKNPKLLTDAYKDLLKEYDVDFKDRDIKSVVEDKILNPEKLKQGGAQGGQSGMSQ

>WP_279760164.1 foldase protein PrsA [Staphylococcus aureus]

MKMINKLIVPVIASALLLGACGASATDSKENTLISSKAGDVTVADTMKKIGKDQIANASFTEMLNKILADKYKNKVNDKKIDEQIEKMQKQYGGKDKFEKALQQQGLTADKYKENLRTAAYHKELLSDKIKISDSEIKEDSKKASHILIKVKSKKSDKEGLDDKEAKQKAEEIQKEVSKDPSKFGEIAKKESMDTGSAKKDGELGYVLKGQTDKDFEKALFKLKDGEVSDVVKSSFGYHIIKADKPTDFNSEKQSLKEKLVDQKVQKNPKLLTDAYKDLLKEYDVDFKDRDIKSVVEDKILNPEKLKQGGAQGGQSGMSQ

>HAR4499086.1 peptidylprolyl isomerase [Staphylococcus aureus]

MKMINKLIVPVTASALLLGACGASATDSKENTLISSKAGDVTVADTMKKIGKDQIANASFTEMLNKILADKYKNKVNDKKIDEQIEKMQKQYGGKDKFEKALQQQGLTADKYKENLRTAAYHKELLSDKIKISDSEIKEDSKKASHILIKVKSKKSDKEGLDDKEAKQKAEEIQKEVSKDPSKFGEIAKKESMDTGSAKKDGELGYVLKGQTDKDFEKALFKLKDGEVSDVVKSSFGYHIIKADKPTDFNSEKQSLKEKLVDQKVQKNPKLLTDAYKDLLKEYDVDFKDRDIKSVVEDKILNPEKLKQDGAQGGQSGMSQ

>HCY0907600.1 peptidylprolyl isomerase [Staphylococcus aureus]

MKMINKLIVPVTASALLLGACGASATDSKENTLISSKAGDVTVADTMKKIGKDQIANASFTEMLNKILADKYKNKVNDKKIDEQIEKMQKQYGGKDKFEKALQQQGLTADKYKENLRTAAYHKELLSDKIKISDSEIKEDSKKASHILIKVKSKKSDKEGLDDKEAKQKAEEIQKEVSKDPSKFGEIAKKESMDTGSAKKDGELGYVLKGQTDKDFEKALFKLKDGEVSDVVKSSFGYHIIKADKPTDFNSEKQSLKEKLVDQKVQKNPKLLTDAYKDLLKEYDVDFKDRDIKSVVEDKILNPEKLKQGGAQGGQSDMSQ

>WP_061390690.1 foldase protein PrsA [Staphylococcus aureus]

MKTINKLIVPVTASALLLGACGASATDSKENTLISSKAGDVTVADTMKKIGKDQIANASFTEMLNKILADKYKNKVNDKKIDEQIEKMQKQYGGKDKFEKALQQQGLTADKYKENLRTAAYHKELLSDKIKISDSEIKEDSKKASHILIKVKSKKSDKEGLDDKEAKQKAEEIQKEVSKDPSKFGEIAKKESMDTGSAKKDGELGYVLKGQTDKDFEKALFKLKDGEVSDVVKSSFGYHIIKADKPTDFNSEKQSLKEKLVDQKVQKNPKLLTDAYKDLLKEYDVDFKDRDIKSVVEDKILNPEKLKQGGAQGGQSGMSQ

>EJN0198329.1 peptidylprolyl isomerase [Staphylococcus aureus]

MKMINKLIVPVTASALLLGACGASATDSKENTLISSKAGDVTVADTMKKIGKDQIANASFTEMLNKILADKYKNKVNDKKIDEQIEKMQKQYGGKDKFEKALQQQGLTADKYKENLRTAAYHKELLSDKIKISDSEIKEDSKKASHILIKVKSKKSDKEGLDDKEAKQKAEEIQKEVSKDPSKFGEIAKKESMDTGSAKKDGELGYVLKGQTDKDFEKALFKLKDGEVSDVVKSSFGYHIIKADKPTDFNSEKQSLKEKLVDQKVQKNPKLLTDAYKDLLKEYDVDFKDRDIKSVVEDKILNPEKLKQGGAQG

>HCX2218853.1 peptidylprolyl isomerase [Staphylococcus aureus]

MKMINKLIVPVTASALLLGACGASATDSKENTLISSKAGDVTVADTMKKIGKDQIANASFTEMLNKILADKYKNKVNDKKIDEQIEKMQKQYGGKDKFEKALQQQGLTADKYKENLRTAAYHKELLSDKIKISDSEIKKDSKKASHILIKVKSKKSDKEGLDDKEAKQKAEEIQKEVSKDPSKFGEIAKKESMDTGSAKKDGELGYVLKGQTDKDFEKALFKLKDGEVSDVVKSSFGYHIIKADKPTDFNSEKQSLKEKLVDQKVQKNPKLLTDAYKDLLKEYDVDFKDRDIKSVVEDKILNPEKLKQGGAKGGQSGMSQ

>HCZ0778451.1 peptidylprolyl isomerase [Staphylococcus aureus]

MKMINKLIVPVTASALLLGACGASATDSKENTLISSKAGDVTVADTVKKIGKDQIANASFTEMLNKILADKYKNKVNDKKIDEQIEKMQKQYGGKDKFEKALQQQGLTADKYKENLRTAAYHKELLSDKIKISDSEIKEDSKKASHILIKVKSKKSDKEGLDDKEAKQKAEEIQKEVSKDPSKFGEIAKKESMDTGSAKKDGELGYVLKGQTDKDFEKALFKLKDGEVSDVVKSSFGYHIIKADKPTDFNSEKQSLKEKLVDQKVQKNPKLLTDAYKDLLKEYDVDFKDRDIKSVVEDKILNPEKLKQGGAQGGQSGMSQ

>CAC5968450.1 Foldase protein PrsA precursor [Staphylococcus aureus]

MKMINKLIVPVTASALLLGACGASATDSKENTLISSKSGDVTVADTMKKIGKDQIANASFTEMLNKILADKYKNKVNDKKIDEQIEKMQKQYGGKDKFEKALQQQGLTADKYKENLRTAAYHKELLSDKIKISDSEIKEDSKKASHILIKVKSKKSDKEGLDDKEAKQKAEEIQKEVSKDPSKFGEIAKKESMDTGSAKKDGELGYVLKGQTDKDFEKALFKLKDGEVSDVVKSSFGYHIIKADKPTDFNSEKQSLKEKLVDQKVQKNPKLLTDAYKDLLKEYDVDFKDRDIKSVVEDKILNPEKLKQGGAQGGQSGMSQ

>WP_061730622.1 foldase protein PrsA [Staphylococcus aureus]

MKMINKLIVPVTASALLLGACGASATDSKENTLISSKAGDVTVADTMKKIGKDQIANASFTEMLNKILADKYKNKVNDKKIDEQIEKMQKQYGGKDKFEKALQQQGLTADKYKENLRTAAYHKELLSDKIKISDSEIKEDSKKASHILIKVKSKKNDKEGLDDKEAKQKAEEIQKEVSKDPSKFGEIAKKESMDTGSAKKDGELGYVLKGQTDKDFEKALFKLKDGEVSDVVKSSFGYHIIKADKPTDFNSEKQSLKEKLVDQKVQKNPKLLTDAYKDLLKEYDVDFKDRDIKSVVEDKILNPEKLKQGGAQGGQSGMSQ

>WP_070030914.1 foldase protein PrsA [Staphylococcus aureus]

MKMINKLIVPVTASALLLGACGASATDSKENTLISSKAGDVTVADTMKKIGKDQIANASFTEMLNKILADKYKNKVNDKKIDEQIEKMQKQYGGKDKFEKALQQQGLTADKYKENLRTAAYHKELLTDKIKISDSEIKEDSKKASHILIKVKSKKSDKEGLDDKEAKQKAEEIQKEVSKDPSKFGEIAKKESMDTGSAKKDGELGYVLKGQTDKDFEKALFKLKDGEVSDVVKSSFGYHIIKADKPTDFNSEKQSLKEKLVDQKVQKNPKLLTDAYKDLLKEYDVDFKDRDIKSVVEDKILNPEKLKQGGAQGGQSGMSQ

>HDJ2889598.1 peptidylprolyl isomerase [Staphylococcus aureus]

MKMINKLIVPVTASALLLGACGASATDSKENTLISSKAGDVTVADTMKKIGKDQIANASFTEMLNKILADKYKNKVNDKKIDEQIEKMQKQYGGKDKFEKALQQQGLTADKYKENLRTAAYHKELLSDKIKISDSEIKEDSKKASHILIKVKSKKSDKEGLDDKEAKQKAEEIQKEVSKDPSKFGEIAKKESMDTGSAKKDGELGYVLKGQTDKDFEKALFKLKDGEVSDVVKSSFGYHIIKADKPTDFNSEKQSLKEKLVNQKVQKNPKLLTDAYKDLLKEYDVDFKDRDIKSVVEDKILNPEKLKQGGAQGGQSGMSQ

>MDT4002345.1 peptidylprolyl isomerase [Staphylococcus aureus]

MKMINKLIVPVTASALLLGACGASATDSKENTLISSKAGDVTVADTMKKIGKDQIANASFTEMLNKILADKYKNKVNDKKIDEQIEKMQKQYGGKDKFEKALQQQGLTADKYKENLRTAAYHKELLSDKIKISDSEIKEDSKKASHILIKVKSKKSDKEGLDDKEAKQKAEEIQKEVAKDPSKFAEIAKKESMDTGSAKKDGELGYVLKGQTDKDFEKALFKLKDGEVSDVVKSSFGYHIIKADKPTDFNSE

>HDD2569701.1 peptidylprolyl isomerase [Staphylococcus aureus]

MKMINKLIVPVTASALLLGACGASATDSKENTLISSKAGDVTVADTMKKIGKDQIANASFTEMLNKILADKYKNKVNDKKIDEQIEKMQKQYGGKDKFEKALQQQGLTADKYKENLRTAAYHKELLSDKIKISDSEIKEDSKKASHILIKVKSKKSDKEGLDDKEAKQKAEEIQKEVSKDQSKFGEIAKKESMDTGSAKKDGELGYVLKGQTDKDFEKALFKLKDGEVSDVVKSSFGYHIIKADKPTDFNSEKQSLKEKLVDQKVQKNPKLLTDAYKDLLKEYDVDFKDRDIKSVVEDKILNPEKLKQGGAQGGQSGMSQ

>MDT4138693.1 peptidylprolyl isomerase [Staphylococcus aureus]

MKMINKLIVPVTASALLLGACGASATDSKENTLISSKAGDVTVADTMKKIGKDQIANASFTEMLNKILADKYKNKVNDKKIDEQIEKMQKQYGGKDKFEKALQQQGLTADKYKENLRTAAYHKELLSDKIKISDSEIKEDSKKASHILIKVKSKKSDKEGLDDKEAKQKAEEIQKEVSKDPSKFGEIAKKESMDTGSAKKDGELGYVLKGQTDKDFEKALFKLKDGEVSDVVKSSFGYHIIKADKPTDFNSEKQSLKEKLVDQKVQKNPKLLTDAYKDLLKEYDVDFKDRDIKSVVEDKILNPEKLKQGGAKGGQSGMS

>HDB1497142.1 peptidylprolyl isomerase [Staphylococcus aureus]

MKMINKLIVPVTASALLLGACGASATDSKENTLISSKAGDVTVADTMKKIGKDQIANASFTEMLNKILADKYKNKVNDKKIDEQIEKMQKQYGGKDKFEKALQQQGLTADKYKENLRTAAYHKELLSDKIKISDSEIKEDSKKASHILIKVKSKKSDKEGLDDKEAKQKAEEIQKEVSKDPSKFGEIAKKESMDTGSAKKDGELGYVLKGQTDKDFEKALFKLKDGEVSDVVKSSFGYHIIKADKPTDFNSEKQSLKEKLVDQKVQKNPKLLTDAYKDLLKEYDVDFKDRDIKSVVEDKILNPEKLKQGGAQGGQS

>HCC5631513.1 peptidylprolyl isomerase [Staphylococcus aureus]

MKMINKLIVPVTASALLLGACGASATDSKENTLISSKAGDVTVADTMKKIGKDQIANASFTEMLNKILADKYKNKVNDKKIDEQIEKMQKQYGGKDKFKKALQQQGLTADKYKENLRTAAYHKELLSDKIKISDSEIKEDSKKASHILIKVKSKKSDKEGLDDKEVKQKAEEIQKEVSKDPSKFGEIAKKESMDTGSAKKDGELGYVLKGQTDKDFEKALFKLKDGEVSDVVKSSFGYHIIKADKPTDFNSEKQSLKEKLVDQKVQKNPKLLTDAYKDLLKEYDVDFKDRDIKSVVEDKILNPEKLKQGGAQGGQSGMSQ

>HDF6489298.1 peptidylprolyl isomerase [Staphylococcus aureus]

MKMINKLIVPVTASALLLGACGASATDSKENTLISSKAGDVTVADTMKKIGKDQIANASFTEMLNKILADKYKNKVNDKKIDEQIEKMQKQYGGKDKFEKALQQQGLTADKYKENLRTAAYHKELLSDKIKISDSEIKEDSKKASHILIKVKSKKSDKEGLDDKEAKQKAEEIQKEVSKDPSKFGEVAKKESMDTGSAKKDGELGYVLKGQTDKDFEKALFKLKDGEVSDVVKSSFGYHIIKADKPTDFNSEKQSLKEKLVDQKVQKNPKLLTDAYKDLLKEYDVDFKDRDIKSVVEDKILNPEKLKQGGAQGGQSGMSQ

>HAR7586105.1 peptidylprolyl isomerase [Staphylococcus aureus]

MKMINKLIVPVTASALLLGACGASATDSKENTLISSKAGDVTVADTMKKIGKDQIANASFTEMLNKILADKYKNKVNDKKIDEQIEKMQKQYGGKDKFEKALQQQGLTADKYKENLRTAAYHKELLSDKIKISDSEIKEDSKKASHILIKVKSKKSDKEGLDDKEAKQKAEEIQKEVSKDPSKFGEIAKKESMDTGSAKKDGELGYVLKGQTDKDFEKALFKLKDGEVSDVVKSSFGYHIIKADKPTDFNSEKQSLKEKLVDQKVQKNPKLLTDAYKDLLKEYDVDFKDRDIKSVVEDKILNPEKLKQGGVKGGQSGMSQ

>HDI8234179.1 peptidylprolyl isomerase [Staphylococcus aureus]

MKMINKLIVPVTASALLLGACGAGATDSKENTLISSKAGDVTVADTMKKIGKDQIANASFTEMLNKILADKYKNKVNDKKIDEQIEKMQKQYGGKDKFEKALQQQGLTADKYKENLRTAAYHKELLSDKIKISDSEIKEDSKKASHILIKVKSKKSDKEGLDDKEAKQKAEEIQKEVSKDPSKFGEIAKKESMDTGSAKKDGELGYVLKGQTDKDFEKALFKLKDGEVSDVVKSSFGYHIIKADKPTDFNSEKQSLKEKLVDQKVQKNPKLLTDAYKDLLKEYDVDFKDRDIKSVVEDKILNPEKLKQGGAQGGQSGMSQ

>HAR4295870.1 peptidylprolyl isomerase [Staphylococcus aureus]

MKMINKLIVPVTASALLLGACGASATDSKENTLISSKAGDVTVADTMKKIGKDQIANASFTEMLNKILADKYKNKVNDKKIDEQIEKMQKQYGGKDKFEKALQQQGLTADKYKENLRTAAYHKELLSDKIKISDSEIKEDSKKASHILIKVKSKKSDKEGLDDKEAKQKAEEIQKEVSKDPSKFGEIAKKESMDTGSAKKDGELGYVLKGQTDKDFEKALFKLKDGEVSDVVKSSFGYHIIKADKPTDFNSEKQSLKEKLVDQKVQKNPKLLTDAYKDLLKEYDVDFKDRDIKSVVEDKILNPKKLKQGGAKGGQSGMSQ

>NGL50457.1 peptidylprolyl isomerase [Staphylococcus aureus]

MKIINKLIVPVTASALLLGACGASATDSKENTLISSKAGDVTVADTMKKIGKDQIANASFTEMLNKILADKYKNKVNDKKIDEQIEKMQKQYGGKDKFEKALQQQGLTADKYKENLRTAAYHKELLSDKIKISDSEIKEDSKKASHILIKVKSKKSDKEGLDDKEAKQKAEEIQKEVSKDPSKFGEIAKKESMDTGSAKKDGELGYVLKGQTDKDFEKALFKLKDGEVSDVVKSSFGYHIIKADKPTDFNSEKQSLKEKLVDQKVQKNPKLLTDAYKDLLKEYDVDFKDRDIKSVVEDKILNPEKLKQGGAQGGQSGMSQ

>HDB3161941.1 peptidylprolyl isomerase [Staphylococcus aureus]

MKMINKLIVPITASALLLGACGASATDSKENTLISSKAGDVTVADTMKKIGKDQIANASFTEMLNKILADKYKNKVNDKKIDEQIEKMQKQYGGKDKFEKALQQQGLTADKYKENLRTAAYHKELLSDKIKISDSEIKEDSKKASHILIKVKSKKSDKEGLDDKEAKQKAEEIQKEVSKDPSKFGEIAKKESMDTGSAKKDGELGYVLKGQTDKDFEKALFKLKDGEVSDVVKSSFGYHIIKADKPTDFNSEKQSLKEKLVDQKVQKNPKLLTDAYKDLLKEYDVDFKDRDIKSVVEDKILNPEKLKQGGAQGGQSGMSQ

>WP_000782118.1 MULTISPECIES: foldase protein PrsA [Staphylococcus]

MKMINKLIVPVTASALLLGACGASATDSKENTLISSKAGDVTVADTMKKIGKDQIANASFTEMLNKILADKYKNKVNDKKIDEQIEKMQKQYGGKDKFEKALQQQGLTADKYKENLRTAAYHKELLSDKIKISDSEIKEDSKKASHILIKVKSKKSDKEGLDDKEAKQKAEEIQKEVSKDPSKFGEIAKKESMDTGSAKKDGELGYVLKGQTDKDFEKALFKLKDGEVSDVVKSSFGYHIIKADKPTDFNSEKQSLKEKLVDQKVQKNPKLLTDAYKDLLKEYDVDFKDRDIKSVVEDKILNPEKLKQGGAKGGQSGMSQ

>HCT4622823.1 peptidylprolyl isomerase [Staphylococcus aureus]

MKMINKLIVPVTASALLLGACGASATDSKENTLISSKAGDVTVADTMKKIGKDQIANASFTEMLNKILADKYKNKVNDKKIDEQIEKMQKQYGGKDKFEKALQQQGLTADKYKENLRTAAYHKELLSDKIKISDSEIKEDSKKASHILIKVKSKKSDKEGLDDKEAKQKAEEIQKEVSKDPSKFGEIAKKESMDTGSAKKDGELGYVLKGQTDKDFEKALFKLKDGEVSDVVKSSFGYHIIKADKPTDFNSEKQSLKEKLVDQKVQKNPKLLTDAYKDLLKEYDVDFKDRDIKSVVEDKILNPEKLKQGGAKGGQSGM

>HDD0320126.1 peptidylprolyl isomerase [Staphylococcus aureus]

MKMINKLIVPLTASALLLGACGASATDSKENTLISSKAGDVTVADTMKKIGKDQIANASFTEMLNKILADKYKNKVNDKKIDEQIEKMQKQYGGKDKFEKALQQQGLTADKYKENLRTAAYHKELLSDKIKISDSEIKEDSKKASHILIKVKSKKSDKEGLDDKEAKQKAEEIQKEVSKDPSKFGEIAKKESMDTGSAKKDGELGYVLKGQTDKDFEKALFKLKDGEVSDVVKSSFGYHIIKADKPTDFNSEKQSLKEKLVDQKVQKNPKLLTDAYKDLLKEYDVDFKDRDIKSVVEDKILNPEKLKQGGAQGGQSGMSQ

>HCY8436409.1 peptidylprolyl isomerase [Staphylococcus aureus]

MKMINKLIVPVTASALLLGACGASATDSKENTLISSKAGDVTVADTMKKIGKDQIANASFTEMLNKILADKYKNKVNDKKIDEQIEKMQKQYGGKDKFEKALQQQGLTADKYKENLRTAAYHKELLSDKIKISDSEIKEDSKKASHILIKVKSKKSDKEGLDDKEAKQKAEEIQKEVSKDPSKFGEIAKKESMDTGSAKKDGELGYVLKGQTDKDFEKALFKLKDGEVSDVVKSSFGYHIIKADKPTDFNSEKQSLKEKLVDQKVQKNPKLLTDAYKDLLKEYDVDFKDRDIKSVAEDKILNPEKLKQGGAQGGQSGMSQ

>HCG2447696.1 peptidylprolyl isomerase [Staphylococcus aureus]

MKMINKLIVPVTASALLLGACGASATDSKENTLISSKAGDVTIADTMKKIGKDQIANASFTEMLNKILADKYKNKVNDKKIDEQIEKMQKQYGGKDKFEKALQQQGLTADKYKENLRTAAYHKELLSDKIKISDSEIKEDSKKASHILIKVKSKKSDKEGLDDKEAKQKAEEIQKEVSKDPSKFGEIAKKESMDTGSAKKDGELGYVLKGQTDKDFEKALFKLKDGEVSDVVKSSFGYHIIKADKPTDFNSEKQSLKEKLVDQKVQKNPKLLTDAYKDLLKEYDVDFKDRDIKSVVEDKILNPEKLKQGGAQGGQSGMSQ

>HDD4370087.1 peptidylprolyl isomerase [Staphylococcus aureus]

MKMINKLIVPVTASALLLGACGASATDSKENTLISSKAGDVTVADTMKKIGKDQIANASFTEMLNKILADKYKNKVNDKKIDEQIEKMQKQYGGKDKFEKALQQQGLTADKYKENLRTAAYHKELLSDKIKISDSEIKEDSKKASHILIKVKSKKSDKEGLDDKEAKQKAEEIQKEVSKDPSKFGEIAKKESMDTGSAKKDGELGYVLKGQTDKDFEKALFKLKDGEVSDIVKSSFGYHIIKADKPTDFNSEKQSLKEKLVDQKVQKNPKLLTDAYKDLLKEYDVDFKDRDIKSVVEDKILNPEKLKQGGAQGGQSGMSQ

>XAP97548.1 peptidylprolyl isomerase [Staphylococcus aureus]

MKMINKLIVPVTASALLLGACGASATDSKENTLISSKAGDVTVADTMKKIGKDQIANASFTEMLNKILADKYKNKVNDKKIDEQIEKMQKQYGGKDKFEKALQQQGLTADKYKENLRTVAYHKELLSDKIKISDSEIKEDSKKASHILIKVKSKKSDKEGLDDKEAKQKAEEIQKEVSKDPSKFGEIAKKESMDTGSAKKDGELGYVLKGQTDKDFEKALFKLKDGEVSDVVKSSFGYHIIKADKPTDFNSEKQSLKEKLVDQKVQKNPKLLTDAYKDLLKEYDVDFKDRDIKSVVEDKILNPEKLKQGGAQGGQSGMSQ

>HDG5360377.1 peptidylprolyl isomerase [Staphylococcus aureus]

MKMINKLIVPVTASALLLGACGASATDSKENTLISSKAGDVTVADTMKKIGKDQIANASFTEMLNKILVDKYKNKVNDKKIDEQIEKMQKQYGGKDKFEKALQQQGLTADKYKENLRTAAYHKELLSDKIKISDSEIKEDSKKASHILIKVKSKKSDKEGLDDKEAKQKAEEIQKEVSKDPSKFGEIAKKESMDTGSAKKDGELGYVLKGQTDKDFEKALFKLKDGEVSDVVKSSFGYHIIKADKPTDFNSEKQSLKEKLVDQKVQKNPKLLTDAYKDLLKEYDVDFKDRDIKSVVEDKILNPEKLKQGGAQGGQSGMSQ

>HEC4214732.1 peptidylprolyl isomerase [Staphylococcus aureus]

MKMINKLIVPVTASALLLDACGASATDSKENTLISSKAGDVTVADTMKKIGKDQIANASFTEMLNKILADKYKNKVNDKKIDEQIEKMQKQYGGKDKFEKALQQQGLTADKYKENLRTAAYHKELLSDKIKISDSEIKEDSKKASHILIKVKSKKSDKEGLDDKEAKQKAEEIQKEVSKDPSKFGEIAKKESMDTGSAKKDGELGYVLKGQTDKDFEKALFKLKDGEVSDVVKSSFGYHIIKADKPTDFNSEKQSLKEKLVDQKVQKNPKLLTDAYKDLLKEYDVDFKDRDIKSVVEDKILNPEKLKQGGAQGGQSGMSQ

>HCY7460340.1 peptidylprolyl isomerase [Staphylococcus aureus]

MKMINKLIVPVTASALLLGACGASATDSKENTLISSKAGDVTVADTMKKIGKDQIANASFTEMLNKILADKYKNKVNDKKIDEQIEKMQKQYGGKDKFEKVLQQQGLTADKYKENLRTAAYHKELLSDKIKISDSEIKEDSKKASHILIKVKSKKSDKEGLDDKEAKQKAEEIQKEVSKDPSKFGEIAKKESMDTGSAKKDGELGYVLKGQTDKDFEKALFKLKDGEVSDVVKSSFGYHIIKADKPTDFNSEKQSLKEKLVDQKVQKNPKLLTDAYKDLLKEYDVDFKDRDIKSVVEDKILNPEKLKQGGAQGGQSGMSQ

>HCT9701539.1 peptidylprolyl isomerase [Staphylococcus aureus]

MKMINKLIVPVTASALLLGACGASATDSKENTLISSKAGDVTVADTMKKIGKDQIANASFTEMLNKILADKYKNKVNDKKIDEQIEKMQKQYGGKDKFEKALQQQGLTADKYKENLRTAAYHKELLSDKIKISDSEIKEDSKKASHILIKVKSKKSDKEGLDDKEAKQKAEEIQKEVSKDPSKFGEIAKKESMDTGSAKKDGELGYVLKGQTDKDFEKALFKLKDGEVSDVVKSSFGYHIIKADKPTDFNSEKQSLKEKLVDQKVQKNPKLLTDAYKDLLKEYDVDFKDRDIKSVVEDKILNSEKLKQGGAKGGQSGMSQ

>HDT6992939.1 peptidylprolyl isomerase [Staphylococcus aureus]

MKMINKLIVPVTASALLLGACGASATDSKENTLISSKAGDVTVADTMKKIGKDQIANASFTEMLNKILADKYKNKVNDKKIDEQIEKMQKQHGGKDKFEKALQQQGLTADKYKENLRTAAYHKELLSDKIKISDSEIKEDSKKASHILIKVKSKKSDKEGLDDKEAKQKAEEIQKEVSKDPSKFGEIAKKESMDTGSAKKDGELGYVLKGQTDKDFEKALFKLKDGEVSDVVKSSFGYHIIKADKPTDFNSEKQSLKEKLVDQKVQKNPKLLTDAYKDLLKEYDVDFKDRDIKSVVEDKILNPEKLKQGGAQGGQAGMSQ

>WP_226452240.1 foldase protein PrsA [Staphylococcus aureus]

MKMINKLIVPVTASALLLGACGASATDSKENTLISSKAGDVTVADTMKKIGKDQIANASFTEMLNKILADKYKNKVNDKKIDEQIEKMQKQYGGKDKFEKALQQQGLTADKYKENLRTAAYHKELLSDKIKISDSEIKEYSKKASHILIKVKSKKSDKEGLDDKEAKQKAEEIQKEVSKDPSKFGEIAKKESMDTGSAKKDGELGYVLKGQTDKDFEKALFKLKDGEVSDVVKSSFGYHIIKADKPTDFNSEKQSLKEKLVDQKVQKNPKLLTDAYKDLLKEYDVDFKDRDIKSVVEDKILNPEKLKQGGAKGGQSGMSQ

>HDH5613823.1 peptidylprolyl isomerase [Staphylococcus aureus]

MKMINKLIVPVTASALLLGACGASATDSKENTLISSKAGDVTVADTMKKIGKDQIANASFTEMLNKILADKYKNKVNDKKIDEQIEKMQKQYGGKDKFEKALQQQGLTADKYKENLRTAAYHKELLSDKIKISDSEIKEDSKKASHILIKVKSKKSDKEGLDDKEAKQKAEKIQKEVSKDPSKFGEIAKKESMDTGSAKKDGELGYVLKGQTDKDFEKALFKLKDGEVSDVVKSSFGYHIIKADKPTDFNSEKQSLKEKLVDQKVQKNPKLLTDAYKDLLKEYDVDFKDRDIKSVVEDKILNPEKLKQGGAQGGQSGMSQ

>WP_031918799.1 foldase protein PrsA [Staphylococcus aureus]

MKMINKLIVPVTASALLLGACGASATDSKENTLISSKAGDVTVADTMKKIGKDQIANASFTEMLNKILADKYKNKVNDKKIDEQIEKTQKQYGGKDKFEKALQQQGLTADKYKENLRTAAYHKELLSDKIKISDSEIKEDSKKASHILIKVKSKKSDKEGLDDKEAKQKAEEIQKEVSKDPSKFGEIAKKESMDTGSAKKDGELGYVLKGQTDKDFEKALFKLKDGEVSDVVKSSFGYHIIKADKPTDFNSEKQSLKEKLVDQKVQKNPKLLTDAYKDLLKEYDVDFKDRDIKSVVEDKILNPEKLKQGGAQGGQSGMSQ

>HDG5860829.1 peptidylprolyl isomerase [Staphylococcus aureus]

MKMINKLIVPVTASALLLGACGASATDSKENTLISSKAGDVTVADTMKKIGKDQIANASFTEMLNKILADKYKNKVNDKKIDEQIEKMQKQYGGKDKFEKALQQQGLTVDKYKENLRTAAYHKELLSDKIKISDSEIKEDSKKASHILIKVKSKKSDKEGLDDKEAKQKAEEIQKEVSKDPSKFGEIAKKESMDTGSAKKDGELGYVLKGQTDKDFEKALFKLKDGEVSDVVKSSFGYHIIKADKPTDFNSEKQSLKEKLVDQKVQKNPKLLTDAYKDLLKEYDVDFKDRDIKSVVEDKILNPEKLKQGGAQGGQSGMSQ

>HDI0883817.1 peptidylprolyl isomerase [Staphylococcus aureus]

MKMINKLIVPVTASALLLGAYGASATDSKENTLISSKAGDVTVADTMKKIGKDQIANASFTEMLNKILADKYKNKVNDKKIDEQIEKMQKQYGGKDKFEKALQQQGLTADKYKENLRTAAYHKELLSDKIKISDSEIKEDSKKASHILIKVKSKKSDKEGLDDKEAKQKAEEIQKEVSKDPSKFGEIAKKESMDTGSAKKDGELGYVLKGQTDKDFEKALFKLKDGEVSDVVKSSFGYHIIKADKPTDFNSEKQSLKEKLVDQKVQKNPKLLTDAYKDLLKEYDVDFKDRDIKSVVEDKILNPEKLKQGGAQGGQSGMSQ

>HDP6020992.1 peptidylprolyl isomerase [Staphylococcus aureus]

MKMINKLIVPVTASALLLGACGASATDSKENTLISSKAGDVTVADTMKKIGKDQIANASFTEMLNKILADKYKNKVNDKKIDEQIEKMQKQYGGKDKFEKALQQQGLTADKYKENLRTAAYRKELLSDKIKISDSEIKEDSKKASHILIKVKSKKSDKEGLDDKEAKQKAEEIQKEVSKDPSKFGEIAKKESMDTGSAKKDGELGYVLKGQTDKDFEKALFKLKDGEVSDVVKSSFGYHIIKADKPTDFNSEKQSLKEKLVDQKVQKNPKLLTDAYKDLLKEYDVDFKDRDIKSVVEDKILNPEKLKQGGAQGGQSGMSQ

>HDZ6019101.1 peptidylprolyl isomerase [Staphylococcus aureus]

MKMINKLIVPVTASALLLGACGASATDSKENTLISSKAGDVTVADIMKKIGKDQIANASFTEMLNKILADKYKNKVNDKKIDEQIEKMQKQYGGKDKFKKALQQQGLTADKYKENLRTAAYHKELLSDKIKISDSEIKEDSKKASHILIKVKSKKSDKEGLDDKEAKQKAEEIQKEVSKDPSKFGEIAKKESMDTGSAKKDGELGYVLKGQTDKDFEKALFKLKDGEVSDVVKSSFGYHIIKADKPTDFNSEKQSLKEKLVDQKVQKNPKLLTDAYKDLLKEYDVDFKDRDIKSVVEDKILNPEKLKQGGAQGGQSGMSQ

>WP_061389952.1 foldase protein PrsA [Staphylococcus aureus]

MKMINKLIVPVTASALLLGACGASATDSKENTLISSKAGDVTVADTMKKIGKDQIANASFTEMLNKILADKYKNKVNDKKIDEQIEKMQKQYGGKDKFEKALQQQGLTADKYKENLRTAAYHKELLSDKIKISDSEIKEDSKKASHILIKVKSKKSDKEGLDDKEAKQKAEEIQKEVSKDPSKFGEIAKKESMDTGSAKKDGELGYVLKGQTDKDFEKALFKLKDGEVSDVVKSSFGYHIIKADKPTDFNSEKQSLKEKLVDQKVQKNPKLLTDAYKDLLKEYDVDFKDRDIKSFVEDKILNPEKLKQGGAQGGQSGMSQ

>MBU5153209.1 peptidylprolyl isomerase [Staphylococcus aureus]

MKMINKLIVPVTASALLLGACGASATDSKENTLISSKAGDVTVADTMKKIGKDQIANASFTEMLNKILADKYKNKVNDKKIDEQIEKMQKQYGGKDKFKKALQQQGLTADKYKENLRTAAYHKELLSDKIKISDSEIKEDSKKASHILIKVKSKKSDKEGLEDKEAKQKAEEIQKEVSKDPSKFGEIAKKESMDTGSAKKDGELGYVLKGQTDKDFEKALFKLKDGEVSDVVKSSFGYHIIKADKPTDFNSEKQSLKEKLVDQKVQKNPKLLTDAYKDLLKEYDVDFKDRDIKSVVEDKILNPEKLKQGGAQGGQSGMSQ

>NGH74799.1 peptidylprolyl isomerase [Staphylococcus aureus]

MKMINKLIVPVTASALLLGACGASATDSKENTLISSKAGDVTVADTMKKIGKDQIANASFTEMLNKILADKYKNKVNDKKIDEQIERMQKQYGGKDKFEKALQQQGLTADKYKENLRTAAYHKELLSDKIKISDSEIKEDSKKASHILIKVKSKKSDKEGLDDKEAKQKAEEIQKEVSKDPSKFGEIAKKESMDTGSAKKDGELGYVLKGQTDKDFEKALFKLKDGEVSDVVKSSFGYHIIKADKPTDFNSEKQSLKEKLVDQKVQKNPKLLTDAYKDLLKEYDVDFKDRDIKSVVEDKILNPEKLKQGGAQGGQSGMSQ

>CAC9092495.1 Foldase protein PrsA precursor [Staphylococcus aureus]

MKMINKLIVPVTASALLLGACGASATDSKENTLISSKAGDVTVADTMKKIGKDQIANASFTEMLNKILADKYKNKVNDKKIDEQIEKMQKQYGGKDKFEKALQQQGLTADKYKENLRTAAYHKELLSDKIKISDSEIREDSKKASHILIKVKSKKSDKEGLDDKEAKQKAEEIQKEVSKDPSKFGEIAKKESMDTGSAKKDGELGYVLKGQTDKDFEKALFKLKDGEVSDVVKSSFGYHIIKADKPTDFNSEKQSLKEKLVDQKVQKNPKLLTDAYKDLLKEYDVDFKDRDIKSVVEDKILNPEKLKQGGAQGGQSGMSQ

>HDJ6465707.1 peptidylprolyl isomerase [Staphylococcus aureus]

MKMINKLIVPVTASALLLGACGASATDSKENTLISSKAGDVTVADTMKKIGKDQIANASFTEMLNKILADKYKNKVNDKKIDEQIEKMQKQYGGKDKFEKALQQQGLTADKYKENLRTAAYHKELLSDKIKISDSEIKEDSKKASHILIKVKSKKSDKEGLDDKEAKQKAEEIQKEVSKDPSKFGEIAKKESMDTGSAKKDGELGYVLKGQTDKDFEKALFKLKDGEVSDVVKSSFGYHIIKADKPTDFNSEKQSLKEKLVDQKVQKNPKLLTDAYKDLLKEYDVDFRDRDIKSVVEDKILNPEKLKQGGAQGGQSGMSQ

>WP_233665693.1 foldase protein PrsA [Staphylococcus aureus]

MKMINKLIVPVTASALLLGACGASATDSKENTLISSKAGDVTVADTMKKIGKDQIANASFTEMLNKILADKYKNKVNDKKIDEQIEKMQKQYGGKDKFEKALQQQGLTADKYKENLRTAAYHKELLSDKIKISDSEIKEDSKKASHILIKVKSKRSDKEGLDDKEAKQKAEEIQKEVSKDPSKFGEIAKKESMDTGSAKKDGELGYVLKGQTDKDFEKALFKLKDGEVSDVVKSSFGYHIIKADKPTDFNSEKQSLKEKLVDQKVQKNPKLLTDAYKDLLKEYDVDFKDRDIKSVVEDKILNPEKLKQGGAQGGQSGMSQ

>ELK6125794.1 peptidylprolyl isomerase [Staphylococcus aureus]

MKMINKLIVPVTASALLLGACGANATDSKENTLISSKAGDVTVADTMKKIGKDQIANASFTEMLNKILADKYKNKVNDKKIDEQIEKMQKQYGGKDKFEKALQQQGLTADKYKENLRTAAYHKELLSDKIKISDSEIKEDSKKASHILIKVKSKKSDKEGLDDKEAKQKAEEIQKEVSKDPSKFGEIAKKESMDTGSAKKDGELGYVLKGQTDKDFEKALFKLKDGEVSDVVKSSFGYHIIKADKPTDFNSEKQSLKEKLVDQKVQKNPKLLTDAYKDLLKEYDVDFKDRDIKSVVEDKILNPEKLKQGGAQGGQSGMSQ

>HDA2204579.1 peptidylprolyl isomerase [Staphylococcus aureus]

MKMINKLIVPVTASALLLGACGASATDSKENTLISSKAGDVTVADTMKKIGKDQIANASFTEMLNKILADKYKNKVNDKKIDEQIEKMQKQYGGKDKFEKALQQQGLTADKYKENLRTAAYHKELLSDKIKISDSEIKEDSKKASHILIKVKSKKSDKEGLDDKEAKQKAEEIQKEVSKDPSKFGEIAKKESMDTGSAKKDGELGYVLKGQTDKGFEKALFKLKDGEVSDVVKSSFGYHIIKADKPTDFNSEKQSLKEKLVDQKVQKNPKLLTDAYKDLLKEYDVDFKDRDIKSVVEDKILNPEKLKQGGAQGGQSGMSQ

>HDZ5880397.1 peptidylprolyl isomerase [Staphylococcus aureus]

MKMINKLIVPVTASALLLGACGASATDSKENTLISSKAGDVTVADTMKKIGKDQIANASFTEMLNKILADKYKNKVDDKKIDEQIEKMQKQYGGKDKFEKALQQQGLTADKYKENLRTAAYHKELLSDKIKISDSEIKEDSKKASHILIKVKSKKSDKEGLDDKEAKQKAEEIQKEVSKDPSKFGEIAKKESMDTGSAKKDGELGYVLKGQTDKDFEKALFKLKDGEVSDVVKSSFGYHIIKADKPTDFNSEKQSLKEKLVDQKVQKNPKLLTDAYKDLLKEYDVDFKDRDIKSVVEDKILNPEKLKQGGAQGGQSGMSQ

>HEI5848252.1 peptidylprolyl isomerase [Staphylococcus aureus]

MKMINKLIVPVTASALLLGACGASATDSKENTLISSKAGDVTVADTMKKIGKDQIANASFTEILNKILADKYKNKVNDKKIDEQIEKMQKQYGGKDKFKKALQQQGLTADKYKENLRTAAYHKELLSDKIKISDSEIKEDSKKASHILIKVKSKKSDKEGLDDKEAKQKAEEIQKEVSKDPSKFGEIAKKESMDTGSAKKDGELGYVLKGQTDKDFEKALFKLKDGEVSDVVKSSFGYHIIKADKPTDFNSEKQSLKEKLVDQKVQKNPKLLTDAYKDLLKEYDVDFKDRDIKSVVEDKILNPEKLKQGGAQGGQSGMSQ

>HDC3974027.1 peptidylprolyl isomerase [Staphylococcus aureus]

MKMINKLIVPVTASALLLGACGASATDSKENTLISSKAGDVTVADTMKKIGKDQIANASFTEMLNKILADKYKNKVNDKKIDEQIEKMQKQYGGKDKFEKALQQQGLTADKYKENLRTAAYHKELLSDKIKISDSEIKEDSKKASHILIKVKSKKSDKEGLDDKEAKQKAGEIQKEVSKDPSKFGEIAKKESMDTGSAKKDGELGYVLKGQTDKDFEKALFKLKDGEVSDVVKSSFGYHIIKADKPTDFNSEKQSLKEKLVDQKVQKNPKLLTDAYKDLLKEYDVDFKDRDIKSVVEDKILNPEKLKQGGAQGGQSGMSQ

>WP_061641475.1 foldase protein PrsA [Staphylococcus aureus]

MKMINKLIVPVTASALLLGACGASATDSKENTLISSKAGDVTVADTMKKIGKDQIANASFTEMLNKILADKYKNKVNDKKIDEQIEKMQKQYGGKDKFEKALQQQGLTADKYKENLRTAAYHKELLSDKIKISDSEIKEDSKKASHILIKVKSKKSDKEGLDDKEAKQKAEEIQKEVSKDPSKFGEIAKKESMDTGSAKKDGELGYVLKGQTDKDFEKALFKLKDGEVSDVVKSSFGYHIIKADKPTDFNSEKQSLKEKLVDQKVQKNPKLLTDSYKDLLKEYDVDFKDRDIKSVVEDKILNPEKLKQGGAQGGQSGMSQ

>HCV3590592.1 peptidylprolyl isomerase [Staphylococcus aureus]

MKMINKLIVPVTASALLLGACGASATDSKENTLISSKAGDVTVSDTMKKIGKDQIANASFTEMLNKILADKYKNKVNDKKIDEQIEKMQKQYGGKDKFKKALQQQGLTADKYKENLRTAAYHKELLSDKIKISDSEIKEDSKKASHILIKVKSKKSDKEGLDDKEAKQKAEEIQKEVSKDPSKFGEIAKKESMDTGSAKKDGELGYVLKGQTDKDFEKALFKLKDGEVSDVVKSSFGYHIIKADKPTDFNSEKQSLKEKLVDQKVQKNPKLLTDAYKDLLKEYDVDFKDRDIKSVVEDKILNPEKLKQGGAQGGQSGMSQ

>WP_123138779.1 foldase protein PrsA [Staphylococcus aureus]

MKMINKLIVPVTASALLLGACGASATDSKENTLISSKAGDVTVADTMKKIGKDQIANASFTEMLNKILADKYKNKVNDKKIDEQIEKMQKQYGGKDKFEKALQQQGLTADKYKENLRTAAYHKELLSDKIKISDSEIKEDSKKASHILIKVKSKKSDKQGLDDKEAKQKAEEIQKEVSKDPSKFGEIAKKESMDTGSAKKDGELGYVLKGQTDKDFEKALFKLKDGEVSDVVKSSFGYHIIKADKPTDFNSEKQSLKEKLVDQKVQKNPKLLTDAYKDLLKEYDVDFKDRDIKSVVEDKILNPEKLKQGGAQGGQSGMSQ

>HCT1231448.1 peptidylprolyl isomerase [Staphylococcus aureus]

MKMINKLIVPVTASALLLGACGASATDSKENTLISSKAGDVTVADTMKKIGKDQIANASFTEMLNKILADKYKNKVNDKKIDEQIEKMQKQYGGKDKFEKALQQQGLTADKYKENLRTAAYHKELLSDKIKISDSEIKEDSKKASHILIKVKSKKSDKEGLDDKEAKQKAEEIQKEVSKDPSKFGEIAKKESMDTGSAKKDGELGYVLKGQTDKDFEKALFKLKDGEVSDVVKSSFGYHIIKADKPTDFNIEKQSLKEKLVDQKVQKNPKLLTDAYKDLLKEYDVDFKDRDIKSVVEDKILNPEKLKQGGAQGGQSGMSQ

>HCV9028775.1 peptidylprolyl isomerase [Staphylococcus aureus]

MKMINKLIVPVTASALLLGACGASATDSKGNTLISSKAGDVTVADTMKKIGKDQIANASFTEMLNKILADKYKNKVNDKKIDEQIEKMQKQYGGKDKFEKALQQQGLTADKYKENLRTAAYHKELLSDKIKISDSEIKEDSKKASHILIKVKSKKSDKEGLDDKEAKQKAEEIQKEVSKDPSKFGEIAKKESMDTGSAKKDGELGYVLKGQTDKDFEKALFKLKDGEVSDVVKSSFGYHIIKADKPTDFNSEKQSLKEKLVDQKVQKNPKLLTDAYKDLLKEYDVDFKDRDIKSVVEDKILNPEKLKQGGAQGGQSGMSQ

>HCU0646515.1 peptidylprolyl isomerase [Staphylococcus aureus]

MKMINKLIVPVTASALLLGACGASATDSKENTLISSKAGDVTVADTMKKIGKDQIANASFTEMLNKILADKYKNKVNDKKIDEQIEKMQKQYGGKDKFEKALQQQGLTADKYKENLRTAAYHKELLSDKIKISDSEIKEDSKKASHILIKVKSKKSDKEGLDDKEAKQKAEEIQKEVSKDPSKFGGIAKKESMDTGSAKKDGELGYVLKGQTDKDFEKALFKLKDGEVSDVVKSSFGYHIIKADKPTDFNSEKQSLKEKLVDQKVQKNPKLLTDAYKDLLKEYDVDFKDRDIKSVVEDKILNPEKLKQGGAQGGQSGMSQ

>WP_031878995.1 foldase protein PrsA [Staphylococcus aureus]

MKMINKLIVPVTASALLLGACGASATDSKENTLISSKAGDVTVADTMKKIGKDQIANASFTEMLNKILADKYKNKVNDKKIDEQIEKMQKQYGGKDKFKKALQQQGLTADKYKENLRTAAYHKELLSDKIKISDSEIKEDSKKASHILIKVKSKKSDKEGLDDKEAKQKAEEIQKEVSKDPSKFGEIAKKESMDTDSAKKDGELGYVLKGQTDKDFEKALFKLKDGEVSDVVKSSFGYHIIKADKPTDFNSEKQSLKEKLVDQKVQKNPKLLTDAYKDLLKEYDVDFKDRDIKSVVEDKILNPEKLKQGGAQGGQSGMSQ

>HDB5574426.1 peptidylprolyl isomerase [Staphylococcus aureus]

MKMINKLIVPVTASALLLGACGASATDSKENTLISSKAGDVTVADTMKKIGKDQIANASFTEMLNKILADKYKNKVNDKKIDEQIEKMQKQYGGKDKFEKALQQQGLTADKYKENLRTAAYHKELLSDKIKISDSEIKEDSKKASHILIKVKSKKSDKEGLDDKEAKQKAEEIQKEVSKDPSKFGEIAKKDSMDTGSAKKDGELGYVLKGQTDKDFEKALFKLKDGEVSDVVKSSFGYHIIKADKPTDFNSEKQSLKEKLVDQKVQKNPKLLTDAYKDLLKEYDVDFKDRDIKSVVEDKILNPEKLKQGGAQGGQSGMSQ

>EKF1475486.1 peptidylprolyl isomerase [Staphylococcus aureus]

MKMINKLIVPVTASALLLGACGASATDSKENTLISSKTGDVTVADTMKKIGKDQIANASFTEMLNKILADKYKNKVNDKKIDEQIEKMQKQYGGKDKFEKALQQQGLTADKYKENLRTAAYHKELLSDKIKISDSEIKEDSKKASHILIKVKSKKSDKEGLDDKEAKQKAEEIQKEVSKDPSKFGEIAKKESMDTGSAKKDGELGYVLKGQTDKDFEKALFKLKDGEVSDVVKSSFGYHIIKADKPTDFNSEKQSLKEKLVDQKVQKNPKLLTDAYKDLLKEYDVDFKDRDIKSVVEDKILNPEKLKQGGAQGGQSGMSQ

>HDI7459877.1 peptidylprolyl isomerase [Staphylococcus aureus]

MKMINKLIVPVTASALLLGACGASATDSKENTLISSKAGDVTVADTMKKIGKDQIANASFTEMLNKILADKYKNKVNDKKIDEQIEKMQKQYGGKDKFEKALQQQGLTADKYKENLRTAAYHKELLSDKIKISDSEIKEDNKKASHILIKVKSKKSDKEGLDDKEAKQKAEEIQKEVSKDPSKFGEIAKKESMDTGSAKKDGELGYVLKGQTDKDFEKALFKLKDGEVSDVVKSSFGYHIIKADKPTDFNSEKQSLKEKLVDQKVQKNPKLLTDAYKDLLKEYDVDFKDRDIKSVVEDKILNPEKLKQGGAQGGQSGMSQ

>WP_111065313.1 foldase protein PrsA [Staphylococcus aureus]

MKMINKLIVPVTASALLLGACGASATDSKENTLISSKAGDVTVADTMKKIGKDQIANASFTEMLNKILADKYKNKVNDKKIDEQIEKMQKQYGGKDKFEKALQQQGLTADKYKENLRTAAYHKELLSDKIKISDFEIKEDSKKASHILIKVKSKKSDKEGLDDKEAKQKAEEIQKEVSKDPSKFGEIAKKESMDTGSAKKDGELGYVLKGQTDKDFEKALFKLKDGEVSDVVKSSFGYHIIKADKPTDFNSEKQSLKEKLVDQKVQKNPKLLTDAYKDLLKEYDVDFKDRDIKSVVEDKILNPEKLKQGGAQGGQSGMSQ

>WP_111079786.1 foldase protein PrsA [Staphylococcus aureus]

MKMINKLIVPVTASALLLGACGASATDSKENTLISSKAGDVTVADTMKKIGKDQIANASFTEMLNKILADKYKNKVNDKKIDEQIEKMQKQYGGKDKFEKALQQQGLTADKYKENLRTAAYHKELLSDKIKISDSEIKEDSKKASHILIKVKSKKSDKEGLDDKEAKQKAEEIQKEVSKDPSKFGEIAKKESMDTGSAKKDGELGYVLKGQTDKDFEKALFKLKDGEVSDVVKSSFGYHIIKADKPTDFNSEKQSLKKKLVDQKVQKNPKLLTDAYKDLLKEYDVDFKDRDIKSVVEDKILNPEKLKQGGAQGGQSGMSQ

>WP_033858294.1 foldase protein PrsA [Staphylococcus aureus]

MKMINKLIVPVTASALLLGACGASATDSKENTLISSKAGDVTVADTMKKIGKDQIANASFTEMLNKILADKYKNKVNDKKIDEQIKKMQKQYGGKDKFEKALQQQGLTADKYKENLRTAAYHKELLSDKIKISDSEIKEDSKKASHILIKVKSKKSDKEGLDDKEAKQKAEEIQKEVSKDPSKFGEIAKKESMDTGSAKKDGELGYVLKGQTDKDFEKALFKLKDGEVSDVVKSSFGYHIIKADKPTDFNSEKQSLKEKLVDQKVQKNPKLLTDAYKDLLKEYDVDFKDRDIKSVVEDKILNPEKLKQGGAQGGQSGMSQ

>WP_222637413.1 foldase protein PrsA [Staphylococcus aureus]

MKMINKLIVPVTASALLLGACGASATDSKENTLISSKAGDVTVADTMKKIGKDQIANASFTEMLNKILADKYKNKVNDKKIDEQIEKMQKQYGGKDKFEKALQQQGLTADKYKENLRTAAYHKELLSDKIKISDSKIKEDSKKASHILIKVKSKKSDKEGLDDKEAKQKAEEIQKEVSKDPSKFGEIAKKESMDTGSAKKDGELGYVLKGQTDKDFEKALFKLKDGEVSDVVKSSFGYHIIKADKPTDFNSEKQSLKEKLVDQKVQKNPKLLTDAYKDLLKEYDVDFKDRDIKSVVEDKILNPEKLKQGGAQGGQSGMSQ

>ORO18390.1 peptidylprolyl isomerase [Staphylococcus aureus]

MKMINKLIVPVTASALLLGACGASVTDSKENTLISSKAGDVTVADTMKKIGKDQIANASFTEMLNKILADKYKNKVNDKKIDEQIEKMQKQYGGKDKFEKALQQQGLTADKYKENLRTAAYHKELLSDKIKISDSEIKEDSKKASHILIKVKSKKSDKEGLDDKEAKQKAEEIQKEVSKDPSKFGEIAKKESMDTGSAKKDGELGYVLKGQTDKDFEKALFKLKDGEVSDVVKSSFGYHIIKADKPTDFNSEKQSLKEKLVDQKVQKNPKLLTDAYKDLLKEYDVDFKDRDIKSVVEDKILNPEKLKQGGAQGGQSGMSQ

>HAR7072682.1 peptidylprolyl isomerase [Staphylococcus aureus]

MKMINKLIVPVTASALLLGACGASATDSKENTLISSKAGDVTVADTMKKIGKDQIANASFTEMLNKILADKYKNKVNDKKIDEQIEKMQKQYGGKDKFEKALQQQGLTADKYKENLRTAAYHKELMADKIKISDSEIKEDSKKASHILIKVKSKKSDKEGLDDKEAKQKAEEIQKEVSKDPSKFGEIAKKESMDTGSAKKDGELGYVLKGQTDKDFEKALFKLKDGEVSDVVKSSFGYHIIKADKPTDFNSEKQSLKEKLVDQKVQKNPKLLTDAYKDLLKEYDVDFKDRDIKSVVEDKILNPEKLKQGGAQGGQSGMSQ

>WP_149558572.1 foldase protein PrsA [Staphylococcus aureus]

MKMINKLIVPVTASALLLGACGASATDSKENTLISSKAGDVTVADTMKKIGKDQIANASFTEMLNKILADKYKNKVNDKKIDEQIEKMQKQYGGKDKFEKALQQQGLTADKYKENLRTAAYHKELLSDKIKISDSEIKEDSKKASHILIKVKSKKSDKEGLDDKEAKQKAEEIQKEVSKDPSKFGEIAKKESMDTGSAKKDGELGYVLKGQTDKYFEKALFKLKDGEVSDVVKSSFGYHIIKADKPTDFNSEKQSLKEKLVDQKVQKNPKLLTDAYKDLLKEYDVDFKDRDIKSVVEDKILNPEKLKQGGAQGGQSGMSQ

>HEI6216140.1 peptidylprolyl isomerase [Staphylococcus aureus]

MKMINKLIVPVTASALLLGACGASATDSKENTLISSKAGDVTVADTMKKIGKDQIANASFTEILNKILADKYKNKVNDKKIDEQIEKMQKQYGGKDKFKKALQQQGLTADKYKENLRTAAYHKELLSDKIKISDSEIKEDSKKASHILIKVKSKKSDKEGLDDKEAKQKAEEIQKEVSKDPSKFGEIAKKESMDTGSAKKDGELGYVLKGQTDKDFEKALFKLKDGEVSDVVKSSFGYHIIKADKPTDFNSEKQSLKEKLVDQKVQKNPKLLTDAYKDLLKEYDIDFKDRDIKSVVEDKILNPEKLKQGGAQGGQSGMSQ

>WJA90938.1 peptidylprolyl isomerase [Staphylococcus aureus]

MKMINKLIVPVTASALLLGACGASATDSKENTLISSKAGDVTVADTMKKIGKDQIANASFTEMLNKILADKYNNKVNDKKIDEQIEKMQKQYGGKDKFKKALQQQGLTADKYKENLRTAAYHKELLSDKIKISDSEIKEDSKKASHILIKVKSKKSDKEGLDDKEAKQKAEEIQKEVSKDPSKFGEIAKKESMDTGSAKKDGELGYVLKGQTDKDFEKALFKLKDGEVSDVVKSSFGYHIIKADKPTDFNSEKQSLKEKLVDQKVQKNPKLLTDAYKDLLKEYDVDFKDRDIKSVVEDKILNPEKLKQGGAQGGQSGMSQ

>WJB32853.1 peptidylprolyl isomerase [Staphylococcus aureus]

MKMINKLIVPVTASALLLGACGASATNSKENTLISSKAGDVTVADTMKKIGKDQIANASFTEMLNKILADKYKNKVNDKKIDEQIEKMQKQYGGKDKFEKALQQQGLTADKYKENLRTAAYHKELLSDKIKISDSEIKEDSKKASHILIKVKSKKSDKEGLDDKEAKQKAEEIQKEVSKDPSKFGEIAKKESMDTGSAKKDGELGYVLKGQTDKDFEKALFKLKDGEVSDVVKSSFGYHIIKADKPTDFNSEKQSLKEKLVDQKVQKNPKLLTDAYKDLLKEYDVDFKDRDIKSVVEDKILNPEKLKQGGAQGGQSGMSQ

>HBE8268187.1 peptidylprolyl isomerase [Staphylococcus aureus]

MKMINKLIVPVTASALLLGACGASATDFKENTLISSKAGDVTVADTMKKIGKDQIANASFTEMLNKILADKYKNKVNDKKIDEQIEKMQKQYGGKDKFEKALQQQGLTADKYKENLRTAAYHKELLSDKIKISDSEIKEDSKKASHILIKVKSKKSDKEGLDDKEAKQKAEEIQKEVSKDPSKFGEIAKKESMDTGSAKKDGELGYVLKGQTDKDFEKALFKLKDGEVSDVVKSSFGYHIIKADKPTDFNSEKQSLKEKLVDQKVQKNPKLLTDAYKDLLKEYDVDFKDRDIKSVVEDKILNPEKLKQGGAQGGQSGMSQ

>HDP6161316.1 peptidylprolyl isomerase [Staphylococcus aureus]

MKMINKLIVPVTASALLLGACGASATDSKENTLISSKAGDVTVADTMKKIGKDQIANASFTEMLNKILADKYKNKVNDKKIDEQIEKMQKQYGGKDKFEKALQQQGLTADKYKENLRTAAYHKELLSDKIKISDSEIKEDSKKASHILIKVKSKKSDKEGLDDKEAKQKAEEIQKEVSKDPSKFGEIAKKESMDTGFAKKDGELGYVLKGQTDKDFEKALFKLKDGEVSDVVKSSFGYHIIKADKPTDFNSEKQSLKEKLVDQKVQKNPKLLTDAYKDLLKEYDVDFKDRDIKSVVEDKILNPEKLKQGGAQGGQSGMSQ

>HCZ8826282.1 peptidylprolyl isomerase [Staphylococcus aureus]

MKMINKLIVPVTASALLLGACGASATDSKENTLISSKAGDVTVADTMKKIGKDQIANASFTEMLNKILADKYKNKVNDKKIDEQIEKMQKQYGGKDKFEKALQQQGLTADKYKENLRTAAYHKELLSDKIKISDSEIKEDSKKASHILIKVKSKKSDKEGLDDKEAKQKAEEIQKEVSKDPSKFGEIAKKESMDTGSAKKDGELGYVLKGQTDKDFEKALFKLKDGEVSDLVKSSFGYHIIKADKPTDFNSEKQSLKEKLVDQKVQKNPKLLTDAYKDLLKEYDVDFKDRDIKSVVEDKILNPEKLKQGGAQGGQSGMSQ

>WP_232044300.1 foldase protein PrsA [Staphylococcus aureus]

MKMINKLIVPVTASALLLGACGASATDSKENTLISSKAGDVTVADTMKKIGKDQIANASFTEMLNKILADKYKNKVNDKKIDEQIEKMQKQYGGKDKFEKALQQQGLTADKYKENLRTAAYHKELLSDKIKISDSEIKEDSKKASHILIKVKSKKSDKEGLDDKEAKQKAEEIQKEVSKDPSKFGEIAKKESMDTGSAKKDGELGYVLKGQTDKDFEKALFKLKDGQVSDVVKSSFGYHIIKADKPTDFNSEKQSLKEKLVDQKVQKNPKLLTDAYKDLLKEYDVDFKDRDIKSVVEDKILNPEKLKQGGAQGGQSGMSQ

>HDI6878029.1 peptidylprolyl isomerase [Staphylococcus aureus]

MKMINKLIVPVTASALLLGACGASATDSKENTLISSKAGDVTVADTMKKIGKDQIANASFTEMLNKILADKYKNKVNDKKIDEQIEKMQKQYGGKDKFKKALQQQGLTADKYKENLRTAAYHKELLSDKIKISDSEIKEDSKKASHILIKVKSKKSDKEGLDDKEAKQKAEEIQKEVSKDPSKFGEIAKKESMDTGSAKKDGELGYVLKGQTDKDFEKALFKLKDGEVSDVVKSSFGYHIIKADKPTDFNSEKQSLKEKLVDQKVQKNPKLLTDACKDLLKEYDVDFKDRDIKSVVEDKILNPEKLKQGGAQGGQSGMSQ

>HCY1654190.1 peptidylprolyl isomerase [Staphylococcus aureus]

MKMINKLIVPVTASALLLGACGASATDSKENTLISSKAGDVTVADTMKKIGKDQIANASFTEMLNKILADKYKNKVNDKKIDEQIEKMQKQYGGKDKFEKALQQQGLTADKYKENLRTAAYHKELLSDKIKISDSEIKEDSKKASHILIKVKSKKSDKEGLDDKEAKQKAEEIQKEVSKDPSKFGEIAKKETMDTGSAKKDGELGYVLKGQTDKDFEKALFKLKDGEVSDVVKSSFGYHIIKADKPTDFNSEKQSLKEKLVDQKVQKNPKLLTDAYKDLLKEYDVDFKDRDIKSVVEDKILNPEKLKQGGAQGGQSGMSQ

>HDC5770740.1 peptidylprolyl isomerase [Staphylococcus aureus]

MKMINKLIVPVTASALLLGACGASATDSKENTLISSKAGDVTVADTMKKIGKDQIANASFTEMLNKILADKYKNKVNDKKIDEQIEKMQKQYGGKDKFEKALQQQGLTADKYKENLRTAAYNKELLSDKIKISDSEIKEDSKKASHILIKVKSKKSDKEGLDDKEAKQKAEEIQKEVSKDPSKFGEIAKKESMDTGSAKKDGELGYVLKGQTDKDFEKALFKLKDGEVSDVVKSSFGYHIIKADKPTDFNSEKQSLKEKLVDQKVQKNPKLLTDAYKDLLKEYDVDFKDRDIKSVVEDKILNSEKLKQGGAQGGQSGMSQ

>HDG5824053.1 peptidylprolyl isomerase [Staphylococcus aureus]

MKMINKLIVPVTASALLLGACGASATDSKENTLISSKAGDVTVADTMKKIGKDQIANASFTEMLNKILADKYKNKVNDKKIDEQIEKMQKQYGGKDKFEKALQQQGLTADKYKENLRTAAYHKELLSDKIKISDSEIKEDSKKASHILIKVKSKKSDKEGLDDKEAKQKAEEIQKEVSKDPSKFGEIAKKESMDTGSAKKDGELGYVLKGQTDKDFEKALFKLKDGEVSDVVKSSFGYHIIKADKPTDFNSEKQSLKEKLVDQIVQKNPKLLTDAYKDLLKEYDVDFKDRDIKSVVEDKILNPEKLKQGGAQGGQSGMSQ

>WJC87878.1 peptidylprolyl isomerase [Staphylococcus aureus]

MKMINKLIVPVTASALLLGACGASATDSKENTLISSKAGDVTVADTMKKIGKDQIANASFTEMLNKILADKYKNKVNDKKIDEQIEKMQKQYGGKDKFKKALQQQGLTADKYKENLRTAAYHKELLSDKIKISDSEIKEDSKKASHILIKVKSKKSDNEGLDDKEAKQKAEEIQKEVSKDPSKFGEIAKKESMDTGSAKKDGELGYVLKGQTDKDFEKALFKLKDGEVSDVVKSSFGYHIIKADKPTDFNSEKQSLKEKLVDQKVQKNPKLLTDAYKDLLKEYDVDFKDRDIKSVVEDKILNPEKLKQGGAQGGQSGMSQ

>WP_064272732.1 foldase protein PrsA [Staphylococcus aureus]

MKMINKLIVPVTASALLLGACGASATDSKENTLISSKAGDVTVADTMKKIGKDQIANASFTEMLNKILADKYKNKVNDKKIDEQIEKMQKQYGGKDKFKNALQQQGLTADKYKENLRTAAYHKELLSDKIKISDSEIKEDSKKASHILIKVKSKKSDKEGLDDKEAKQKAEEIQKEVSKDPSKFGEIAKKESMDTGSAKKDGELGYVLKGQTDKDFEKALFKLKDGEVSDVVKSSFGYHIIKADKPTDFNSEKQSLKEKLVDQKVQKNPKLLTDAYKDLLKEYDVDFKDRDIKSVVEDKILNPEKLKQGGAQGGQSGMSQ

>HDJ3222964.1 peptidylprolyl isomerase [Staphylococcus aureus]

MKMINKLIVPVTASALLLGACGASATDSKENTLISSKAGDVTVADTMKKIGKDQIANASFTEMLNKILADKYKNKVNDKKIDEQIEKMQKQYGGKDKFEKALQQQGLTADKYKENLRTAAYHKELLSDKIKISDSEIKEDSKKASHILIKVKSKKSDKEGLDDKEAKQKAEEIQKEVSKDPSKFGEIAKKESMDTGSAKKDGELGYVLKGQTDKDFEKALFKLKDGEVSDVVKSSFGYHIIKADKPTDFNSEKQSLKEKLVDQEVQKNPKLLTDAYKDLLKEYDVDFKDRDIKSVVEDKILNPEKLKQGGAQGGQSGMSQ

>HDA7934334.1 peptidylprolyl isomerase [Staphylococcus aureus]

MKMINKLIVPVTASALLLGACGASATDSKENTLISSKAGDVTVADTMKKIGKDQIANASFTEMLNKILADKYKNKVNDKKIDEQIEKMQKQYGGKDKFEKALQQQGLTADKYKENLRTAAYHKELLSDKIKISDSEIKEDSKKASHILIKVKSKKSDKEGLDDKEAKQKAEEIQKEVSKDPSKFGEIAKKESMDTGSAKKDGELGYVLKGQTDKDFEKALFKLKDGEVSDVVKSSFGYHIIKADEPTDFNSEKQSLKEKLVDQKVQKNPKLLTDAYKDLLKEYDVDFKDRDIKSVVEDKILNPEKLKQGGAQGGQSGMSQ

>HCX2479029.1 peptidylprolyl isomerase [Staphylococcus aureus]

MKMINKLIVPVTASALLLGACGASATDSKENTLISSKAGDVTVADTMKKIGKDQIANASFTEMLNKILADKYKNKVNDKKIDEQIEKMQKQYGGKDKFEKALQQQGLTADKYKENLRIAAYHKELLSDKIKISDSEIKEDSKKASHILIKVKSKKSDKEGLDDKEAKQKAEEIQKEVSKDPSKFGEIAKKESMDTGSAKKDGELGYVLKGQTDKDFEKALFKLKDGEVSDVVKSSFGYHIIKADKPTDFNSEKQSLKEKLVDQKVQKNPKLLTDAYKDLLKEYDVDFKDRDIKSVVEDKILNPEKLKQGGAKGGQSGMSQ

>HEI6695556.1 peptidylprolyl isomerase [Staphylococcus aureus]

MKMINKLIVPVTASALLLGACGASATDSKENTLISSKAGDVTVADTMKKIGKDQIANASFTEMLNKILADKYKNEVNDKKIDEQIEKMQKQYGGKDKFEKALQQQGLTADKYKENLRTAAYHKELLSDKIKISDSEIKEDSKKASHILIKVKSKKSDKEGLDDKEAKQKAEEIQKEVSKDPSKFGEIAKKESMDTGSAKKDGELGYVLKGQTDKDFEKALFKLKDGEVSDVVKSSFGYHIIKADKPTDFNSEKQSLKEKLVDQKVQKNPKLLTDAYKDLLKEYDVDFKDRDIKSVVEDKILNPEKLKQGGAQGGQSGMSQ

>HAR2919294.1 peptidylprolyl isomerase [Staphylococcus aureus]

MKMINKLIVPVTASALLLGACGASATDSKENTLISSKAGDVTVADTMKKIGKDQIANASFTEMLNKILADKYKNKVNDKKIDEQIEKMQKQYGGKDKFEKALQQQGLTADKYKENLRTAAYHKELLSDKIKISDSEIKEDSKKASHILIKVKSKKSDKEGLDDKEAKQKAEEIQKEVSKDPSKFGEIAKKESMDTGSAKKDGELGYVLKGQTDKDFEKALFKLKDGEVSDVVKSSFGYHIIKADKPTDFNSEKQSLKEKLVDQKVQKNPKLLTDAYKDLLKEY

>WP_151097574.1 foldase protein PrsA, partial [Staphylococcus aureus]

MKMINKLIVPVTASALLLGACGASATDSKENTLISSKAGDVTVADTMKKIGKDQIANASFTEMLNKILADKYKNKVNDKKIDEQIEKMQKQYGGKDKFEKALQQQGLTADKYKENLRTAAYHKELLSDKIKISDSEIKEDSKKASHILIKVKSKKSDKEGLDDKEAKQKAEEIQKEVSKNPSKFGEIAKKESMDTGSAKKDGELGYVLKGQTDKDFEKALFKLKDGEVSDVVKSSFGYHIIKADKPTDFNSEKQSLKEKLVDQKVQKNPKLLTDAYKDLLKEYDVDFKDRDIKSVVEDK

>HDG9731167.1 peptidylprolyl isomerase [Staphylococcus aureus]

MKMINKLIVPVTASALLLGACGASATDSKENTLISSKAGDVTVADTMKKIGKDQIANASFTEMLNKILADKYKNKVNDKKIDEQIEKMQKQYGGKDKFKKALQQQGLTADKYKENLRTAAYHKELLSDKIKISDSEIKEDSKKASHILIKVKSKKSDKEGLDDKEAKQKAEEIQKEVSKDPSKFGEIAKKESMDTGSAKKDGELGYVLKGQTDKDFEKALFKLKDGEVSDVVKSSFGYHIIKADKPTDFNSEKQSLKEKLVDQKVQKNPKLLTDAYKDLLKEYDVNFKDRDIKSVVEDKILNPEKLKQGGAQGGQSGMSQ

>HDN3487939.1 peptidylprolyl isomerase [Staphylococcus aureus]

MKMINKLIVPVTASALLLGACGASATDSKENTLISSKAGDVTVADTMKKIGKDQIANASFTEMLNKILADKYKNKVNDKKIDEQIEKMQKQYGGKDKFEKALQQQGLTADKYKENLRTAAYHKELLSDKIKISDSEIKEDSKKASHILIKVKSKKSDKEGLDDKEAKQKAEEIQKEVSKDPSKFGEIAKKESMDTGSAKKDGELGYVLKGQTDKDFEKALFKLKDGEVSDVVKSSFGYHIFKADKPTDFNSEKQSLKEKLVDQKVQKNPKLLTDAYKDLLKEYDVDFKDRDIKSVVEDKILNPEKLKQGGAQGGQSGMSQ

>HCD7050340.1 peptidylprolyl isomerase [Staphylococcus aureus]

MKMINKLIVPVTASALLLGACGASATDSKENTLISSKAGDVTVADTMKKIGKDQIANASFTEMLNKILADKYKNKVNDKKIDEQIEKMQKQYGGKDKFEKALQQQGLTADKYKENLRTAAYHKELLSDKIKISDSEIKEDSKKASHILIKVKSKKSDKEGLDDKEAKQKAEEIQKEVSKNPSKFGEIAKKESMDTGSAKKDGELGYVLKGQTDKDFEKALFKLKDGEVSDVVKSSFGYHIIKSDKPTDFNSEKQSLKEKLVDQKVQKNPKLLTDAYKDLLKEYDVDFKDRDIKSVVEDKILNPEKLKQGGAQGGQSGMSQ

>HCW8221251.1 peptidylprolyl isomerase [Staphylococcus aureus]

MKMTNKLIVPVTASALLLGACGASATDSKENTLISSKAGDVTVADTMKKIGKDQIANASFTEMLNKILADKYKNKVNDKKIDEQIEKMQKQYGGKDKFKKALQQQGLTADKYKENLRTAAYHKELLSDKIKISDSEIKEDSKKASHILIKVKSKKSDKEGLDDKEAKQKAEEIQKEVSKDPSKFGEIAKKESMDTGSAKKDGELGYVLKGQTDKDFEKALFKLKDGEVSDVVKSSFGYHIIKADKPTDFNSEKQSLKEKLVDQKVQKNPKLLTDAYKDLLKEYDVDFKDRDIKSVVEDKILNPEKLKQGGAQGGQSGMSQ

>HDD2311965.1 peptidylprolyl isomerase [Staphylococcus aureus]

MKMINKLIVPVTASALLLGACGASATDSKENTLISSKAGDVTVADTMKKIGKDQIANASFTEMLNKILADKYKNKVNDKKIDEQIEKMQKQYGGKDKFKKALQQQGLTADKYKENLRTAAYHKELLSDKIKISDSEIKEDSKKASHILIKVKSKKSDKEGLDDKEAKQKAEEIQKEVSKGPSKFGEIAKKESMDTGSAKKDGELGYVLKGQTDKDFEKALFKLKDGEVSDVVKSSFGYHIIKADKPTDFNSEKQSLKEKLVDQKVQKNPKLLTDAYKDLLKEYDVDFKDRDIKSVVEDKILNPEKLKQGGAQGGQSGMSQ

>HCZ6519511.1 peptidylprolyl isomerase [Staphylococcus aureus]

MKMINKLIVPVTASALLLGACGASATDSKENTLISSKAGDVTVADTMKKIGKDQIANASFTEMLNKILADKYKNKVNDKKIDEQIEKMQKQYGGKDKFKKALQQQGLTADKYKENLRTAAYHKELLSDKIKISDSEIKEDSKKASHILIKVKSKKSDKEGLDDKEAKQKAEEIQKEVSKDPSKFGEIAKKESMDTGSAKKDGELGYVLKGQTDKDFEKALFKLKDGEVSDVVKSSFGYHIIKADKPTDFNSEKQSLKEKLVDQKVQKNPKLLTDAYKDLLKEYDVDFKDHDIKSVVEDKILNPEKLKQGGAQGGQSGMSQ

>WP_031864047.1 foldase protein PrsA [Staphylococcus aureus]

MKMINKLIVPVTASALLLGACGASATDSKENTLISSKAGDVTVADTMKKIGKDQIANASFTEMLNKILADKYKNKVNDKKIDEQIEKMQKQYGGKDKFEKALQQQGLTADKYKENLRTAAYHKELLSDKIKISDSEIKEDSKKASHILIKVKSKKSDKEGLDDKEAKQKAEEIQKEVSKDPSKFGEIGKKESMDTGSAKKDGELGYVLKGQTDKDFEKALFKLKDGEVSDVVKSSFGYHIIKADKPTDFNSEKQSLKEKLVDQKVQKNPKLLTDAYKDLLKEYDVDFKDRDIKSVVEDKILNPEKLKQGGAQGGQSGMSQ

>HCZ0743758.1 peptidylprolyl isomerase [Staphylococcus aureus]

MKMINKLIVPVTASALLLGACGASATDSKENTLISSKAGDVTVADTMKIIGKDQIANASFTEMLNKILADKYKNKVNDKKIDEQIEKMQKQYGGKDKFEKALQQQGLTADKYKENLRTAAYHKELLSDKIKISDSEIKEDSKKASHILIKVKSKKSDKEGLDDKEAKQKAEEIQKEVSKDPSKFGEIAKKESMDTGSAKKDGELGYVLKGQTDKDFEKALFKLKDGEVSDVVKSSFGYHIIKADKPTDFNSEKQSLKEKLVDQKVQKNPKLLTDAYKDLLKEYDVDFKDRDIKSVVEDKILNPEKLKQGGAQGGQSGMSQ

>HAY2223817.1 peptidylprolyl isomerase [Staphylococcus aureus]

MKMINKLIVPVTASALLLGACGASATDSKENTLISSKAGDVTVADTMKKIGKDQIANASFTEMLNKILADKYKNKVNDKKIDEQIEKMQKQYGGKDKFEKALQQQGLTADKYKENLRTAAYHKELLSDKIKISDSEIKEDSKKASHILIKVKSKKSDKEGLDDKEAKQKAEEIQKEVSKNPSKFGEIAKKESMDTGSAKKDGELGYVLKGQTDKDFEKALFKLKDGEVSDVVKSSFGYHIIKADKPTDFNSEKQSLKEKLVDQKVQKNPKLLTDAYKDLLKEYDVDFKDRDIKSVVENKILNPEKLKQGGAQGGQSGMSQ

>HDP2522369.1 peptidylprolyl isomerase [Staphylococcus aureus]

MKMINKLIVPVTASALLLGACGASATDSKENTLISSKAGDVTVADTMKKIGKDQIANASFTEMLNKILADKYKNKVNDKKIDEQIEKMPKQYGGKDKFKKALQQQGLTADKYKENLRTAAYHKELLSDKIKISDSEIKEDSKKASHILIKVKSKKSDKEGLDDKEAKQKAEEIQKEVSKDPSKFGEIAKKESMDTGSAKKDGELGYVLKGQTDKDFEKALFKLKDGEVSDVVKSSFGYHIIKADKPTDFNSEKQSLKEKLVDQKVQKNPKLLTDAYKDLLKEYDVDFKDRDIKSVVEDKILNPEKLKQGGAQGGQSGMSQ

>WP_276123766.1 foldase protein PrsA [Staphylococcus aureus]

MKMINKLIVPVTASALLLGACGASATDSKENTLISSKAGDVTVADTMKKIGKDQIANASFTEMLNKILADKYKNKVNDKKIDEQIEKMQKQYGGKDKFKKALQQQGLTADKYKENLRTAAYHKELLSDKIKISDSEIKEDSKKASHILIKVKSKKSDKEGLDDKEAKQKAEEIQKEVSKDPSKFGEIAKKESMDTGSAKKDGELSYVLKGQTDKDFEKALFKLKDGEVSDVVKSSFGYHIIKADKPTDFNSEKQSLKEKLVDQKVQKNPKLLTDAYKDLLKEYDVDFKDRDIKSVVEDKILNPEKLKQGGAQGGQSGMSQ

>WP_085065317.1 foldase protein PrsA [Staphylococcus aureus]

MKMINKLIVPVTASALLLGACGASATDSKENTLISSKAGDVTVADTMKKIGKDQIANASFTEMLNKILADKYKNKVNDKKIDEQIEKMQKQYGGKDKFEKALQQQGLTADKYKENLRTAAYHKELLSDKIKISDSEIKEDSKKASHILIKVKSKKSDKEGLDDKEAKQKAEEIQKEVSKDPSKFGEIAKKESMDTGSAKKDGELGYVLKGQTDKDFEKALFKLKDGEVSDVVKSSFGYHIIKADKPTEFNSEKQSLKEKLVDQKVQKNPKLLTDAYKDLLKEYDVDFKDRDIKSVVEDKILNPEKLKQGGAQGGQSGMSQ

>WP_250425927.1 foldase protein PrsA [Staphylococcus aureus]

MKMINKLIVPVTASALLLGACGASATDSKENTLISSKAGDVTVADTMKKIGKDQIANASFTEMLNKILADKYKNKVNDKKINEQIEKMQKQYGGKDKFEKALQQQGLTADKYKENLRTAAYHKELLSDKIKISDSEIKEDSKKASHILIKVKSKKSDKEGLDDKEAKQKAEEIQKEVSKDPSKFGEIAKKESMDTGSAKKDGELGYVLKGQTDKDFEKALFKLKDGEVSDVVKSSFGYHIIKADKPTDFNSEKQSLKEKLVDQKVQKNPKLLTDAYKDLLKEYDVDFKDRDIKSVVEDKILNPEKLKQGGAQGGQSGMSQ

>UXV54935.1 peptidylprolyl isomerase [Staphylococcus aureus]

MKMINKLIVPVTASALLLGACGASATDSKENTLISSKAGDVTVADTMKKIGKDQIANASFTEMLNKILADKYKNKVNDKKIDEQIEKMQKQYGGKDKFEKALQQQGLTADKYKENLRTAAYHKELLSDKIKISDSEIKEDSKKASHILIKVKSKKSDKEGLDDKEAKQKAEEIQKEVSKDPSKFGEIAKKESMDTGSAKKDGELGYVLKGQTDKDFEKALFKLKDGEVSDVVKSSFGYHIIKADKPTDFNSEKQSLKEKLVDQKVQKNPKLLTDAYKDLLKEYDVDFKNRDIKSVVEDKILNPEKLKQGGAQGGQSGMSQ

>WP_000782129.1 foldase protein PrsA [Staphylococcus aureus]

MKMINKLIVPVTASALLLGACGASATDSKENTLISSKAGDVTVADTMKKIGKDQIANASFTEMLNKILADKYKNKVNDKKIDEQIEKMQKQYGGKDKFEKALQQQGLTADKYKENLRTAAYHKELLSDKIKISDSEIKEDSKKASHILIKVKSKKSDKEGLDDKEAKQKAEEIQKEVSKNPSKFGEIAKKESMDTGSAKKDGELGYVLKGQTDKDFEKALFKLKDGEVSDVVKSSFGYHIIKADKPTDFNSEKQSLKEKLVDQKVQKNPKLLTDAYKDLLKEYDVDFKDRDIKSVVEDKILNPEKLKQGGAQGGQSGMSQ

>HEI6461334.1 peptidylprolyl isomerase [Staphylococcus aureus]

MKMINKLIVPVTASALLLGACGASATDSKENTLISSKAGDVTVADTMKKIGKDQIANASFTEMLNKILADKYKNKVNDKKIDEQIEKMQKQYGGKDKFEKALQQQGLTANKYKENLRTAAYHKELLSDKIKISDSEIKEDSKKASHILIKVKSKKSDKEGLDDKEAKQKAEEIQKEVSKDPSKFGEIAKKESMDTGSAKKDGELGYVLKGQTDKDFEKALFKLKDGEVSDVVKSSFGYHIIKADKPTDFNSEKQSLKEKLVDQKVQKNPKLLTDAYKDLLKEYDVDFKDRDIKSVVEDKILNPEKLKQGGAQGGQSGMSQ

>HCZ5545205.1 peptidylprolyl isomerase [Staphylococcus aureus]

MKMINKLIVPVTASALLLGACGASAKDSKENTLISSKAGDVTVADTMKKIGKDQIANASFTEMLNKILADKYKNKVNDKKIDEQIEKMQKQYGGKDKFKKALQQQGLTADKYKENLRTAAYHKELLSDKIKISDSEIKEDSKKASHILIKVKSKKSDKEGLDDKEAKQKAEEIQKEVSKDPSKFGEIAKKESMDTGSAKKDGELGYVLKGQTDKDFEKALFKLKDGEVSDVVKSSFGYHIIKADKPTDFNSEKQSLKEKLVDQKVQKNPKLLTDAYKDLLKEYDVDFKDRDIKSVVEDKILNPEKLKQGGAQGGQSGMSQ

>HDH5899280.1 peptidylprolyl isomerase [Staphylococcus aureus]

MKMINKLIVPVTASALLLGACGASATDSKENTLISSKAGDVTVADTMKKIGKDQIANASFTEMLNKILADKYKNKVNDKKIDEQIEKMQKQYGGKEKFEKALQQQGLTADKYKENLRTAAYHKELLSDKIKISDSEIKEDSKKASHILIKVKSKKSDKEGLDDKEAKQKAEEIQKEVSKDPSKFGEIAKKESMDTGSAKKDGELGYVLKGQTDKDFEKALFKLKDGEVSDVVKSSFGYHIIKADKPTDFNSEKQSLKEKLVDQKVQKNPKLLTDAYKDLLKEYDVDFKDRDIKSVVEDKILNPEKLKQGGAQGGQSGMSQ

>HCY1361087.1 peptidylprolyl isomerase [Staphylococcus aureus]

MKMINKLIGPVTASALLLGACGASATDSKENTLISSKAGDVTVADTMKKIGKDQIANASFTEMLNKILADKYKNKVNDKKIDEQIEKMQKQYGGKDKFEKALQQQGLTADKYKENLRTAAYHKELLSDKIKISDSEIKEDSKKASHILIKVKSKKSDKEGLDDKEAKQKAEEIQKEVSKDPSKFGEIAKKESMDTGSAKKDGELGYVLKGQTDKDFEKALFKLKDGEVSDVVKSSFGYHIIKADKPTDFNSEKQSLKEKLVDQKVQKNPKLLTDAYKDLLKEYDVDFKDRDIKSVVEDKILNPEKLKQGGAQGGQSGMSQ

>WP_072489939.1 foldase protein PrsA [Staphylococcus aureus]

MKMINKLIVPVTASALLLGACGASATDSKENTLIFSKAGDVTVADTMKKIGKDQIANASFTEMLNKILADKYKNKVNDKKIDEQIEKMQKQYGGKDKFEKALQQQGLTADKYKENLRTAAYHKELLSDKIKISDSEIKEDSKKASHILIKVKSKKSDKEGLDDKEAKQKAEEIQKEVSKDPSKFGEIAKKESMDTGSAKKDGELGYVLKGQTDKDFEKALFKLKDGEVSDVVKSSFGYHIIKADKPTDFNSEKQSLKEKLVDQKVQKNPKLLTDAYKDLLKEYDVDFKDRDIKSVVEDKILNPEKLKQGGAKGGQSGMSQ

>MBU7894004.1 peptidylprolyl isomerase [Staphylococcus aureus]

MKMINKLIVPVTASALLLGACGACGASATDSKENTLISSKAGDVTVADTMKKIGKDQIANASFTEMLNKILADKYKNKVNDKKIDEQIEKMQKQYGGKDKFKKALQQQGLTADKYKENLRTAAYHKELLSDKIKISDSEIKEDSKKASHILIKVKSKKSDKEGLDDKEAKQKAEEIQKEVSKDPSKFGEIAKKESMDTGSAKKDGELGYVLKGQTDKDFEKALFKLKDGEVSDVVKSSFGYHIIKADKPTDFNSEKQSLKEKLVDQKVQKNPKLLTDAYKDLLKEYDVDFKDRDIKSVVEDKILNPEKLKQGGAQGGQSGMSQ

>HDH6588020.1 peptidylprolyl isomerase [Staphylococcus aureus]

MKMINKLIVPVTASALLLGACGASATDSKENTLISSKAGDVTVADTMKKIGKDQIANASFTEMLNKISADKYKNKVNDKKIDEQIEKMQKQYGGKDKFEKALQQQGLTADKYKENLRTAAYHKELLSDKIKISDSEIKEDSKKASHILIKVKSKKSDKEGLDDKEAKQKAEEIQKEVSKDPSKFGEIAKKESMDTGSAKKDGELGYVLKGQTDKDFEKALFKLKDGEVSDVVKSSFGYHIIKADKPTDFNSEKQSLKEKLVDQKVQKNPKLLTDAYKDLLKEYDVDFKDRDIKSVVEDKILNPEKLKQGGAQGGQSGMSQ

>HCZ2736514.1 peptidylprolyl isomerase [Staphylococcus aureus]

MKMINKLIVPVTASALLLGACGASATDSKENTLISSKAGDVTVADTMKKIGKDQIANASFTEMLNKILADKYKNKVNDKKIDEQIEKMQKQYGGKDKFEKALQQQGLTADKYKENLRTAAYHKELLSDKIKISDSEIKEDSKKASHILIKVKSKKSDKEGLDDKEAKQKAEEIQKEVSKNPSKFGEIAKKESMDTGSAKKDGELGYVLKGQTDKDFEKALFKLKDGEVSDVVKSSFGYHIIKADKPTDFNSEKQSLKEKLVDQKVQKNPKLLTDAYKDLLKEYDVDFKDRDIKSVVEDKILNPAKLKQGGAQGGQSGMSQ

>HDH6983010.1 peptidylprolyl isomerase [Staphylococcus aureus]

MKMINKLIVPVTASALLLGACGASATDSKENTLISSKAGDVTVADTMKKIGKDQIANASFTEMLNKILADKYKNKVNDKKIDEQIEKMQKQYGGKDKFEKALQQQGLTADKYKENLRTAAYHKELLSDKIKISDSEIKEDSKKASHILIKVKSKKSDKEGLDDKEAKQKAEEIQKEVSKDPSKFGEIAKKESMDTGSAKKDGELGYVLKGQTDKDFEKVLFKLKDGEVSDVVKSSFGYHIIKADKPTDFNSEKQSLKEKLVDQKVQKNPKLLTDAYKDLLKEYDVDFKDRDIKSVVEDKILNPEKLKQGGAQGGQSGMSQ

>HCY3056978.1 peptidylprolyl isomerase [Staphylococcus aureus]

MKMINKLIVPVTASALLLGACSASATDSKENTLISSKAGDVTVADTMKKIGKDQIANASFTEMLNKILADKYKNKVNDKKIDEQIEKMQKQYGGKDKFKKALQQQGLTADKYKENLRTAAYHKELLSDKIKISDSEIKEDSKKASHILIKVKSKKSDKEGLDDKEAKQKAEEIQKEVSKDPSKFGEIAKKESMDTGSAKKDGELGYVLKGQTDKDFEKALFKLKDGEVSDVVKSSFGYHIIKADKPTDFNSEKQSLKEKLVDQKVQKNPKLLTDAYKDLLKEYDVDFKDRDIKSVVEDKILNPEKLKQGGAQGGQSGMSQ

>HDI7492144.1 peptidylprolyl isomerase [Staphylococcus aureus]

MKMINKLIVPVTASALLLGACGVSATDSKENTLISSKAGDVTVADTMKKIGKDQIANASFTEMLNKILADKYKNKVNDKKIDEQIEKMQKQYGGKDKFEKALQQQGLTADKYKENLRTAAYHKELLSDKIKISDSEIKEDSKKASHILIKVKSKKSDKEGLDDKEAKQKAEEIQKEVSKDPSKFGEIAKKESMDTGSAKKDGELGYVLKGQTDKDFEKALFKLKDGEVSDVVKSSFGYHIIKADKPTDFNSEKQSLKEKLVDQKVQKNPKLLTDAYKDLLKEYDVDFKDRDIKSVVEDKILNPEKLKQGGAQGGQSGMSQ

>WP_215803948.1 foldase protein PrsA [Staphylococcus aureus]

MKMINKLIVPVTASALLLGACGASATDSKENTLISSKAGDVTVADTMKKIGKDQIANASFTEMLNKILADKYKNKVNDKKIDEQIEKMQKQYGGKDKFKKALQQQGLTADKYKENLRTAAYHKELLSDKIKISDSEIKEDSKKASHILIKVKSKKSDKESLDDKEAKQKAEEIQKEVSKDPSKFGEIAKKESMDTGSAKKDGELGYVLKGQTDKDFEKALFKLKDGEVSDVVKSSFGYHIIKADKPTDFNSEKQSLKEKLVDQKVQKNPKLLTDAYKDLLKEYDVDFKDRDIKSVVEDKILNPEKLKQGGAQGGQSGMSQ

>MBG1229454.1 peptidylprolyl isomerase [Staphylococcus aureus]

MINKLIVPVTASALLLGACGASATDSKENTLISSKAGDVTVADTMKKIGKDQIANASFTEMLNKILADKYKNKVNDKKIDEQIEKMQKQYGGKDKFKKALQQQGLTADKYKENLRTAAYHKELLSDKIKISDSEIKEDSKKASHILIKVKSKKSDKEGLDDKEAKQKAEEIQKEVSKDPSKFGEIAKKESMDTGSAKKDGELGYVLKGQTDKDFEKALFKLKDGEVSDVVKSSFGYHIIKADKPTDFNSEKQSLKEKLVDQKVQKNPKLLTDAYKDLLKEYDVDFKDRDIKSVVEDKILNPEKLKQGGAQGGQSGMSQ

>HCY6500575.1 peptidylprolyl isomerase [Staphylococcus aureus]

MKMINKLIVPVTASALLLGACGASATDSKENTLISSKAGDVTVADTMKKIGKDQIANASFTEMLNKILADKYKNKVNDKKIDEQIEKMQKQYGGKDKFKKALQQQGLTADKYKENLRTAAYHKELLSDKIKISDSEIKEDSKKASHILIKVKSKKSDKEGLDDKEAKQKAEEIQKEVSKDSSKFGEIAKKESMDTGSAKKDGELGYVLKGQTDKDFEKALFKLKDGEVSDVVKSSFGYHIIKADKPTDFNSEKQSLKEKLVDQKVQKNPKLLTDAYKDLLKEYDVDFKDRDIKSVVEDKILNPEKLKQGGAQGGQSGMSQ

>HCZ8132429.1 peptidylprolyl isomerase [Staphylococcus aureus]

MKMINKLIVPVTASALLLGACGASATDSKENTLISSKAGDVTVADTMKKIGKDQIANASFTEMLNKILADKYKNKVNDKKIDEQIEKMQKQYGGKDKFEKALQQQGLTADKYKENLRTAAYHKELLSDKIKISDSEIKEDSKKASHILIKVKSKKSDKEGLDDKEAKQKAEEIQKEVSKDPSKFGEIAKKESMDPGSAKKDGELGYVLKGQTDKDFEKALFKLKDGEVSDVVKSSFGYHIIKADKPTDFNSEKQSLKEKLVDQKVQKNPKLLTDAYKDLLKEYDVDFKDRDIKSVVEDKILNPEKLKQGGAQGGQSGMSQ

>HDJ7356838.1 peptidylprolyl isomerase [Staphylococcus aureus]

MKMINKLIVPVTASALLLGACGASATDSKENTLISSKAGDVTVADTMKKIGKDQIANASFTEMLNKILADKYKNKVNDKKIDEQIEKMQKQYGGKDKFEKALQQQGLTADKYKENLRTAAYHKELLSDKIKISDSEIKEDSKKASHILIKVKSKKSDKEGLDDKEAKQKAEEIQKEVSKDPSKFGEIAKKESMDTGSAKKDGELGYVLKGQTDKDFEKALFNLKDGEVSDVVKSSFGYHIIKADKPTDFNSEKQSLKEKLVDQKVQKNPKLLTDAYKDLLKEYDVDFKDRDIKSVVEDKILNPEKLKQGGAQGGQSGMSQ

>HCY3071674.1 peptidylprolyl isomerase [Staphylococcus aureus]

MKMINKLIVPVTASALLLGACGASATDSKENTLISSKAGDVTVADTMKKIGKDQIANASFTEMLNNILADKYKNKVNDKKIDEQIEKMQKQYGGKDKFEKALQQQGLTADKYKENLRTAAYHKELLSDKIKISDSEIKEDSKKASHILIKVKSKKSDKEGLDDKEAKQKAEEIQKEVSKDPSKFGEIAKKESMDTGSAKKDGELGYVLKGQTDKDFEKALFKLKDGEVSDVVKSSFGYHIIKADKPTDFNSEKQSLKEKLVDQKVQKNPKLLTDAYKDLLKEYDVDFKDRDIKSVVEDKILNPEKLKQGGAQGGQSGMSQ

>WP_031886449.1 foldase protein PrsA [Staphylococcus aureus]

MKMINKLIVPVTASALLLGACGASATDSKENTLISSKAGDVTVADTMKKIGKDQIANASFTEMLNKILADKYKNKVNDKKIDEQIEKMQNQYGGKDKFEKALQQQGLTADKYKENLRTAAYHKELLSDKIKISDSEIKEDSKKASHILIKVKSKKSDKEGLDDKEAKQKAEEIQKEVSKDPSKFGEIAKKESMDTGSAKKDGELGYVLKGQTDKDFEKALFKLKDGEVSDVVKSSFGYHIIKADKPTDFNSEKQSLKEKLVDQKVQKNPKLLTDAYKDLLKEYDVDFKDRDIKSVVEDKILNPEKLKQGGAQGGQSGMSQ

>HCV3683336.1 peptidylprolyl isomerase [Staphylococcus aureus]

MKMINKLIVPVTASALLLGACGASATDSKENKLISSKAGDVTVADTMKKIGKDQIANASFTEMLNKILADKYKNKVNDKKIDEQIEKMQKQYGGKDKFKKALQQQGLTADKYKENLRTAAYHKELLSDKIKISDSEIKEDSKKASHILIKVKSKKSDKEGLDDKEAKQKAEEIQKEVSKDPSKFGEIAKKESMDTGSAKKDGELGYVLKGQTDKDFEKALFKLKDGEVSDVVKSSFGYHIIKADKPTDFNSEKQSLKEKLVDQKVQKNPKLLTDAYKDLLKEYDVDFKDRDIKSVVEDKILNPEKLKQGGAQGGQSGMSQ

>HDP2836111.1 peptidylprolyl isomerase [Staphylococcus aureus]

MKMINKLIVPVTASALLLGACGASATDSKENTLISSKAGDVTVADTMKKIGKDQIANASFTEMLNKILADKYKNKVNDKKIDEQIEKMQKQYGGKDKFKKALQQQGLTADKYKENLRTAAYHKELLSDKIKISDSEIKEDSKKASHILIKVKSKKSDKEGLDDKEAKQKAEEIQKEVSKDPSKFGEIAKKESMDTGSAKKDGELGYVLKGQTDKDFEKALFKLKDGEVSDVVKSSFGYHIIKADKPTDFNSEKQSLKEKLVDQKVQKNPKLLTDAYKDLLKEYDVDFKDRDIKSVVEDKILNPEKLK

>HDK9404462.1 peptidylprolyl isomerase [Staphylococcus aureus USA1100-04031]

MKMINKLIVPVTASALLLGACGASATDSKENTLISSKAGDVTVADTMKKIGKDQIANASFTEMLNKILADKYKNKVNDKKIDEQIEKMQKQYGGKDKFKKALQQQGLTADKYKENLRTAAYHKELLSDKIKISDSEIKEDSKKASHILIKVKSKKSDKEGLDDKEAKQKAEEIQKEVSKDPSKFGEIAKKESMDTGSAKKDGELGYVLKGQTDKDFEKALFKLKDGEVSDVVKSSFGYHIIKADKPTDFNSEKQSLKEKLVDQKVQKNPKLLTDAYKDLLKEYDVDFKDRDIKSVVEDKILNPEKLKQGGAQGGQ

>WP_061742529.1 foldase protein PrsA [Staphylococcus aureus]

MKMINKLIVPVTASALLLGACGASATDSKENTLISSKAGDVTVADTMKKIGKDQIANASFTEMLNKILADKYKNKVNDKKIDEQIEKMQKQYGGKDKFEKALQQQGLTADKYKENLRTAAYHKELLSDKIKISDSEIKEDSKKASYILIKVKSKKSDKEGLDDKEAKQKAEEIQKEVSKDPSKFGEIAKKESMDTGSAKKDGELGYVLKGQTDKDFEKALFKLKDGEVSDVVKSSFGYHIIKADKPTDFNSEKQSLKEKLVDQKVQKNPKLLTDAYKDLLKEYDVDFKDRDIKSVVEDKILNPEKLKQGGAQGGQSGMSQ

>HCY8847185.1 peptidylprolyl isomerase [Staphylococcus aureus]

MKMINKLIVPVTASALLLGACGASATDSKENTLISSKAGDVTVADTMKKIGKDQIANASFTEMLNKILADKYKNKVNDKKIDEQIEKMQKQYGGKDKFKKALQQQGLTADKYKENLRTAAYHKELLSDKIKISDSEIKEDSKKASHILIKVKSKKSDKEGLDDKEAKQKAEEIQKEVSKDPSKFGEIAKKESMDTGSAKKDGELGYVLKGQTDKDFEKALFKLKDGEVSDVVKSSFGYHIIKADKPTDFNSEKQSLKEKLVDQKVQKNPKLLTDAYKDLLKEYDVDFKDRDIKSVVEDKILNPEKLKQGGGQSGMSQ

>HDG9639707.1 peptidylprolyl isomerase [Staphylococcus aureus]

MKMINKLIVPVTASALLLGACGASATDSKENTLISSKAGDVTVADTMKKIGKDQIANASFTEMLNKILADKYKNKFNDKKIDEQIEKMQKQYGGKDKFEKALQQQGLTADKYKENLRTAAYHKELLSDKIKISDSEIKEDSKKASHILIKVKSKKSDKEGLDDKEAKQKAEEIQKEVSKDPSKFGEIAKKESMDTGSAKKDGELGYVLKGQTDKDFEKALFKLKDGEVSDVVKSSFGYHIIKADKPTDFNSEKQSLKEKLVDQKVQKNPKLLTDAYKDLLKEYDVDFKDRDIKSVVEDKILNPEKLKQGGAQGGQSGMSQ

>HDC8005605.1 peptidylprolyl isomerase [Staphylococcus aureus]

MKMINKLIVPVTASALLLGACGASATDSKENTLISSKAGDVTVADTMKKIGKDQIANASFTEMLNKILADKYKNKVNDKKIDEQIEKMQKQYGGKDKFKKALQQQGLTADKYKENLRTAAYHKELLSDKIKISDSEIKEDSKKASHILIKVKSKKSDKEGLDDKEAKQKAEEIQKEVSKDPSKFGEIAKKESMDTGSAKKDGELGYVLKGQTDKDFEKALFKLKDGEVSDVVKSSFGYHIIKADKPTDFNSEKQSLKEKLVDQKVQKNPKLLTDAYKDLLKEYDVDFKDRDIKSVVKDKILNPEKLKQGGAQGGQSGMSQ

>HDI0304488.1 peptidylprolyl isomerase [Staphylococcus aureus]

MKMINKLIVPVTASALLLGACGASATDSKENTLISSKAGDVTVADTMKKIGKDQIANASFTEMLNKILADKHKNKVNDKKIDEQIEKMQKQYGGKDKFEKALQQQGLTADKYKENLRTAAYHKELLSDKIKISDSEIKEDSKKASHILIKVKSKKSDKEGLDDKEAKQKAEEIQKEVSKDPSKFGEIAKKESMDTGSAKKDGELGYVLKGQTDKDFEKALFKLKDGEVSDVVKSSFGYHIIKADKPTDFNSEKQSLKEKLVDQKVQKNPKLLTDAYKDLLKEYDVDFKDRDIKSVVEDKILNPEKLKQGGAQGGQSGMSQ

>HDC6109799.1 peptidylprolyl isomerase [Staphylococcus aureus]

MKMINKLIVPVTASALLLGACGASATDSKENTLISSKAGDVTVADTMKKIGKDQIANASFTEMLNKILADKYKNKVNDKKIDEQIEKMQKQYGGKDKFKKALQQQGLTADKYKENLRTAAYHKELLSDKIKISDSEIKEDSKKASHILIKVKSKKSDKEGIDDKEAKQKAEEIQKEVSKDPSKFGEIAKKESMDTGSAKKDGELGYVLKGQTDKDFEKALFKLKDGEVSDVVKSSFGYHIIKADKPTDFNSEKQSLKEKLVDQKVQKNPKLLTDAYKDLLKEYDVDFKDRDIKSVVEDKILNPEKLKQGGAQGGQSGMSQ

>MBS3325354.1 peptidylprolyl isomerase [Staphylococcus aureus]

MKMINKLIVPVTASALLLGACGASATDSKENTLISSKAGDVTVADTMKKIGKDQIANASFTEMLNKILADKYKNKVNDKKIDEQIEKMQKQYGGKDKFKKALQQQGLTADKYKENLRTAAYHKELLSDKIKISDSEIKEDSKKASHILIKVKSKKSDKEGLDDKEAKQKAEEIQKEVSKDPSKFGEIAKKESMDTGSAKKDGELGYVLKGQTDKDFEKALFKLKDGEVSDVVKSSFGYHIIKADKPTDFNSEKQSLKEKLVDQKVQKNPKLLTDAYKDLLKEYDVDFKDRDIKSVVEDKILNPEKLKQGGAQGGQFGMSQ

>HEP1244364.1 peptidylprolyl isomerase [Staphylococcus aureus]

MKMINKLIVPVTASALLLGACGASATDSKENTLISSKAGDVTVADTMKKIGKDQIANASFTEMLNKILADKYKNKVNDKKIDEQIEKMQKQYGGKDKFKKALQQQGLTADKYKENLRTAAYHKELLSDKIKISDSEIKEDSKKASHILIKVKSKKSDKEGLDDKEAKQKAEEIQKEVSKDPSKFGEIAKKESMDTGSAKKDGELGYVLKGQTDKDFEKALFKLKDGEVSDVVKSSFGYHIIKADKPTDFNSEKQSLKEKLVDQKVQKNPKLLTDAYKDLLKEYDVDFKDRDIKSVVEDKILNPEKLKQGGTQGGQSGMSQ

>WP_000782130.1 MULTISPECIES: foldase protein PrsA [Staphylococcus]

MKMINKLIVPVTASALLLGACGASATDSKENTLISSKAGDVTVADTMKKIGKDQIANASFTEMLNKILADKYKNKVNDKKIDEQIEKMQKQYGGKDKFKKALQQQGLTADKYKENLRTAAYHKELLSDKIKISDSEIKEDSKKASHILIKVKSKKSDKEGLDDKEAKQKAEEIQKEVSKDPSKFGEIAKKESMDTGSAKKDGELGYVLKGQTDKDFEKALFKLKDGEVSDVVKSSFGYHIIKADKPTDFNSEKQSLKEKLVDQKVQKNPKLLTDAYKDLLKEYDVDFKDRDIKSVVEDKILNPEKLKQGGAQGGQSGMSQ

>HDF9061531.1 peptidylprolyl isomerase [Staphylococcus aureus]

MKMINKLIVPVTASALLLGACGASATDSKENTLISSKAGDVTVADTMKKIGKDQIANASFTEMLNKILADKYKNKVNDKKIDEQIEKMQKQYGGKDKFKKALQQQGLTADKYKENLRTAAYHKELLSDKIKISDSEIKEDSKKASHILIKVKSKKSDKEGLDDKEAKQKAEEIQKEVSKDPSKFGEIAKKESMDTGSAKKDGELGYVLKGQTDKDFEKALFKLKDGEVSDVVKSSFGYHIIKADKPTDFNSEKQSLKEKLVDQKVQKNPKLLTDAYKDLLKEYDVDFKDRDIKSVVEDKILNPEKLKQGGPQGGQSGMSQ

>HDC9449567.1 peptidylprolyl isomerase [Staphylococcus aureus]

MKMINKLIVPVTASALLLGACGASATDSKENTLISSKAGDVTVADTMKKIGKDQIANASFTEMLNKILADKYKNKVNDKKIDEQIEKMQKQYGGKDKFKKALQQQGLTADKYKENLRTAAYHKELLSDKIKISDSEIKEDSKKTSHILIKVKSKKSDKEGLDDKEAKQKAEEIQKEVSKDPSKFGEIAKKESMDTGSAKKDGELGYVLKGQTDKDFEKALFKLKDGEVSDVVKSSFGYHIIKADKPTDFNSEKQSLKEKLVDQKVQKNPKLLTDAYKDLLKEYDVDFKDRDIKSVVEDKILNPEKLKQGGAQGGQSGMSQ

>HEK6648481.1 peptidylprolyl isomerase [Staphylococcus aureus]

MKMINKLIVPVTASALLLGACGASATDSKENTLISSKAGDVTVADTMKKIGKDQIANASFTEMLNKILADKYKNKVNDKKIDEQIEKMQKQYGGKDKFKKALQQQGLTADKYKENLRTAAYHKELLSDKIKISDSEIKEDSKKASHILIKVKSKKSDKEGLDDKEAKQKAEEIQKEVSKDPSKFGEIAKKESMDTGSAKKDGELGYVLKGQTDKDFEKALFKLKDGEVSDVVKSSFGYHIIKADKPTDFNNEKQSLKEKLVDQKVQKNPKLLTDAYKDLLKEYDVDFKDRDIKSVVEDKILNPEKLKQGGAQGGQSGMSQ

>HDC9780392.1 peptidylprolyl isomerase [Staphylococcus aureus]

MKMINKLIVPVTASALLLGACGASATDSKENTLISSKAGDVTVADTMKKIGKDQIANASFTEMLNKILADKYKNKVNDKKIDEQIEKMQKQYGGKDKFKKALQQQGLTADKYKENLRTAAYHKELLSDKIKISDSEIKEDSKKASHILIKVKSKKSDKEGLDDKEAKQKAEEIQKEVSKDPSKFGEIAKKESMDTGSAKKDGELGYVLKGQTDKDFEKALFKLKDGEVSDVVKSSFGYHIIKADKPTDFNSEKQSLKEKIVDQKVQKNPKLLTDAYKDLLKEYDVDFKDRDIKSVVEDKILNPEKLKQGGAQGGQSGMSQ

>WP_250723057.1 peptidylprolyl isomerase [Staphylococcus aureus]

MKMINKLIVPVTASALLLGACGASATDSKENTLISSKAGDVTVADTMKKIGKDQIANASFTEMLNKILADKYKNKVNDKKIDEQIEKMQKQYGGKDKFEKALQQQGLTADKYKENLRTAAYHKELLSDKIKISDSEIKEDSKKASHILIKVKSKKSDKVGLDDKEAKQKAEEIQKEVSKDPSKFGEIAKKESMDTGSAKKDGELGYVLKGQTDKDFEKALFKLKDGEVSDVVKSSFGYHIIKADKPTDFNSEKQSLKEKLVDQKVQKNPKLLTDAYKDLLKEYDVDFKDRDIKSVVEDKILNPEKLKQGGAQGGQSGMSQ

>HDA7625930.1 peptidylprolyl isomerase [Staphylococcus aureus]

MKMINKLIVPVTASALLLGACGTSATDSKENTLISSKAGDVTVADTMKKIGKDQIANASFTEMLNKILADKYKNKVNDKKIDEQIEKMQKQYGGKDKFEKALQQQGLTADKYKENLRTAAYHKELLSDKIKISDSEIKEDSKKASHILIKVKSKKSDKEGLDDKEAKQKAEEIQKEVSKDPSKFGEIAKKESMDTGSAKKDGELGYVLKGQTDKDFEKALFKLKDGEVSDVVKSSFGYHIIKADKPTDFNSEKQSLKEKLVDQKVQKNPKLLTDAYKDLLKEYDVDFKDRDIKSVVEDKILNPEKLKQGGAQGGQSGMSQ

>HDD7326239.1 peptidylprolyl isomerase [Staphylococcus aureus]

MKMINKLIVPVTASALLLGACGASATDSKENTLISSKAGDVTVADTMKKIGKDQIANASFTEMLNKILADKYKNKVNDKKIDEQIEKMQKQYGGKDKFEKALQQQGLTADKYKENLRTAAYHKELLSDKIKISDSEIKEDSKKASHILIKVKSKKSDKEGLDDKEAKQKAEEIQKEVSKDPSKFGEIAKKESMDTGSAKKDGELGYVLKGQTDKDFEKTLFKLKDGEVSDVVKSSFGYHIIKADKPTDFNSEKQSLKEKLVDQKVQKNPKLLTDAYKDLLKEYDVDFKDRDIKSVVEDKILNPEKLKQGGAQGGQSGMSQ

>WP_248491320.1 foldase protein PrsA [Staphylococcus aureus]

MKMINKLIVPVTASALLLGACGASATDSKENTLISSKAGDVTVADTMKKIGKDQIANASFTEMLNKILADKYKNKVNDKKIDEQIEKMQKQYGGKDKFEKALQQQGLTADKYKENLRTAAYHKELLSDKIKISDSEIKEDSKKASHILIKVKSKKSDKEGLDDKEAKQKAEEIQKEVSKDPSKFGEITKKESMDTGSAKKDGELGYVLKGQTDKDFEKALFKLKDGEVSDVVKSSFGYHIIKADKPTDFNSEKQSLKEKLVDQKVQKNPKLLTDAYKDLLKEYDVDFKDRDIKSVVEDKILNPEKLKQGGAQGGQSGMSQ

>EKF1434698.1 peptidylprolyl isomerase [Staphylococcus aureus]

MKMINKLIVPVTASTLLLGACGASATDSKENTLISSKAGDVTVADTMKKIGKDQIANASFTEMLNKILADKYKNKVNDKKIDEQIEKMQKQYGGKDKFEKALQQQGLTADKYKENLRTAAYHKELLSDKIKISDSEIKEDSKKASHILIKVKSKKSDKEGLDDKEAKQKAEEIQKEVSKDPSKFGEIAKKESMDTGSAKKDGELGYVLKGQTDKDFEKALFKLKDGEVSDVVKSSFGYHIIKADKPTDFNSEKQSLKEKLVDQKVQKNPKLLTDAYKDLLKEYDVDFKDRDIKSVVEDKILNPEKLKQGGAQGGQSGMSQ

>HCT1640714.1 peptidylprolyl isomerase [Staphylococcus aureus]

MKMINKLIVPVTASALLLGACGASATDSKENTLISSKAGDVTVADTMKKIGKDQIANASFTEMLNKILADKYKNKVNDKKIDEQIEKMQKQYGGKDKFKKALQQQGLTADKYKENLRTAAYHKELLSDKIKISDSEIKEDSKKASHILIKVKSKKSDKEGLDDKEAKQKAEEIQKEVSKDPSKFGEIAKKESMDTGSAKKDGELGYVLKGQTDKDFEKALFKLKDGEVSDVVKSSFGYHIIKADKPTDFNSEKQSLKEKLVDQKVQKNPKLLTDAYKDLLKEYDVDFKDRDIKSVVEDKILNPEKLKQGGAQGGQSG

>HCY6114293.1 peptidylprolyl isomerase [Staphylococcus aureus]

MKMINKLIVPVTASALLLGACGASATDSKENTLISSKAGDVTVADTMKKIGKDQIANASFTEMLNKILADKYKNKVNDKKIDEQIEKMQKQYGDKDKFKKALQQQGLTADKYKENLRTAAYHKELLSDKIKISDSEIKEDSKKASHILIKVKSKKSDKEGLDDKEAKQKAEEIQKEVSKDPSKFGEIAKKESMDTGSAKKDGELGYVLKGQTDKDFEKALFKLKDGEVSDVVKSSFGYHIIKADKPTDFNSEKQSLKEKLVDQKVQKNPKLLTDAYKDLLKEYDVDFKDRDIKSVVEDKILNPEKLKQGGAQGGQSGMSQ

>WP_208151559.1 foldase protein PrsA [Staphylococcus aureus]

MKMINKLIVPVTASALLLGACGASATDSKENTLISSKAGDVIVADTMKKIGKDQIANASFTEMLNKILADKYKNKVNDKKIDEQIEKMQKQYGGKDKFEKALQQQGLTADKYKENLRTAAYHKELLSDKIKISDSEIKEDSKKASHILIKVKSKKSDKEGLDDKEAKQKAEEIQKEVSKDPSKFGEIAKKESMDTGSAKKDGELGYVLKGQTDKDFEKALFKLKDGEVSDVVKSSFGYHIIKADKPTDFNSEKQSLKEKLVDQKVQKNPKLLTDAYKDLLKEYDVDFKDRDIKSVVEDKILNPEKLKQGGAQGGQSGMSQ

>HDB8478118.1 peptidylprolyl isomerase [Staphylococcus aureus]

MKMINKLIVPVTASALLLGACGASATDSKENTLISSKAGDVTVADTMKKIGKDQIANASFTEMLNKILADKYKNKVNDKKIDEQIEKMQKQYGGKDKFEKALQQQGLTADKYKENLRTVAYHKELLSDKIKISDSEIKEDSKKASHILIKVKSKKSDKEGLDDKEAKQKAEEIQKEVSKNPSKFGEIAKKESMDTGSAKKDGELGYVLKGQTDKDFEKALFKLKDGEVSDVVKSSFGYHIIKADKPTDFNSEKQSLKEKLVDQKVQKNPKLLTDAYKDLLKEYDVDFKDRDIKSVVEDKILNPEKLKQGGAQGGQSGMSQ

>HCD8543591.1 peptidylprolyl isomerase [Staphylococcus aureus]

MKMINKLIVPVTASALLLGACGASATDSKENTLISSKAGDVTVADTMKKIGKDQIANASFTKMLNKILADKYKNKVNDKKIDEQIEKMQKQYGGKDKFEKALQQQGLTADKYKENLRTAAYHKELLSDKIKISDSEIKEDSKKASHILIKVKSKKSDKEGLDDKEAKQKAEEIQKEVSKNPSKFGEIAKKESMDTGSAKKDGELGYVLKGQTDKDFEKALFKLKDGEVSDVVKSSFGYHIIKADKPTDFNSEKQSLKEKLVDQKVQKNPKLLTDAYKDLLKEYDVDFKDRDIKSVVEDKILNPEKLKQGGAQGGQSGMSQ

>HCU0734934.1 peptidylprolyl isomerase [Staphylococcus aureus]

MKMINKLIVPVTASALLLGACGASATDSKENTLISSKAGDVTVADTMKKIGKDQIANASFTEMLNKILADKYKNKVNDKKIDEQIEKMQKQYGGKDKFKKALQQQGLTADKYKENLRTAAYHKELLSDKIKISDSEIKEDSKKASHILIKVKSKKSDKEGLDDKEAKQKAEEIQKEVSKDPSKFGEIAKKESMDTGSAKKDGELGYVLKGQTDKDFEKALFKLKDGEVSDVVKSSFGYHIIKADKPTDFNSEKQSLKEKLVDQKVQKNPKLLTDAYKDLLKEYDVDFKDRDIKSVVEDKILNPEKLKQGGAQGGQSGM

>HED5907456.1 peptidylprolyl isomerase [Staphylococcus aureus]

MKMINKLIVPVTASALLLGACGASATDSKENTLISSKAGDVTVADTMKKIGKDQIANASFTEMLNKILADKYKNKVNDKKIDEQIEKMQKQYGGKDKFEKALQQQGLTADKYKENLRTAAYHKELLSDKIKISDSEIKEDSKKASHILIKVKSKKSDKEGLDDKEAKQKAEEIQKEVSKDPSKFGEIAKKESMDTGSAKKDGELGYVLKGQTDKDFEKALFKLKDGEVSDVVKSSFGYHIIKADKPTDFNSEKQ

>HAR4490732.1 peptidylprolyl isomerase [Staphylococcus aureus]

MKMINKLIVPVTASALLLGACGASATDSKENTLISSKAGDVTVADTMKKIGKDQIANASFTEMLNKILADKYKNKVNDKKSDEQIEKMQKQYGGKDKFEKALQQQGLTADKYKENLRTAAYHKELLSDKIKISDSEIKEDSKKASHILIKVKSKKSDKEGLDDKEAKQKAEEIQKEVSKDPSKFGEIAKKESMDTGSAKKDGELGYVLKGQTDKDFEKALFKLKDGEVSDVVKSSFGYHIIKADKPTDFNSEKQSLKEKLVDQKVQKNPKLLTDAYKDLLKEYDVDFKDRDIKSVVEDKILNPEKLKQGGAQGGQSGMSQ

>HCY8745735.1 peptidylprolyl isomerase [Staphylococcus aureus]

MKMINKLIVPVTASALLLGACGASATDSKENTLISSKAGDVTVADTMKKIGKDQIANASFTEMLNKILADKYKNKVNDKKIDEQIEKMQKQYGGKDKFKKALQQQGLTADKYKENLRTAAYHKELLSDKIKISDSEIKEDSKKASHILIKVKSKKSDKEGLDDKEAKQKAEEIQKEVSKDPSKFGEIAKKESMDTGSAKKDGELGYVLKGQTDKDFEKALFKLKDGEVSDVVKSSFGYHIIKADKPTDFNSEKQSLKEKLVDQKVQKNPKLLTDAYKDLLKEYDVDFKDRDIKSVVEDKILNPEKLKQGGAQGGQSA

>HCY6125351.1 peptidylprolyl isomerase [Staphylococcus aureus]

MKMINKLIVPVTASALLLGACGASATDSKENTLISSKAGDVTVADTMKKIGKDQIANASFTEMLNKILADKYKNKVNDKKIDEQIEKMQKQYGGKDKFKKALQQQGLTADKYKENLRTAAYHKELLSDKIKISDSEIKEDSKKASHILIKVKSKKSDKEGLDDKEAKQKAEEIQKEVSKDPSKFGEIAKKESMDTGSAKKDGELGYVLKGQTDKDFEKALFKLKDGEVSDVVKSSFGYHIIKADKPTDFNSEKQSLKEKLVDQKVQKNPKLLTDAYKDLLKEYDVDFKDRDIKSVVEDKILNPEKLKQGGAQGGQSGMNQ

>HDF4860665.1 peptidylprolyl isomerase [Staphylococcus aureus]

MKMINKLIVPVTASALLLGACGASATDSKENTLISSKAGDVTVADTMKKIGKDQIANASFTEMLNKILADKYKNKVNDKKIDEQIEKMQKQYGGKDKFKKALQQQGLTADKYKENLRTAAYHKELLSDKIKISDSEIKEDSKKASHILIKVKSKKSDKEGLDDKEAKQKAEEIQKEVSKDPSKFGEIAKKESMDTGSAKKDGELDYVLKGQTDKDFEKALFKLKDGEVSDVVKSSFGYHIIKADKPTDFNSEKQSLKEKLVDQKVQKNPKLLTDAYKDLLKEYDVDFKDRDIKSVVEDKILNPEKLKQGGAQGGQSGMSQ

>HCY1355694.1 peptidylprolyl isomerase [Staphylococcus aureus]

MKMINKLIVPVTASALLLGACGASATDSKENTLISSKAGDVTVADTMKKIGKDQIANASFTEMLNKILADKYKNKVNDKKIDEQIEKMQKQYGGKDKFKKALQQQGLTADKYKENLRTAAYHKELLSDKIKISDSEIKEDSKKASHILIKVKSKKSDKEGLDDKEAKQKAEEIQKEVSKDPSKFGEIAKKESMDTGSAKKDGELGYVLKGQTDKDFEKALFKLKDGEVSDVVKSSFGYHIIKADKPTDFNSEKQSLKEKLVDQKVQKNPKLLTDAYKDLLKEYDVDFKDRDIKSVVEDKILNPEKLKQGGAQGGQSVMSQ

>HDC8324604.1 peptidylprolyl isomerase [Staphylococcus aureus]

MKMINKLIVPVTASALLLGACGASATDSKENTLISSKAGDVTVADTMKKIGKDQIANASFTEMLNKILADKYKNKVNDKKIDEQIEKMQKQYGGKDKFEKALQQQGLTADKYKENLRTAAYHKELLSDKIKISDSEIKEDSKKASHILIKVKSKKSDKEGLDDKEAKQKAEEIQKEVSKNPSKFGEIAKKESMDTGSAKKDGELGYVLKGQTDKDFEKALFKLKDGEVSDVVKSSFGYHIIKADKPTDFNSEKQSLKEKLVDQKVQKNPKLLTDAYKDLLKEYDVDFKDRDIKSVVEDKILNPEKLKQGGAKGGQSGMSQ

>EKK3610689.1 peptidylprolyl isomerase [Staphylococcus aureus]

MKMINKLIVPVTASALLLGACGASATDSKENTLISSKAGDVTVADTMKKIGKDQIANASFTEMLNKILADKYKNKVNDKKIDEQIEKMQKQYGGKDKFKKALQQQGLTADKYKENLRTAAYHKELLSDKIKISDSEIKEDSKKASHILIKVKSKKSDKEGLDDKEAKQKAEEIQKEVSKDPSKFGEIAKKESMDTGSAKKDGELGYVLKGQTDKDFEKALFKLKDGEVSDVVKSSFGYHIIKADKPTDFNSEKQSLKEKLVDQKVQKNPKLLTDAYKDILKEYDVDFKDRDIKSVVEDKILNPEKLKQGGAQGGQSGMSQ

>WP_064138623.1 peptidylprolyl isomerase [Staphylococcus aureus]

MKMINKLIVPVTASALLLGACGASATDSKENTLISSKAGDVTVADTMKKIGKDQIANASFTEMLNKILADKYKNKVNDKKIDEQIEKMQKQYGGKDKFEKALQQQGLTADKYKENLRTAAYHKELLSDKIKISDSEIKEDSKKASHILIKVKSKKSDKKGLDDKEAKQKAEEIQKEVSKDPSKFGEIAKKESMDTGSAKKDGELGYVLKGQTDKDFEKALFKLKDGEVSDVVKSSFGYHIIKADKPTDFNSEKQSLKEKLVDQKVQKNPKLLTDAYKDLLKEYDVDFKDRDIKSVVEDKILNPEKLKQGGAKGGQSGMSQ

>HCY8173453.1 peptidylprolyl isomerase [Staphylococcus aureus]

MKMINKLIVPVTASALLLGACGASATDSKENTLISSKAGDVTVADTMKKIGKDQIANASFTEMLNKILADKYKNKVNDKKIDEQIEKMQKQYGGKDKFKKALQQQGLTADKYKENLRTAAYHKELLSDKIKISDSEIKEDSKKASHILIKVKSKKSDKEGLDDKEAKQKAEEIQKEVSKDPSKFGEIAKKESMDTGSAKKDGELGYVLKGQTDKDFEKALFKLKDGEVSDVVKSSFGYHIIKADKPTDFNSEKQSLKEKLVDQKVQKNPKLLTDAYKDLLKEYDVDFKDRDIKSVVEDKILNPEKLKQGGAQGGQSAMSQ

>WP_228580537.1 foldase protein PrsA [Staphylococcus aureus]

MKMINKLIVPVTASALLLGACGASATDSKENTLISSKAGDVTVADTMKKIGKDQIANASFTEMLNKILADKYKNKVNDKKIDEQIEKMQKQYGGKDKFKKALQQQGLTADKYKENLRTAAYHKELLSDKIKISDSEIKEDSKKASHILIKVKSKKSDKEGLDDKEAKQKAEEIQKEVSKDPSKFGEIAKKESMDTGSAKKDGELGYVLKGQTDKDFEKALFKLKDGEVSDVVKSSFGYHIIKADKPTDFNSEKQSLKEKLVDQKVQKNPKLLTDAYKDLLKEYDVDFKDRDIKSVVEDKILNPEKLKHGGAQGGQSGMSQ

>EHM86697.1 putative foldase protein PrsA, partial [Staphylococcus aureus subsp. aureus 21194]

TASALLLGACGASATDSKENTLISSKAGDVTVADTMKKIGKDQIANASFTEMLNKILADKYKNKVNDKKIDEQIEKMQKQYGGKDKFEKALQQQGLTADKYKENLRTAAYHKELLSDKIKISDSEIKEDSKKASHILIKVKSKKSDKEGLDDKEAKQKAEEIQKEVSKDPSKFGEIAKKESMDTGSAKKDGELGYVLKGQTDKDFEKALFKLKDGEVSDVVKSSFGYHIIKADKPTDFNSEKQSLKEKLVDQKVQKNPKLLTDAYKDLLKEYDVDFKDRDIKSVVEDKILNPEKLKQGGAQGGQSGMSQ

>HBI8395845.1 peptidylprolyl isomerase [Staphylococcus aureus]

MKMINKLIVPVTASALLLGACGASATDSKENTLISSKAGDVTVADTMKKIGKDQIANASFTEMLNKILADKYKNKVNDKKIDEQIEKMQKQYGGKDKFKKALQQQGLTADKYKENLRTAAYHKELLSDKIKISDSEIKEDSKKASHILIKVKSKKSDKEGLDDKEAKQKAEEIQKEVSKDPSKFGEIAKKESMDTGSAKKDGELGYVLKGQTDKDFEKALFKLKDGEVSDVVKSSFGYHIIKADKPTDFNSEKQSLKEKLVDQKVQKNPKLLTDAYKDLLKEYDIDFKDRDIKSVVEDKILNPEKLKQGGAQGGQSGMSQ

>HAY2606533.1 peptidylprolyl isomerase [Staphylococcus aureus]

MKMINKLIVPVTASALLLGACGASATDSKENTLISSKAGDVTVADTMKKIGKDQIANASFTEMLNKILADKYKNKVNDKKIDEQIEKMQKQYGGKDKFEKALQQQGLTADKYKENLRTAAYHKELLSDKIKISDSEIKEDSKKASHILIKVKSKKSDKEGLDDKEAKQKAEEIQKEVSKDPSKFGEIAKKESMDTGSAKKDGELGYVLKGQTDKDFEKALFKLKDGEVSDVVKSSFGYHIIKADKPTDFNSEKQSLKEKLVDQKVQKKPKLLTDAYKDLLKEYDVDFKDRDIKSVVEDKILNPEKLKQGGAQGGQSGMSQ

>HAR7394520.1 peptidylprolyl isomerase [Staphylococcus aureus]

MKMINKLIVPVTASALLLGACGASATDSKENTLISSKAGDVTVADTMKKIGKDQIANASFTEMLNKILADKYKNKVNDKKIDEQIEKMQKQYGGKDKFKKALQQQGLTADKYKENLRTAAYHKELLSDKIKISDSEIKEDSKKASHILIKVKSKKSDKEGLDDKEAKQKAEEIQKEVSKDPSKFGEIAKKESMDTGSAKKDGELGYVLKGQTDKNFEKALFKLKDGEVSDVVKSSFGYHIIKADKPTDFNSEKQSLKEKLVDQKVQKNPKLLTDAYKDLLKEYDVDFKDRDIKSVVEDKILNPEKLKQGGAQGGQSGMSQ

>WP_303751533.1 foldase protein PrsA [Staphylococcus aureus]

MKMINKLIVPVTASALLLGACGASATDSKENTLISSKAGDVTVADTMKKIGKDQIANASFTEMLNKILADKYKNKVNDKKIDEQIEKMQKQYGGKDKFKKALQQQGLTADKYKENLRTAAYHKELLSDKIKISDSEIKEDSKKASHILIKVKSKKSDKEGLDDKEAKQKAEEIQKEVSKDPSKFGEIAKKESMDTGSAKKDGELGYVLKGQTDKDFEKALFKLKDGEVSDVVKSSFGYHIIKADKPTDFNSEKQSLKEKLVDQKVQKNPKLLTDAYKNLLKEYDVDFKDRDIKSVVEDKILNPEKLKQGGAQGGQSGMSQ

>HBC4873437.1 peptidylprolyl isomerase [Staphylococcus aureus]

MKMINKLIVPVTASALLLGACGASATDSKENTLISSKAGDVTVADTMKKIGKDQIGNASFTEMLNKILADKYKNKVNDKKIDEQIEKMQKQYGGKDKFKKALQQQGLTADKYKENLRTAAYHKELLSDKIKISDSEIKEDSKKASHILIKVKSKKSDKEGLDDKEAKQKAEEIQKEVSKDPSKFGEIAKKESMDTGSAKKDGELGYVLKGQTDKDFEKALFKLKDGEVSDVVKSSFGYHIIKADKPTDFNSEKQSLKEKLVDQKVQKNPKLLTDAYKDLLKEYDVDFKDRDIKSVVEDKILNPEKLKQGGAQGGQSGMSQ

>HDA1721162.1 peptidylprolyl isomerase [Staphylococcus aureus]

MKMINKLIVPVTASALLLGACGASATDSKENTLISSKAGDVKVADTMKKIGKDQIANASFTEMLNKILADKYKNKVNDKKIDEQIEKMQKQYGGKDKFEKALQQQGLTADKYKENLRTAAYHKELLSDKIKISDSEIKEDSKKASHILIKVKSKKSDKEGLDDKEAKQKAEEIQKEVSKDPSKFGEIAKKESMDTGSAKKDGELGYVLKGQTDKDFEKALFKLKDGEVSDVVKSSFGYHIIKADKPTDFNSEKQSLKEKLVDQKVQKNPKLLTDAYKDLLKEYDVDFKDRDIKSVVEDKILNPEKLKQGGAQGGQSGMSQ

>WP_064134305.1 peptidylprolyl isomerase, partial [Staphylococcus aureus]

MKMINKLIVPVTASALLLGACGASATDSKENTLISSKAGDVTVADTMKKIGKDQIANASFTEMLNKILADKYKNKVNDKKIDEQIEKMQKQYGGKDKFKKALQQQGLTADKYKENLRTAAYHKELLSDKIKISDSEIKEDSKKASHILIKVKSKKSDKEGLDDKEAKQKAEEIQKEVSKDPSKFGEIAKKESMDTGSAKKDGELGYVLKGQTDKDFEKALFKLKDGEVSDVVKSSFGYHIIKADKPTDFNSEKQSLKEKLVDQKVQKNP

>HDI0763899.1 peptidylprolyl isomerase [Staphylococcus aureus]

MKMINKLIVPVTASALLLGACGASATDSKENTLISSKAGDVTVADTMKKIGKDQIANASFTEMLNKILADKYKNKVNDKKIDEQIEKMQKQYGGKDKFKKALQQQGLTADKYKENLRTASYHKELLSDKIKISDSEIKEDSKKASHILIKVKSKKSDKEGLDDKEAKQKAEEIQKEVSKDPSKFGEIAKKESMDTGSAKKDGELGYVLKGQTDKDFEKALFKLKDGEVSDVVKSSFGYHIIKADKPTDFNSEKQSLKEKLVDQKVQKNPKLLTDAYKDLLKEYDVDFKDRDIKSVVEDKILNPEKLKQGGAQGGQSGMSQ

>WP_208689384.1 foldase protein PrsA [Staphylococcus aureus]

MKMINKLIVPVTASALLLGACGASATDSKENTLISSKAGDVTVADTMKKIGKDQIANASFTEMLNKILADKYKNKVNDKKNDEQIEKMQKQYGGKDKFEKALQQQGLTADKYKENLRTAAYHKELLSDKIKISDSEIKEDSKKASHILIKVKSKKSDKEGLDDKEAKQKAEEIQKEVSKDPSKFGEIAKKESMDTGSAKKDGELGYVLKGQTDKDFEKALFKLKDGEVSDVVKSSFGYHIIKADKPTDFNSEKQSLKEKLVDQKVQKNPKLLTDAYKDLLKEYDVDFKDRDIKSVVEDKILNPEKLKQGGAQGGQSGMSQ

>HDE4517710.1 peptidylprolyl isomerase [Staphylococcus aureus]

MKMINKLIVPVTASALLLGACGASATDSKENTLISSKAGDVTVADTMKKIGKDQIANASFTEMLNKILADKYKNKVNDKKIDEQIEKMQKQYGGKDKFKKALQQQGLTADKYKENLRTAAYHKELLSDKIKISDSEIKEDSKKASHILIKVKSKKSDKEGLDDKEAKQKAEGIQKEVSKDPSKFGEIAKKESMDTGSAKKDGELGYVLKGQTDKDFEKALFKLKDGEVSDVVKSSFGYHIIKADKPTDFNSEKQSLKEKLVDQKVQKNPKLLTDAYKDLLKEYDVDFKDRDIKSVVEDKILNPEKLKQGGAQGGQSGMSQ

>HEA6576722.1 peptidylprolyl isomerase [Staphylococcus aureus]

MKMINKLIVPVTASALLLGACGASATDSKENTLISSKAGDVTVADTMKKIGKDQIANASFTEMLNKILADKYKNKVNDKKIDERIEKMQKQYGGKDKFKKALQQQGLTADKYKENLRTAAYHKELLSDKIKISDSEIKEDSKKASHILIKVKSKKSDKEGLDDKEAKQKAEEIQKEVSKDPSKFGEIAKKESMDTGSAKKDGELGYVLKGQTDKDFEKALFKLKDGEVSDVVKSSFGYHIIKADKPTDFNSEKQSLKEKIVDQKVQKNPKLLTDAYKDLLKEYDVDFKDRDIKSVVEDKILNPEKLKQGGAQGGQSGMSQ

>HDI8056162.1 peptidylprolyl isomerase [Staphylococcus aureus]

MKMINKLIVPVTASALLLGACGASATDSKENTLISSKAGDVTVADTMKKIGKDQIANASFTEMLNKILADKYKNKVNDKKIDEQIEKMQKQYGGKDKFKKALQQQGLTADKYKENLRTAAYHKELLSDKIKISDSEIKEDSKKASHILIKVKSKKSDKEGLDDKEAKQKAEEIQKEVSKDPSKFGEIAKKESMDTGSAKKDGELGYVLKGQTDKDFEKALFKLKDGEVSDVVKSSFGYHIIKADKPTDFNSEKQSLKEKLVDQKVQKNPKLLTDAYKDLLKEYDVDFKDRDTKSVVEDKILNPEKLKQGGAQGGQSGMSQ

>WP_315976465.1 peptidylprolyl isomerase [Staphylococcus aureus]

MKMINKLIVPVTASALLLGACGASATDSKENTLISSKAGDVTVADTMKKIGKDQIANASFTEMLNKILADKYKNKVNDKKIDEQIEKMQKQYGGKGKFEKALQQQGLTADKYKENLRTAAYHKELLSDKIKISDSEIKEDSKKASHILIKVKSKKSDKEGLDDKEAKQKAEEIQKEVSKDPSKFGEIAKKESMDTGSAKKDGELGYVLKGQTDKDFEKALFKLKDGEVSDVVKSSFGYHIIKADKPTDFNSEKQSLKEKLVDQKVQKNPKLLTDAYKDLLKEYDVDFKDRDIKSVVEDKILNPEKLKQGGAQGGQSGMSQ

>HDB4375794.1 peptidylprolyl isomerase [Staphylococcus aureus]

MKMINKLIVPVTASALLLGACGASATDSKENTLISSKAGDVTVADTMKKIGKDQIANASFTEMLNKILADKYKNKVNDKKIDEQIEKMQKQYGGKDKFKKALQQQGLTADKYKENLRTAAYHKELLSDKIKISDSEIKEDSKKASHILIKVKSKKSDKEGLDDKEAKQKAEEIQKEVSKDPSKFVEIAKKESMDTGSAKKDGELGYVLKGQTDKDFEKALFKLKDGEVSDVVKSSFGYHIIKADKPTDFNSEKQSLKEKLVDQKVQKNPKLLTDAYKDLLKEYDVDFKDRDIKSVVEDKILNPEKLKQGGAQGGQSGMSQ

>HAR7054974.1 peptidylprolyl isomerase [Staphylococcus aureus]

MKMINKLIVPVTASALLLGACGASATDSKENTLISSKAGDVTVADTMKKIGKDQIANASFTEMLNKILADKYKNKVNDKKIDEQIEKMQKQYGGKDKFEKALQQQGLTADKYKENLRTAAYHKELLSDKIKISDSEIKEDSKKASHILIKVKSKKSDKEGLDDKEAKQKAEEIQKEVSKDPSKFGEIAKKESMDTGSAKKDGELGYVLKGQTDKDFEKALFKLKDGEVSDVVKSSFGYHIIKADKPTDFNSEKQSPKEKLVDQKVQKNPKLLTDAYKDLLKEYDVDFKDRDIKSVVEDKILNPEKLKQGGAQGGQSGMSQ

>HCV0019218.1 peptidylprolyl isomerase [Staphylococcus aureus]

MKMINKLIVPVTASALLLGACGASATDSKENTLISSKAGDVTVADTMKKIGKDQIANASFTEMLNKILADKYKNKVNDKKIDEQIEKMQKQYGGKDKFKKALQQQGLTADKYKENLRTAAYHKELLSDKIKISDSEIKEDSKKASHILIKVKSKKSDKEGLDDKEAKQKAEEIQKEVSKDPSKFGEIAKKESMDTGSAKKDGELGYVLKGQTDKDFEKALFKLKDGEVSDVVKSRFGYHIIKADKPTDFNSEKQSLKEKLVDQKVQKNPKLLTDAYKDLLKEYDVDFKDRDIKSVVEDKILNPEKLKQGGAQGGQSGMSQ

>HCU8047529.1 peptidylprolyl isomerase [Staphylococcus aureus]

MKMINKLIVPVTASALLLGACGASATDSKENTLISSKAGDVTVADTMKKIGKDQIANASFTEMLNKILADKYKNKVNDKKIDEQIEKMQKQYSGKDKFEKALQQQGLTADKYKENLRTAAYHKELLSDKIKISDSEIKEDSKKASHILIKVKSKKSDKEGLDDKEAKQKAEEIQKEVSKDPSKFGEIAKKESMDTGSAKKDGELGYVLKGQTDKDFEKALFKLKDGEVSDVVKSSFGYHIIKADKPTDFNSEKQSLKEKLVDQKVQKNPKLLTDAYKDLLKEYDVDFKDRDIKSVVEDKILNPEKLKQGGAQGGQSGMSQ

>QOJ77649.1 peptidylprolyl isomerase [Staphylococcus aureus]

MKMINKLIVPVTASALLLGACGASATDSKENTLISSKAGDVTVADTMKKIGKDQIAKASFTEMLNKILADKYKNKVNDKKIDEQIEKMQKQYGGKDKFEKALQQQGLTADKYKENLRTAAYHKELLSDKIKISDSEIKEDSKKASHILIKVKSKKSDKEGLDDKEAKQKAEEIQKEVSKDPSKFGEIAKKESMDTGSAKKDGELGYVLKGQTDKDFEKALFKLKDGEVSDVVKSSFGYHIIKADKPTDFNSEKQSLKEKLVDQKVQKNPKLLTDAYKDLLKEYDVDFKDRDIKSVVEDKILNPEKLKQGGAQGGQSGMSQ

>HCY8110974.1 peptidylprolyl isomerase [Staphylococcus aureus]

MKMINKLIVPVTASALLLGACGASATDSKENTLISSKAGDVTVADTMKKIGKDQIANASFTEMLNKILADKYKNKVNDKKIDEQIEKMQKQYGGKDKFKKALQQQGLTADKYKENLRTAAYHKELLSDKIKISDSEIKEDSKKASHILIKVKSKKSDKEGLDDKEAKQKAEEIQKEVSKDPSKFGEIAKKGSMDTGSAKKDGELGYVLKGQTDKDFEKALFKLKDGEVSDVVKSSFGYHIIKADKPTDFNSEKQSLKEKLVDQKVQKNPKLLTDAYKDLLKEYDVDFKDRDIKSVVEDKILNPEKLKQGGAQGGQSGMSQ

>HDG8500468.1 peptidylprolyl isomerase [Staphylococcus aureus]

MKMINKLIVPVTASALLLGACGASATDSKENTLISSKAGDVTVADTMKKIGKDQIANASFTEMLNKILADKYKNKVNDKKIDEQIEKMQKQYGGKDKFEKALQQQGLTADKYKDNLRTAAYHKELMADKIKISDSEIKEDSKKASHILIKVKSKKSDKEGLDDKEAKQKAEEIQKEVSKDPSKFGEIAKKESMDTGSAKKDGELGYVLKGQTDKDFEKALFKLKDGEVSDVVKSSFGYHIIKADKPTDFNSEKQSLKEKLVDQKVQKNPKLLTDAYKDLLKEYDVDFKDRDIKSVVEDKILNPEKLKQGGAQGGQSGMSQ

>HCW7408006.1 peptidylprolyl isomerase [Staphylococcus aureus]

MKMINKLIVPVTASALLLGACGASATDSKENTLISSKAGDVTVADTMKKIGKDQIANASFTEMLNKILADKYKNKVNDKKIDEQIEKMQKQYGGKDKFEKALQQQGLTADKYKGNLRTAAYHKELLSDKIKISDSEIKEDSKKASHILIKVKSKKSDKEGLDDKEAKQKAEEIQKEVSKDPSKFGEIAKKESMDTGSAKKDGELGYVLKGQTDKDFEKALFKLKDGEVSDVVKSSFGYHIIKADKPTDFNSEKQSLKEKLVDQKVQKNPKLLTDAYKDLLKEYDVDFKDRDIKSVVEDKILNPEKLKQGGAQGGQSGMSQ

>HCV8108383.1 peptidylprolyl isomerase [Staphylococcus aureus]

MKMINKLIVPVTASALLLGACGASATDSKENTLISSKAGDVTVADTMKKIGKDQIANASFTEMLNKILADKYKNKVNDKKIDEQIEKMQKQYGGKDKFEKALQQQGLTADKYKENLRTAAYHKELLSDKIKISDSEIKEDSKKASHILIKVKSKKSDKEGLDDKEAKQKAEEIQKEVSKDPSKFGEIAKKESMDTGSAKKDGELGYVLKGQTDKDFEKALFKLKDGEVSDVVKSSFGYHIIKADKPTDFNSEKQSPKEKLVDQKVQKNPKLLTDAYKDLLKEYDVDFKDRDIKSVVEDKILNPEKLKQGGAKGGQSGMSQ

>HDG5885713.1 peptidylprolyl isomerase [Staphylococcus aureus]

MKMINKLIVPVTASALLLGACGASATDSKENTLISSKAGDVTVADTMKKIGKDQIANASFTEMLNKILADKYKNKVNDKKIDEQIEKMQKQYGGKDKFEKALQQQALTADKYKENLRTAAYHKELLSDKIKISDSEIKEDSKKASHILIKVKSKKSDKEGLDDKEAKQKAEEIQKEVSKDPSKFGEIAKKESMDTGSAKKDGELGYVLKGQTDKDFEKALFKLKDGEVSDVVKSSFGYHIIKADKPTDFNSEKQSLKEKLVDQKVQKNPKLLTDAYKDLLKEYDVDFKDRDIKSVVEDKILNPEKLKQGGAKGGQSGMSQ

>WP_111053285.1 foldase protein PrsA [Staphylococcus aureus]

MKMINKLIVPVTASALLLGACGASATDSKENTLISSKAGDVTGADTMKKIGKDQIANASFTEMLNKILADKYKNKVNDKKIDEQIEKMQKQYGGKDKFEKALQQQGLTADKYKENLRTAAYHKELLSDKIKISDSEIKEDSKKASHILIKVKSKKSDKEGLDDKEAKQKAEEIQKEVSKDPSKFGEIAKKESMDTGSAKKDGELGYVLKGQTDKDFEKALFKLKDGEVSDVVKSSFGYHIIKADKPTDFNSEKQSLKEKLVDQKVQKNPKLLTDAYKDLLKEYDVDFKDRDIKSVVEDKILNPEKLKQGGAQGGQSGMSQ

>WP_191961489.1 foldase protein PrsA, partial [Staphylococcus aureus]

ALLLGACGASATDSKENTLISSKAGDVTVADTMKKIGKDQIANASFTEMLNKILADKYKNKVNDKKIDEQIEKMQKQYGGKDKFEKALQQQGLTADKYKENLRTAAYHKELLSDKIKISDSEIKEDSKKASHILIKVKSKKSDKEGLDDKEAKQKAEEIQKEVSKDPSKFGEIAKKESMDTGSAKKDGELGYVLKGQTDKDFEKALFKLKDGEVSDVVKSSFGYHIIKADKPTDFNSEKQSLKEKLVDQKVQKNPKLLTDAYKDLLKEYDVDFKDRDIKSVVEDKILNPEKLKQGGAQGGQSGMSQ

>HDA8007278.1 peptidylprolyl isomerase [Staphylococcus aureus]

MKMINKLIVPVTASALLLGACGASATDSKENTLISSKVGDVTVADTMKKIGKDQIANASFTEMLNKILADKYKNKVNDKKIDEQIEKMQKQYGGKDKFKKALQQQGLTADKYKENLRTAAYHKELLSDKIKISDSEIKEDSKKASHILIKVKSKKSDKEGLDDKEAKQKAEEIQKEVSKDPSKFGEIAKKESMDTGSAKKDGELGYVLKGQTDKDFEKALFKLKDGEVSDVVKSSFGYHIIKADKPTDFNSEKQSLKEKLVDQKVQKNPKLLTDAYKDLLKEYDVDFKDRDIKSVVEDKILNPEKLKQGGAQGGQSGMSQ

>HCZ0154229.1 peptidylprolyl isomerase [Staphylococcus aureus]

MKMINKLIVPVTASALLLGACGASATDSKENTLISSKAGDVTVADTMKKIGKDQIANASFTEMLNKILADKYKNKVNDKKIDEQIEKMQKQYGGKDKFKKALQQQGLTADKYKENLRTAAYHKELLSDKIKISDSEIKEDSKKASHILIKVKSKKSDKEGLDDKEAKQKAEEIQKEVSKDPSKFGEIAKKESMDTGSAKKDGELGYVLKGQTDKDFEKALFKLKDGEVSDVVKSSFGYHIIKADKPTDFNSEKQSLKEKLVDQIVQKNPKLLTDAYKDLLKEYDVDFKDRDIKSVVEDKILNPEKLKQGGAQGGQSGMSQ

>HDC7745260.1 peptidylprolyl isomerase [Staphylococcus aureus]

MKMINKLIVPVTASALLLGACGASATDSKENTLISSKARDVTVADTMKKIGKDQIANASFTEMLNKILADKYKNKVNDKKIDEQIEKMQKQYGGKDKFEKALQQQGLTADKYKENLRTAAYHKELLSDKIKISDSEIKEDSKKASHILIKVKSKKSDKEGLDDKEAKQKAEEIQKEVSKDPSKFGEIAKKESMDTGSAKKDGELGYVLKGQTDKDFEKALFKLKDGEVSDVVKSSFGYHIIKADKPTDFNSEKQSLKEKLVDQKVQKNPKLLTDAYKDLLKEYDVDFKDRDIKSVVEDKILNPEKLKQGGAQGGQSGMSQ

>HDB6105676.1 peptidylprolyl isomerase [Staphylococcus aureus]

MKMINKLIVPVTASALLLGACGASATDSKENTLISSKAGDVTVADTMKKIGKDQIANASFTEMLNKILADKYKNKVNDKKIDEQIEKMQKQYGGKDKFEKALQQQGLTADKYKENLRTAAYHKELLSDKIKISDSEIKEDSKKASHILIKVKSKKSDKEGLDDKEAKQKAEEIQKEVSKDPSKFGEIAKKESMDTGSAKKDGELGYVLKGQTDKDFEKALFKLKDREVSDVVKSSFGYHIIKADKPTDFNSEKQSLKEKLVDQKVQKNPKLLTDAYKDLLKEYDVDFKDRDIKSVVEDKILNPEKLKQGGAQGGQSGMSQ

>WP_150024271.1 foldase protein PrsA [Staphylococcus aureus]

MKMINKLIVPVTASALLLGACGSSATDSKENTLISSKAGDVTVADTMKKIGKDQIANASFTEMLNKILADKYKNKVNDKKIDEQIEKMQKQYGGKDKFKKALQQQGLTADKYKENLRTAAYHKELLSDKIKISDSEIKEDSKKASHILIKVKSKKSDKEGLDDKEAKQKAEEIQKEVSKDPSKFGEIAKKESMDTGSAKKDGELGYVLKGQTDKDFEKALFKLKDGEVSDVVKSSFGYHIIKADKPTDFNSEKQSLKEKLVDQKVQKNPKLLTDAYKDLLKEYDVDFKDRDIKSVVEDKILNPEKLKQGGAQGGQSGMSQ

>HEK6183326.1 peptidylprolyl isomerase [Staphylococcus aureus]

MKMINKLIVPVTASSLLLGACGASATDSKENTLISSKAGDVTVADTMKKIGKDQIANASFTEMLNKILADKYKNKVNDKKIDEQIEKMQKQYGGKDKFKKALQQQGLTADKYKENLRTAAYHKELLSDKIKISDSEIKEDSKKASHILIKVKSKKSDKEGLDDKEAKQKAEEIQKEVSKDPSKFGEIAKKESMDTGSAKKDGELGYVLKGQTDKDFEKALFKLKDGEVSDVVKSSFGYHIIKADKPTDFNSEKQSLKEKLVDQKVQKNPKLLTDAYKDLLKEYDVDFKDRDIKSVVEDKILNPEKLKQGGAQGGQSGMSQ

>HAY1921286.1 peptidylprolyl isomerase [Staphylococcus aureus]

MKMINKLIVPVTASALLLGACGASATDSKENTLISSKAGDVTVADTMKKIGKDQIANASFTEMLNKILADKYKNKVNDKKIDEQIEKMQKQYDGKDKFEKALQQQGLTADKYKENLRTAAYHKELLSDKIKISDSEIKEDSKKASHILIKVKSKKSDKEGLDDKEAKQKAEEIQKEVSKDPSKFGEIAKKESMDTGSAKKDGELGYVLKGQTDKDFEKALFKLKDGEVSDVVKSSFGYHIIKADKPTDFNSEKQSLKEKLVDQKVQKNPKLLTDAYKDLLKEYDVDFKDRDIKSVVEDKILNPEKLKQGGAQGGQSGMSQ

>HCV0433649.1 peptidylprolyl isomerase [Staphylococcus aureus]

MKMINKLIVPVTASALLLGACGASATDSKENTLISSKAGDVTVADTMKKIGEDQIANASFTEMLNKILADKYKNKVNDKKIDEQIEKMQKQYGGKDKFKKALQQQGLTADKYKENLRTAAYHKELLSDKIKISDSEIKEDSKKASHILIKVKSKKSDKEGLDDKEAKQKAEEIQKEVSKDPSKFGEIAKKESMDTGSAKKDGELGYVLKGQTDKDFEKALFKLKDGEVSDVVKSSFGYHIIKADKPTDFNSEKQSLKEKLVDQKVQKNPKLLTDAYKDLLKEYDVDFKDRDIKSVVEDKILNPEKLKQGGAQGGQSGMSQ

>MBV2618256.1 peptidylprolyl isomerase [Staphylococcus aureus]

MKMINKLIVPVTASALLLGACGASATDSKENTLISSKAGDVTVADTMKKIGKDQIANASFTEMLNKILADKYKNKVNDKKIDEQIEKMQKQYGGKDKFEKALQQQGLTADKYKENLRTAAYHKELLSDKIKISDSEIKEDSKKASHILIKVKSKKSDKEGLDDKEAKQKAEEIQKEVSKDPSKFGEIAKKDGELGYVLKGQTDKDFEKALFKLKDGEVSDVVKSSFGYHIIKADKPTDFNSEKQSLKEKLVDQKVQKNPKLLTDAYKDLLKEYDVDFKDRDIKSVVEDKILNPEKLKQGGAKGGQSGMSQ

>HCU9855595.1 peptidylprolyl isomerase [Staphylococcus aureus]

MKMINKLIVPVTASALLLGACGASATDSKENTLISSKAGDVTVADTMKKIGKDQIANASFTEMLNKILADKYKNKVNDKKIDEQIEKMQKQYGGKDKFEKALQQQGLTADKYKENLRTAAYHKELLSDKIKISYSEIKEDSKKASHILIKVKSKKSDKEGLDDKEAKQKAEEIQKEVSKDPSKFGEIAKKESMDTGSAKKDGELGYVLKGQTDKDFEKALFKLKDGEVSDVVKSSFGYHIIKADKPTDFNSEKQSLKEKLVDQKVQKNPKLLTDAYKDLLKEYDVDFKDRDIKSVVEDKILNPEKLKQGGAQGGQSGMSQ

>MBV2698477.1 peptidylprolyl isomerase [Staphylococcus aureus]

MKMINKLIVPVTAGALLLGACGASATDSKENTLISSKAGDVTVADTMKKIGKDQIANASFTEMLNKILADKYKNKVNDKKIDEQIEKMQKQYGGKDKFKKALQQQGLTADKYKENLRTAAYHKELLSDKIKISDSEIKEDSKKASHILIKVKSKKSDKEGLDDKEAKQKAEEIQKEVSKDPSKFGEIAKKESMDTGSAKKDGELGYVLKGQTDKDFEKALFKLKDGEVSDVVKSSFGYHIIKADKPTDFNSEKQSLKEKLVDQKVQKNPKLLTDAYKDLLKEYDVDFKDRDIKSVVEDKILNPEKLKQGGAQGGQSGMSQ

>HDB6514199.1 peptidylprolyl isomerase [Staphylococcus aureus]

MKMINKLIVPVTASALLLGACGASATDSKENTLISSKAGDVTVADTMKKIGKDQIANASFTEMLNKILADKYKNKVNDKKIDEQIEKMQKQYGGKDKFEKALQQQDLTADKYKENLRTAAYHKELLSDKIKISDSEIKEDSKKASHILIKVKSKKSDKEGLDDKEAKQKAEEIQKEVSKDPSKFGEIAKKESMDTGSAKKDGELGYVLKGQTDKDFEKALFKLKDGEVSDVVKSSFGYHIIKADKPTDFNSEKQSLKEKLVDQKVQKNPKLLTDAYKDLLKEYDVDFKDRDIKSVVEDKILNPEKLKQGGAQGGQSGMSQ

>HBI0874937.1 peptidylprolyl isomerase [Staphylococcus aureus]

MKMINKLIVPVTASALLLGACGASATDSKENTLISSKAEDVTVADTMKKIGKDQIANASFTEMLNKILADKYKNKVNDKKIDEQIEKMQKQYGGKDKFEKALQQQGLTADKYKENLRTAAYHKELLSDKIKISDSEIKEDSKKASHILIKVKSKKSDKEGLDDKEAKQKAEEIQKEVSKDPSKFGEIAKKESMDTGSAKKDGELGYVLKGQTDKDFEKALFKLKDGEVSDVVKSSFGYHIIKADKPTDFNSEKQSLKEKLVDQKVQKNPKLLTDAYKDLLKEYDVDFKDRDIKSVVEDKILNPEKLKQGGAQGGQSGMSQ

>WP_162674950.1 foldase protein PrsA [Staphylococcus aureus]

MKMINKLIVPVTASALLLGACGASATDSKENTLISSKAGDVTVADTMKKIGKDQIANASFTEMLNKILADKYKNKVNDKKIDEQIEKMQKQYGGKDKFKKALQQQGLTADKYKENLRTAAYHKELLSDKIKISDSEIKEDSKKASHILIKVKSKKSDKEGLDDKEAKQKAEEIQKEVSKDPSKFGEIAKKESMDTGSAKKDGELGYVLKGQNDKDFEKALFKLKDGEVSDVVKSSFGYHIIKADKPTDFNSEKQSLKEKLVDQKVQKNPKLLTDAYKDLLKEYDVDFKDRDIKSVVEDKILNPEKLKQGGAQGGQSGMSQ

>HCG2516804.1 peptidylprolyl isomerase [Staphylococcus aureus]

MKMINKLIVPVTASALLLGACGASATDSKENTLISSKAGDVTVADTMKKIGKDQIANASFTEMLNKILADKYKNKVNDKKIDEQIEKMQKQYGGKDKFKKALQQQGLTADKYKENLRTAAYHKELLSDKIKISDSEIKEDSKKASHILIKVKSKKSDKEGLDDKEAKQKAEEIQKEVSKDPSKFGEIAKKESMDTGSAKKDGELGYVLKGQTDKDFEKALFKLKDGEVSDVVKSSFGYHIIKADKPTDFNSEKQSLKEKLVDQNVQKNPKLLTDAYKDLLKEYDVDFKDRDIKSVVEDKILNPEKLKQGGAQGGQSGMSQ

>HCX9095831.1 peptidylprolyl isomerase [Staphylococcus aureus]

MKMINKLIVPVTASALLLGACGTSATDSKENTLISSKAGDVTVADTMKKIGKDQIANASFTEMLNKILADKYKNKVNDKKIDEQIEKMQKQYGGKDKFKKALQQQGLTADKYKENLRTAAYHKELLSDKIKISDSEIKEDSKKASHILIKVKSKKSDKEGLDDKEAKQKAEEIQKEVSKDPSKFGEIAKKESMDTGSAKKDGELGYVLKGQTDKDFEKALFKLKDGEVSDVVKSSFGYHIIKADKPTDFNSEKQSLKEKLVDQKVQKNPKLLTDAYKDLLKEYDVDFKDRDIKSVVEDKILNPEKLKQGGAQGGQSGMSQ

>WP_103144839.1 foldase protein PrsA [Staphylococcus aureus]

MKMINKLIVPVTASALLLGACGASATDSKENTLISSKAGDVTVADTMKKIGKDQIANASFTEMLNKILADKHKNKVNDKKIDEQIEKMQKQYGGKDKFKKALQQQGLTADKYKENLRTAAYHKELLSDKIKISDSEIKEDSKKASHILIKVKSKKSDKEGLDDKEAKQKAEEIQKEVSKDPSKFGEIAKKESMDTGSAKKDGELGYVLKGQTDKDFEKALFKLKDGEVSDVVKSSFGYHIIKADKPTDFNSEKQSLKEKLVDQKVQKNPKLLTDAYKDLLKEYDVDFKDRDIKSVVEDKILNPEKLKQGGAQGGQSGMSQ

>HDC6369180.1 peptidylprolyl isomerase [Staphylococcus aureus]

MKMINKLIVPVTASTLLLGACGASATDSKENTLISSKAGDVTVADTMKKIGKDQIANASFTEMLNKILADKYKNKVNDKKIDEQIEKMQKQYGGKDKFKKALQQQGLTADKYKENLRTAAYHKELLSDKIKISDSEIKEDSKKASHILIKVKSKKSDKEGLDDKEAKQKAEEIQKEVSKDPSKFGEIAKKESMDTGSAKKDGELGYVLKGQTDKDFEKALFKLKDGEVSDVVKSSFGYHIIKADKPTDFNSEKQSLKEKLVDQKVQKNPKLLTDAYKDLLKEYDVDFKDRDIKSVVEDKILNPEKLKQGGAQGGQSGMSQ

>HCU6957441.1 peptidylprolyl isomerase [Staphylococcus aureus]

MKMINKLIVPVTASALLLGACGASATDSKENTLISSKAGDVTVADTMKKIGKDQIANASFTEMLNKILADKYKNKVNDKKIDEQIEKMQKQYGGKDKFKKALQQQGLTADKYKENLRTAAYHKELLSDKIKISDSEIKEDSKKASHILIKVKSKKSDKEGLDDKEAKQKAEEIQKEVSKDPSKFGEIAKKESMDAGSAKKDGELGYVLKGQTDKDFEKALFKLKDGEVSDVVKSSFGYHIIKADKPTDFNSEKQSLKEKLVDQKVQKNPKLLTDAYKDLLKEYDVDFKDRDIKSVVEDKILNPEKLKQGGAQGGQSGMSQ

>WP_072499084.1 foldase protein PrsA [Staphylococcus aureus]

MKMINKLIVPVTASALLLGACGASATDSKENTLISSKAGDVTVADTMKKIGKDQIANASFTEMLNKILADKYKNKVNDKKIDEQIEKMQKQYGGKDKFEKALQQQGLTADKYKENLRTAAYHKELLSDKIKISVSEIKEDSKKASHILIKVKSKKSDKEGLDDKEAKQKAEEIQKEVSKDPSKFGEIAKKESMDTGSAKKDGELGYVLKGQTDKDFEKALFKLKDGEVSDVVKSSFGYHIIKADKPTDFNSEKQSLKEKLVDQKVQKNPKLLTDAYKDLLKEYDVDFKDRDIKSVVEDKILNPEKLKQGGAKGGQSGMSQ

>HCT1677610.1 peptidylprolyl isomerase [Staphylococcus aureus]

MKMINKLIVPVTASALLLGACGASATDSKENTLISSKAGDVTVADTMKKIGKDQIANASFTEMLNKILADKYKNKVNDKKIDEQIEKMQKQYGGKDKFKKALQQQGLTADKYKENLRTAAYHKELLSDKIKISDSEIKEDSKKASHILIKVKSKKSDKEGLDDKEAKQKAEEIQKEVSKDPSKFGEIAKKESMDTGSAKKDGELGYVLKGQTDKDFEKALFKLKDGEVSDVVKSNFGYHIIKADKPTDFNSEKQSLKEKLVDQKVQKNPKLLTDAYKDLLKEYDADFKDRDIKSVVEDKILNPEKLKQGGAQGGQSGMSQ

>HDJ6707507.1 peptidylprolyl isomerase [Staphylococcus aureus]

LLGACGASATDSKENTLISSKAGDVTVADTMKKIGKDQIANASFTEMLNKILADKYKNKVNDKKIDEQIEKMQKQYGGKDKFEKALQQQGLTADKYKENLRTAAYHKELLSDKIKISDSEIKEDSKKASHILIKVKSKKSDKEGLDDKEAKQKAEEIQKEVSKDPSKFGEIAKKESMDTGSAKKDGELGYVLKGQTDKDFEKALFKLKDGEVSDVVKSSFGYHIIKADKPTDFNSEKQSLKEKLVDQKVQKNPKLLTDAYKDLLKEYDVDFKDRDIKSVVEDKILNPEKLKQGGAQGGQSGMSQ

>HEI5355819.1 peptidylprolyl isomerase [Staphylococcus aureus]

MKMINKLIVPVTASALLLGACGASATDSKENTLISSKAGDVTVADTMKKIGKDQIANASFTEMLNKILADKYKNKVNDKKIDEQIEKMQKQYGGKDKFKKALQQQGLTADKYKENLRTAAYHKELLSDKIKISDSEIKEDSKKASHILIKVKSKKSDKEGLDDKEAKQKAEEIQKEVSKDPSKFGEIAKKESMDTGSAKKDGELGYVLKGQTDKDFEKALFKLKDSEVSDVVKSSFGYHIIKADKPTDFNSEKQSLKEKLVDQKVQKNPKLLTDAYKDLLKEYDVDFKDRDIKSVVEDKILNPEKLKQGGAQGGQSGMSQ

>WP_031928765.1 foldase protein PrsA [Staphylococcus aureus]

MKMINKLIVPVTASALLLGACGASATDSKENTLISSKAGDVTVADTMKKIGKDQIANASFTEMLNKILADKYKYKNKVNDKKIDEQIEKMQKQYGGKDKFEKALQQQGLTADKYKENLRTAAYHKELLSDKIKISDSEIKEDSKKASHILIKVKSKKSDKEGLDDKEAKQKAEEIQKEVSKDPSKFGEIAKKESMDTGSAKKDGELGYVLKGQTDKDFEKALFKLKDGEVSDVVKSSFGYHIIKADKPTDFNSEKQSLKEKLVDQKVQKNPKLLTDAYKDLLKEYDVDFKDRDIKSVVEDKILNPEKLKQGGAQGGQSGMSQ

>WP_148871352.1 foldase protein PrsA [Staphylococcus aureus]

MKMINKLIVPVTASALLLGACGASATDSKENTLISSKAGDVTVADTMKKIGKDQIANASFTEMLNKILADKYKNKVNDKKIDEQIEKMQKQYGGKDKFKKALQQQGLTADKYKENLRTAAYHKELLSDKIKISDSEIKEDSKKASHILIKVKSKKSDKEGLDDKEAKQKAEEIQKEVSKDPSKFGEIAKKESMDTGSAKKDGELGYVLKGQTDKDFEKALFKLKDGEVSDVVKSSFGYHIIKADKPTDFNSEKQSLKEKLVDQKVQKIPKLLTDAYKDLLKEYDVDFKDRDIKSVVEDKILNPEKLKQGGAQGGQSGMSQ

>HDI7744902.1 peptidylprolyl isomerase [Staphylococcus aureus]

MKMINKLIVPVTASALLLGACGASATDSKENTLISSKAGDVTVADTMKKIGKDQIANASFTEMLNKILADKYKNKVNDKKIDEQIEKMQKQYGGKDKFKKALQQQGLTADKYKENLRTAAYHKELLSDKIKISDSEIKEDSKKASHILIKVKSKKSDKEGLDDKEAKQKAEEIQKEVSKDPSKFGEIAKKESMDTGSAKKDGELGYVLKGQTDKDFEKALFKLKDDEVSDVVKSSFGYHIIKADKPTDFNSEKQSLKEKLVDQKVQKNPKLLTDAYKDLLKEYDVDFKDRDIKSVVEDKILNPEKLKQGGAQGGQSGMSQ

>HDH3932424.1 peptidylprolyl isomerase [Staphylococcus aureus]

SATDSKENTLISSKAGDVTVADTMKKIGKDQIANASFTEMLNKILADKYKNKVNDKKIDEQIEKMQKQYGGKDKFEKALQQQGLTADKYKENLRTAAYHKELLSDKIKISDSEIKEDSKKASHILIKVKSKKSDKEGLDDKEAKQKAEEIQKEVSKDPSKFGEIAKKESMDTGSAKKDGELGYVLKGQTDKDFEKALFKLKDGEVSDVVKSSFGYHIIKADKPTDFNSEKQSLKEKLVDQKVQKNPKLLTDAYKDLLKEYDVDFKDRDIKSVVEDKILNPEKLKQGGAQGGQSGMSQ

>WP_031903039.1 foldase protein PrsA [Staphylococcus aureus]

MKMINKLIVPVTASALLLGACGACGACATDSKENTLISSKAGDVTVADTMKKIGKDQIANASFTEMLNKILADKYKNKVNDKKIDEQIEKMQKQYGGKDKFEKALQQQGLTADKYKENLRTAAYHKELLSDKIKISDSEIKEDSKKASHILIKVKSKKSDKEGLDDKEAKQKAEEIQKEVSKDPSKFGEIAKKESMDTGSAKKDGELGYVLKGQTDKDFEKALFKLKDGEVSDVVKSSFGYHIIKADKPTDFNSEKQSLKEKLVDQKVQKNPKLLTDAYKDLLKEYDVDFKDRDIKSVVEDKILNPEKLKQGGAQGGQSGMSQ

>HDJ1949577.1 peptidylprolyl isomerase [Staphylococcus aureus]

NTLISSKAGDVTVADTMKKIGKDQIANASFTEMLNKILADKYKNKVNDKKIDEQIEKMQKQYGGKDKFEKALQQQGLTADKYKENLRTAAYHKELLSDKIKISDSEIKEDSKKASHILIKVKSKKSDKEGLDDKEAKQKAEEIQKEVSKDPSKFGEIAKKESMDTGSAKKDGELGYVLKGQTDKDFEKALFKLKDGEVSDVVKSSFGYHIIKADKPTDFNSEKQSLKEKLVDQKVQKNPKLLTDAYKDLLKEYDVDFKDRDIKSVVEDKILNPEKLKQGGAQGGQSGMSQ

>HDC7820189.1 peptidylprolyl isomerase [Staphylococcus aureus]

TDSKENTLISSKAGDVTVADTMKKIGKDQIANASFTEMLNKILADKYKNKVNDKKIDEQIEKMQKQYGGKDKFEKALQQQGLTADKYKENLRTAAYHKELLSDKIKISDSEIKEDSKKASHILIKVKSKKSDKEGLDDKEAKQKAEEIQKEVSKDPSKFGEIAKKESMDTGSAKKDGELGYVLKGQTDKDFEKALFKLKDGEVSDVVKSSFGYHIIKADKPTDFNSEKQSLKEKLVDQKVQKNPKLLTDAYKDLLKEYDVDFKDRDIKSVVEDKILNPEKLKQGGAQGGQSGMSQ

>WP_188348865.1 peptidylprolyl isomerase [Staphylococcus aureus]

MKMINKLIVPVTASALLLGACGASATDSKENTLISSKAGDVTVADTMKKIGKDQIANASFTEMLNKILADKNKVNDKKIDEQIEKMQKQYGGKDKFEKALQQQGLTADKYKENLRTAAYHKELLSDKIKISDSEIKEDSKKASHILIKVKSKKSDKEGLDDKEAKQKAEEIQKEVSKDPSKFGEIAKKESMDTGSAKKDGELGYVLKGQTDKDFEKALFKLKDGEVSDVVKSSFGYHIIKADKPTDFNSEKQSLKEKLVDQKVQKNPKLLTDAYKDLLKEYDVDFKDRDIKSVVEDKILNPEKLKQGGAQGGQSGMSQ

>TXO13897.1 peptidylprolyl isomerase, partial [Staphylococcus aureus]

DQIANASFTEMLNKILADKYKNKVNDKKIDEQIEKMQKQYGGKDKFEKALQQQGLTADKYKENLRTAAYHKELLSDKIKISDSEIKEDSKKASHILIKVKSKKSDKEGLDDKEAKQKAEEIQKEVSKDPSKFGEIAKKESMDTGSAKKDGELGYVLKGQTDKDFEKALFKLKDGEVSDVVKSSFGYHIIKADKPTDFNSEKQSLKEKLVDQKVQKNPKLLTDAYKDLLKEYDVDFKDRDIKSVVEDKILNPEKLKQGGAQGGQSGMSQ

>CPN00812.1 Foldase protein PrsA precursor [Staphylococcus aureus]

MKKIGKDQIANASFTEMLNKILADKYKNKVNDKKIDEQIEKMQKQYGGKDKFEKALQQQGLTADKYKENLRTAAYHKELLSDKIKISDSEIKEDSKKASHILIKVKSKKSDKEGLDDKEAKQKAEEIQKEVSKDPSKFGEIAKKESMDTGSAKKDGELGYVLKGQTDKDFEKALFKLKDGEVSDVVKSSFGYHIIKADKPTDFNSEKQSLKEKLVDQKVQKNPKLLTDAYKDLLKEYDVDFKDRDIKSVVEDKILNPEKLKQGGAQGGQSGMSQ

>WP_308906064.1 peptidylprolyl isomerase [Staphylococcus aureus]

MKSLKKVMIPITASAVLLSACGNHATDSKENVLISSKAGDVKVEDVMKKIGKEQIANSSFEILLGKLLEKKYADKVDDKEIDKQIKDEQKQYGGKDQFESALKQQGMTLKDYKEQKKLQAYQKQMLMDKVKVSDKELKEDTKKASHILIKVKSDDKDKEGLSDKEAKAKAEKIQKEVKNNPDKFGEIAKKESMDEASAKKDGSLDYVIKGQMVKPFDKALFKLKDGEISDVVKSDYGYHIIKADQPTDFSSEKDKLKSQIIQNKVQKDPKILVEAYKDLLKEFNVDFKDRDIKKAVEDSILNPDKLKQQSQGGEGMQTP

>WP_258026993.1 foldase protein PrsA, partial [Staphylococcus aureus]

KKIGKDQIANASFTEMLNKILADKYKNKVNDKKIDEQIEKMQKQYGGKDKFEKALQQQGLTADKYKENLRTAAYHKELLSDKIKISDSEIKEDSKKASHILIKVKSKKSDKEGLDDKEAKQKAEEIQKEVSKDPSKFGEIAKKESMDTGSAKKDGELGYVLKGQTDKDFEKALFKLKDGEVSDVVKSSFGYHIIKADKPTDFNSEKQSLKEKLVDQKVQKNPKLLTDAYKDLLKEYDVDFKDRDIKSVVEDKILNPEKLKQGGAQGGQSGMSQ

>CXN24826.1 Foldase protein PrsA precursor [Staphylococcus aureus]

MLNKILADKYKNKVNDKKIDEQIEKMQKQYGGKDKFEKALQQQGLTADKYKENLRTAAYHKELLSDKIKISDSEIKEDSKKASHILIKVKSKKSDKEGLDDKEAKQKAEEIQKEVSKDPSKFGEIAKKESMDTGSAKKDGELGYVLKGQTDKDFEKALFKLKDGEVSDVVKSSFGYHIIKADKPTDFNSEKQSLKEKLVDQKVQKNPKLLTDAYKDLLKEYDVDFKDRDIKSVVEDKILNPEKLKQGGAQGGQSGMSQ

>NGB07064.1 peptidylprolyl isomerase [Staphylococcus aureus]

NKILADKYKNKVNDKKIDEQIEKMQKQYGGKDKFEKALQQQGLTADKYKENLRTAAYHKELLSDKIKISDSEIKEDSKKASHILIKVKSKKSDKEGLDDKEAKQKAEEIQKEVSKDPSKFGEIAKKESMDTGSAKKDGELGYVLKGQTDKDFEKALFKLKDGEVSDVVKSSFGYHIIKADKPTDFNSEKQSLKEKLVDQKVQKNPKLLTDAYKDLLKEYDVDFKDRDIKSVVEDKILNPEKLKQGGAQGGQSGMSQ

>NGC81347.1 peptidylprolyl isomerase [Staphylococcus aureus]

YKNKVNDKKIDEQIEKMQKQYGGKDKFEKALQQQGLTADKYKENLRTAAYHKELLSDKIKISDSEIKEDSKKASHILIKVKSTKSDKEGLDDKEAKQKAEEIQKEVSKDPSKFGEIAKKESMDTGSAKKDGELGYVLKGQTDKDFEKALFKLKDGEVSDVVKSSFGYHIIKADKPTDFNSEKQSLKEKLVDQKVQKNPKLLTDAYKDLLKEYDVDFKDRDIKSVVEDKILNPEKLKQGGAQGGQSGMSQ

>HAR5338381.1 peptidylprolyl isomerase [Staphylococcus aureus]

MKMINKLIVPVTASALLLGACGASATDSKENTLISSKAGDVTVADTMKKIGKDQIANASFTEMLNKILADKYKNKVNNKKIDEQIEKMQKQYGGKDKFEKALQQQGLTADKYKENLRTAAYHKELLSDKIKISDSEIKEDSKKASHILIKVKSKKSDKEGLDDKEAKQKAEEIQKEVSKDPSKFGEIAKKESMDTGSAKKDGELGYVLKGQTDKDFEKALFKLKDGEVSDVVKSSFGYHIIKADKPTDFNSEKQSLKEKLVDQKVQKNPKLLTDAYKDLLKEYDVDFKDRDIKSVVEDKILNPEKLKQGGAQGGQSGMSQ

>WP_154456241.1 foldase protein PrsA, partial [Staphylococcus aureus]

VNDKKIDEQIEKMQKQYGGKDKFEKALQQQGLTADKYKENLRTAAYHKELLSDKIKISDSEIKEDSKKASHILIKVKSKKSDKEGLDDKEAKQKAEEIQKEVSKDPSKFGEIAKKESMDTGSAKKDGELGYVLKGQTDKDFEKALFKLKDGEVSDVVKSSFGYHIIKADKPTDFNSEKQSLKEKLVDQKVQKNPKLLTDAYKDLLKEYDVDFKDRDIKSVVEDKILNPEKLKQGGAQGGQSGMSQ

>WP_111028029.1 foldase protein PrsA, partial [Staphylococcus aureus]

VNDKKIDEQIEKMQKQYGGKDKFKKALQQQGLTADKYKENLRTAAYHKELLSDKIKISDSEIKEDSKKASHILIKVKSKKSDKEGLDDKEAKQKAEEIQKEVSKDPSKFGEIAKKESMDTGSAKKDGELGYVLKGQTDKDFEKALFKLKDGEVSDVVKSSFGYHIIKADKPTDFNSEKQSLKEKLVDQKVQKNPKLLTDAYKDLLKEYDVDFKDRDIKSVVEDKILNPEKLKQGGAQGGQSGMSQ

>WP_271291197.1 foldase protein PrsA, partial [Staphylococcus aureus]

DKKIDEQIEKMQKQYGGKDKFEKALQQQGLTADKYKENLRTAAYHKELLSDKIKISDSEIKEDSKKASHILIKVKSKKSDKEGLDDKEAKQKAEEIQKEVSKDPSKFGEIAKKESMDTGSAKKDGELGYVLKGQTDKDFEKALFKLKDGEVSDVVKSSFGYHIIKADKPTDFNSEKQSLKEKLVDQKVQKNPKLLTDAYKDLLKEYDVDFKDRDIKSVVEDKILNPEKLKQGGAQGGQSGMSQ

>WP_063652494.1 foldase protein PrsA, partial [Staphylococcus aureus]

SGGKDKFEKALQQQGLTADKYKENLRTAAYHKELLSDKIKISDSEIKEDSKKASHILIKVKSKKSAKEGLDDKEAKQKAEEIQKEVSKDPSKFGKIAKKESMDTGSAKKDGELGYVLKGQTDKDFEKALFKLKDGEVSDVVKSSFGYHIIKADKPTDFNSEKQSLKEKLVDQKVQKNPKLLTDAYKDLLKEYDVDFKDRDIKSVVEDKILNPEKLKQGGAQGGQSGMSQ

>TXO02275.1 peptidylprolyl isomerase, partial [Staphylococcus aureus]

DEQIEKMQKQYGGKDKFEKALQQQGLTADKYKENLRTAAYHKELLSDKIKISDSEIKEDSKKASHILIKVKSKKSDKEGLDDKEAKQKAEEIQKEVAKDPSKFAEIAKKESMDTGSAKKDGELGYVLKGQTDKDFEKALFKLKDGEVSDVVKSSFGYHIIKADKPTDFNSEKQSLKEKLVDQKVQKNPKLLTDAYKDLLKEYDVDFKDRDIKSVVEDKILNPEKLKQGGAQGGQSGMSQ

>WP_101262441.1 foldase protein PrsA, partial [Staphylococcus aureus]

KIDEQIEKMQKQYGGKDKFEKALQQQGLTADKYKENLRTAAYHKELLSDKIKISDSEIKEDSKKASHILIKVKSKKSDKEGLDDKEAKQKAEEIQKEVSKDPSKFGEIAKKESMDTGSAKKDGELGYVLKGQTDKDFEKALFKLKDGEVSDVVKSSFGYHIIKADKPTDFNSEKQSLKEKLVDQKVQKNPKLLTDAYKDLLKEYDVDFKDRDIKSVVEDKILNPEKLKQGGAQGGQSGMSQ

>WP_101245020.1 foldase protein PrsA, partial [Staphylococcus aureus]

IDEQIEKMQKQYGGKDKFEKALQQQGLTADKYKENLRTAAYHKELLSDKIKISDSEIKEDSKKASHILIKVKSKKSDKEGLDDKEAKQKAEEIQKEVSKDPSKFGEIAKKESMDTGSAKKDGELGYVLKGQTDKDFEKALFKLKDGEVSDVVKSSFGYHIIKADKPTDFNSEKQSLKEKLVDQKVQKNPKLLTDAYKDLLKEYDVDFKDRDIKSVVEDKILNPEKLKQGGAQGGQSGMSQ

>WP_258026863.1 foldase protein PrsA, partial [Staphylococcus aureus]

DEQIEKMQKQYGGKDKFEKALQQQGLTADKYKENLRTAAYHKELLSDKIKISDSEIKEDSKKASHILIKVKSKKSDKEGLDDKEAKQKAEEIQKEVSKDPSKFGEIAKKESMDTGSAKKDGELGYVLKGQTDKDFEKALFKLKDGEVSDVVKSSFGYHIIKADKPTDFNSEKQSLKEKLVDQKVQKNPKLLTDAYKDLLKEYDVDFKDRDIKSVVEDKILNPEKLKQGGAQGGQSGMSQ

>HCX9211724.1 peptidylprolyl isomerase [Staphylococcus aureus]

KIDEQIEKMQKQYGGKDKFKKALQQQGLTADKYKENLRTAAYHKELLSDKIKISDSEIKEDSKKASHILIKVKSKKSDKEGLDDKEAKQKAEEIQKEVSKDPSKFGEIAKKESMDTGSAKKDGELGYVLKGQTDKDFEKALFKLKDGEVSDVVKSSFGYHIIKADKPTDFNSEKQSLKEKLVDQKVQKNPKLLTDAYKDLLKEYDVDFKDRDIKSVVEDKILNPEKLKQGGAQGGQSGMSQ

>HEI1482895.1 peptidylprolyl isomerase [Staphylococcus aureus]

KDKFEKALQQQGLTADKYKENLRTAAYHKELLSDKIKISDSEIKEDSKKASHILIKVKSKKSDKEGLDDKEAKQKAEEIQKEVSKDPSKFGEIAKKESMDTGSAKKDGELGYVLKGQTDKDFEKALFKLKDGEVSDVVKSSFGYHIIKADKPTDFNSEKQSLKEKLVDQKVQKNPKLLTDAYKDLLKEYDVDFKDRDIKSVVEDKILNPEKLKQGGAQGGQSGMSQ

>HDJ1844156.1 peptidylprolyl isomerase [Staphylococcus aureus]

QKQYGGKDKFEKALQQQGLTADKYKENLRTAAYHKELLSDKIKISDSEIKEDSKKASHILIKVKSKKSDKEGLDDKEAKQKAEEIQKEVSKDPSKFGEIAKKESMDTGSAKKDGELGYVLKGQTDKDFEKALFKLKDGEVSDVVKSSFGYHIIKADKPTDFNSEKQSLKEKLVDQKVQKNPKLLTDAYKDLLKEYDVDFKDRDIKSVVEDKILNPEKLKQGGAQGGQSGMSQ

>WP_218089241.1 foldase protein PrsA, partial [Staphylococcus aureus]

QYGGKDKFEKALQQQGLTADKYKENLRTAAYHKELLSDKIKISDSEIKEDSKKASHILIKVKSKKSDKEGLDDKEAKQKAEEIQKEVSKDPSKFGEIAKKESMDTGSAKKDGELGYVLKGQTDKDFEKALFKLKDGEVSDVVKSSFGYHIIKADKPTDFNSEKQSLKEKLVDQKVQKNPKLLTDAYKDLLKEYDVDFKDRDIKSVVEDKILNPEKLKQGGAQGGQSGMSQ

>HDP3194651.1 peptidylprolyl isomerase [Staphylococcus aureus]

KMQKQYGGKDKFEKALQQQGLTADKYKENLRTAAYHKELLSDKIKISDSEIKEDSKKASHILIKVKSKKSDKEGLDDKEAKQKAEEIQKEVSKDPSKFGEIAKKESMDTGSAKKDGELGYVLKGQTDKDFEKALFKLKDGEVSDVVKSSFGYHIIKADKPTDFNSEKQSLKEKLVDQKVQKNPKLLTDAYKDLLKEYDVDFKDRDIKSVVEDKILNPEKLKQGGAQGGQSGMSQ

>HCZ9114774.1 peptidylprolyl isomerase [Staphylococcus aureus]

EQIEKMQKQYGGKDKFEKALQQQGLTADKYKENLRTAAYHKELLSDKIKISDSEIKEDSKKASHILIKVKSKKSDKEGLDDKEAKQKAEEIQKEVSKDPSKFGEIAKKESMDTGSAKKDGELGYVLKGQTDKDFEKALFKLKDGEVSDVVKSSFGYHIIKADKPTDFNSEKQSLKEKLVDQKVQKNPKLLTDAYKDLLKEYDVDFKDRDIKSVVEDKILNPEKLKQGGAQGGQSGMSQ

>WP_256717281.1 foldase protein PrsA, partial [Staphylococcus aureus]

KQYGGKDKFEKALQQQGLTADKYKENLRTAAYHKELLSDKIKISDSEIKEDSKKASHILIKVKSKKSDKEGLDDKEAKQKAEEIQKEVSKDPSKFGEIAKKESMDTGSAKKDGELGYVLKGQTDKDFEKALFKLKDGEVSDVVKSSFGYHIIKADKPTDFNSEKQSLKEKLVDQKVQKNPKLLTDAYKDLLKEYDVDFKDRDIKSVVEDKILNPEKLKQGGAQGGQSGMSQ

>WP_169428016.1 foldase protein PrsA, partial [Staphylococcus aureus]

IEKMQKQYGGKDKFEKALQQQGLTADKYKENLRTAAYHKELLSDKIKISDSEIKEDSKKASHILIKVKSKKSDKEGLDDKEAKQKAEEIQKEVSKDPSKFGEIAKKESMDTGSAKKDGELGYVLKGQTDKDFEKALFKLKDGEVSDVVKSSFGYHIIKADKPTDFNSEKQSLKEKLVDQKVQKNPKLLTDAYKDLLKEYDVDFKDRDIKSVVEDKILNPEKLKQGGAQGGQSGMSQ

>WP_103143781.1 peptidylprolyl isomerase, partial [Staphylococcus aureus]

DKFKKALQQQGLTADKYKENLRTAAYHKELLSDKIKISDSEIKEDSKKASHILIKVKSKKSDKEGLDDKEAKQKAEEIQKEVSKDPSKFGEIAKKESMDTGSAKKDGELGYVLKGQTDKDFEKALFKLKDGEVSDVVKSSFGYHIIKADKPTDFNSEKQSLKEKLVDQKVQKNPKLLTDAYKDLLKEYDVDFKDRDIKSVVEDKILNPEKLKQGGAQGGQSGMSQ

>WP_103144873.1 peptidylprolyl isomerase, partial [Staphylococcus aureus]

GKDKFKKALQQQGLTADKYKENLRTAAYHKELLSDKIKISDSEIKEDSKKASHILIKVKSKKSDKEGLDDKEAKQKAEEIQKEVSKDPSKFGEIAKKESMDTGSAKKDGELGYVLKGQTDKDFEKALFKLKDGEVSDVVKSSFGYHIIKADKPTDFNSEKQSLKEKLVDQKVQKNPKLLTDAYKDLLKEYDVDFKDRDIKSVVEDKILNPEKLKQGGAQGGQSGMSQ

>HDH6142715.1 peptidylprolyl isomerase [Staphylococcus aureus]

QYGGKDKFKKALQQQGLTADKYKENLRTAAYHKELLSDKIKISDSEIKEDSKKASHILIKVKSKKSDKEGLDDKEAKQKAEEIQKEVSKDPSKFGEIAKKESMDTGSAKKDGELGYVLKGQTDKDFEKALFKLKDGEVSDVVKSSFGYHIIKADKPTDFNSEKQSLKEKLVDQKVQKNPKLLTDAYKDLLKEYDVDFKDRDIKSVVEDKILNPEKLKQGGAQGGQSGMSQ

>HBI0819640.1 peptidylprolyl isomerase [Staphylococcus aureus]

GGKDKFKKALQQQGLTADKYKENLRTAAYHKELLSDKIKISDSEIKEDSKKASHILIKVKSKKSDKEGLDDKEAKQKAEEIQKEVSKDPSKFGEIAKKESMDTGSAKKDGELGYVLKGQTDKDFEKALFKLKDGEVSDVVKSSFGYHIIKADKPTDFNSEKQSLKEKLVDQKVQKNPKLLTDAYKDLLKEYDVDFKDRDIKSVVEDKILNPEKLKQGGAQGGQSGMSQ

>HDH2420163.1 peptidylprolyl isomerase [Staphylococcus aureus]

EKALQQQGLTADKYKENLRTAAYHKELLSDKIKISDSEIKEDSKKASHILIKVKSKKSDKEGLDDKEAKQKAEEIQKEVAKDPSKFAEIAKKESMDTGSAKKDGELGYVLKGQTDKDFEKALFKLKDGEVSDVVKSSFGYHIIKADKPTDFNSEKQSLKEKLVDQKVQKNPKLLTDAYKDLLKEYDVDFKDRDIKSVVEDKILNPEKLKQGGAQGGQSGMSQ

>WP_162648617.1 peptidylprolyl isomerase, partial [Staphylococcus aureus]

EKALQQQGLTADKYKENLRTAAYHKELLSDKIKISDSEIKEDSKKASHILIKVKSKKSDKEGLDDKEAKQKAEEIQKEVSKDPSKFGEIAKKESMDTGSAKKDGELGYVLKGQTDKDFEKALFKLKDGEVSDVVKSSFGYHIIKADKPTDFNSEKQSLKEKLVDQKVQKNPKLLTDAYKDLLKEYDVDFKDRDIKSVVEDKILNPEKLKQGGAQGGQSGMSQ

>WP_237763407.1 peptidylprolyl isomerase, partial [Staphylococcus aureus]

LQQQGLTADKYKENLRTAAYHKELLSDKIKISDSEIKEDSKKASHILIKVKSKKSDKEGLDDKEAKQKAEEIQKEVSKDPSKFGEIAKKESMDTGSAKKDGELGYVLKGQTDKDFEKALFKLKDGEVSDVVKSSFGYHIIKADKPTDFNSEKQSLKEKLVDQKVQKNPKLLTDAYKDLLKEYDVDFKDRDIKSVVEDKILNPEKLKQGGAQGGQSGMSQ

>HCX1266855.1 peptidylprolyl isomerase [Staphylococcus aureus]

ALQQQGLTADKYKENLRTAAYHKELLSDKIKISDSEIKEDSKKASHILIKVKSKKSDKEGLDDKEAKQKAEEIQKEVSKDPSKFGEIAKKESMDTGSAKKDGELGYVLKGQTDKDFEKALFKLKDGEVSDVVKSSFGYHIIKADKPTDFNSEKQSLKEKLVDQKVQKNPKLLTDAYKDLLKEYDVDFKDRDIKSVVEDKILNPEKLKQGGAQGGQSGMSQ

>WP_114647754.1 peptidylprolyl isomerase, partial [Staphylococcus aureus]

KALQQQGLTADKYKENLRTAAYHKELLSDKIKISDSEIKEDSKKASHILIKVKSKKSDKEGLDDKEAKQKAEEIQKEVSKDPSKFGEIAKKESMDTGSAKKDGELGYVLKGQTDKDFEKALFKLKDGEVSDVVKSSFGYHIIKADKPTDFNSEKQSLKEKLVDQKVQKNPKLLTDAYKDLLKEYDVDFKDRDIKSVVEDKILNPEKLKQGGAQGGQSGMSQ
